# Discovery of a novel hybrid coumarin-hydroxamate conjugate targeting the HDAC1-Sp1-FOSL2 signaling axis for breast cancer therapy

**DOI:** 10.1186/s12964-024-01733-4

**Published:** 2024-07-15

**Authors:** Sujie Zhu, Wenjing Zhu, Kaihua Zhao, Jie Yu, Wenxia Lu, Rui Zhou, Shule Fan, Weikaixin Kong, Feifei Yang, Peipei Shan

**Affiliations:** 1 Institute of Translational Medicine, College of Medicine, The Affiliated Hospital of Qingdao University, Qingdao University, Qingdao, 266021 China; 2 Clinical Research Center, Qingdao Municipal Hospital, Qingdao, 266071 China; 3 Qingdao Central Hospital, University of Health and Rehabilitation Sciences (Qingdao Central Hospital), Qingdao, 266042 China; 4 School of Biological Science and Technology, University of Jinan, Jinan, 250022 China; 5 Department of Marine Bio-Pharmacology, College of Food Science and Technology, Shanghai Ocean University, Shanghai, 201306 China; 6 Department of Molecular and Cellular Pharmacology, School of Pharmaceutical Sciences, Peking University Health Science Center, Beijing, 100191 China; 7 Institute for Molecular Medicine Finland (FIMM), HiLIFE, University of Helsinki, Helsinki, 00250 Finland; 8 Institute Sanqu Technology (Hangzhou) Co., Ltd., Hangzhou, China

**Keywords:** Breast cancer, Metastasis, Tumor growth, Histone deacetylases inhibitor, Coumarins, FOSL2

## Abstract

**Background:**

Breast cancer is one of the most lethal cancers in women. Despite significant advances in the diagnosis and treatment of breast cancer, many patients still succumb to this disease, and thus, novel effective treatments are urgently needed. Natural product coumarin has been broadly investigated since it reveals various biological properties in the medicinal field. Accumulating evidence indicates that histone deacetylase inhibitors (HDACIs) are promising novel anti-breast cancer agents. However, most current HDACIs exhibit only moderate effects against solid tumors and are associated with severe side effects. Thus, to develop more effective HDACIs for breast cancer therapy, hydroxamate of HDACIs was linked to coumarin core, and coumarin-hydroxamate hybrids were designed and synthesized.

**Methods:**

A substituted coumarin moiety was incorporated into the classic hydroxamate HDACIs by the pharmacophore fusion strategy. ZN444B was identified by using the HDACI screening kit and cell viability assay. Molecular docking was performed to explore the binding mode of ZN444B with HDAC1. Western blot, immunofluorescent staining, cell viability, colony formation and cell migration and flow cytometry assays were used to analyze the anti-breast cancer effects of ZN444B in vitro. Orthotopic studies in mouse models were applied for preclinical evaluation of efficacy and toxicity in vivo. Proteomic analysis, dual-luciferase reporter assay, chromatin immunoprecipitation, co-immunoprecipitation, immunofluorescent staining assays along with immunohistochemical (IHC) analysis were used to elucidate the molecular basis of the actions of ZN444B.

**Results:**

We synthesized and identified a novel coumarin-hydroxamate conjugate, ZN444B which possesses promising anti-breast cancer activity both in vitro and in vivo. A molecular docking model showed that ZN444B binds to HDAC1 with high affinity. Further mechanistic studies revealed that ZN444B specifically decreases FOS-like antigen 2 (FOSL2) mRNA levels by inhibiting the deacetylase activity of HDAC1 on Sp1 at K703 and abrogates the binding ability of Sp1 to the *FOSL2* promoter. Furthermore, FOSL2 expression positively correlates with breast cancer progression and metastasis. Silencing FOSL2 expression decreases the sensitivity of breast cancer cells to ZN444B treatment. In addition, ZN444B shows no systemic toxicity in mice.

**Conclusions:**

Our findings highlight the potential of FOSL2 as a new biomarker and therapeutic target for breast cancer and that targeting the HDAC1-Sp1-FOSL2 signaling axis with ZN444B may be a promising therapeutic strategy for breast cancer.

**Supplementary Information:**

The online version contains supplementary material available at 10.1186/s12964-024-01733-4.

## Background

Breast cancer, with high mortality worldwide, is the most prevalent cancer among women. Despite significant advances in the diagnosis and treatment of breast cancer, it still has a high incidence and mortality and thus represents a life-threatening disease in women [1]. Breast cancer is a complex heterogeneous disease that can be histologically classified into hormone-receptor-positive, human epidermal growth factor (EGF) receptor-2-overexpressing, and triple-negative breast cancer [2, 3]. Recently, encouraging results have been observed with endocrine therapy and human-EGF-receptor-2-targeted therapy [4–6]. However, severe side effects were observed after endocrine therapy and chemotherapy. Moreover, many patients develop resistance to chemotherapeutic agents and eventually experience treatment failure. Therefore, developing more effective therapeutic drugs and exploring potential molecular therapeutic targets for breast cancer are urgently needed.

Histone deacetylases (HDACs) are clinically validated epigenetic drug targets for breast cancer treatment, and five histone deacetylases inhibitors (HDACIs) (vorinostat (SAHA), romidepsin (FK228), belinostat (PXD101), panobinostat (LBH589), and chidamide (CS055)) have been approved for the treatment of cancer [7–12]. However, most current HDACIs have shown limited effectiveness in solid tumors along with toxic side effects and drug resistance [13–15]. Therefore, it is necessary to develop novel HDACIs for breast cancer therapeutics with improved activity. The common pharmacophores of classical HDACI consist of three groups, including a hydrophobic cap group (CAP), a linker domain (Linker) and a zinc-binding group (ZBG) [16]. ZBG acts as the chelating motif for Zn^2+^ in the active site of HDACs, responsible for the potency of inhibitors. The CAP group mediates the interaction with the amino acids at the rim of the enzyme. Thus, structure modification on the CAP region was an effective strategy to design novel inhibitors with improved potency and selectivity [17–20].

Coumarin, a natural product, is an oxygen-containing heterocyclic ring compound. In recent years, numerous studies have demonstrated that coumarins and their derivatives exhibit diverse pharmacological properties, such as anti-tumor, anti-oxidant, anti-coagulant, and anti-microbial effects [21]. Coumarin derivatives easily interact with a range of enzymes and receptors in breast cancer cells [22]. Studies have shown that coumarin hybrids exhibit strong efficacy against breast cancer by binding to different biological targets [23, 24]. Researchers are now highly interested in investigating the therapeutic potential of coumarin hybrids in breast cancer [25]. In addition, a former study had reported that a coumarin derivative, 6,7-dihydroxycoumarin exerts potential anti-oral squamous cancer effect via suppressing the expression of Sp1 [26]. Therefore, in this study, we choose coumarin moiety as a cap group of HDACIs to develop new potential drugs for breast cancer therapy.

Considering coumarin have demonstrated promising biological profiles with low toxicity and hydroxamate as the most active ZBG, we introduced coumarin to the CAP group of HDACIs, and coumarin-hydroxamate hybrid ZN444B with best anti-proliferative effect on breast cancer cells was designed and synthesized. The mechanism of action of this coumarin-hydroxamate conjugate and potential therapeutic targets for breast cancer warrant further exploration.

In this study, we identified a novel hybrid coumarin-hydroxamate conjugate, ZN444B, which significantly inhibits breast cancer cell growth and metastasis both in vitro and in vivo. Further mechanistic studies revealed that ZN444B inhibits Fos-related antigen-2 (FOSL2) expression in breast cancer cells by inducing the disassociation of HDAC1 and Sp1, inhibits the deacetylase activity of HDAC1 on Sp1 at K703, and abrogates the binding ability of Sp1 to the *FOSL2* promoter. This subsequently inhibits FOSL2 expression and thus blocks cell growth and metastasis. We demonstrate for the first time that FOSL2 expression positively correlates with the progression and metastasis of breast cancer. Our findings suggest that FOSL2 can be explored as a new biomarker for breast cancer and that targeting the HDAC1-Sp1-FOSL2 signaling axis with ZN444B may represent a novel option for the treatment of breast cancer.

## Materials and methods

### Cell lines, plasmids and reagents

MDA-MB231 (RRID: CRL_12532, ATCC), 4T1 (RRID: CRL_2539, ATCC), BT549 (RRID: CAL_148, ATCC) and HEK293T (RRID: CVCL_0063, ATCC) cells were obtained from the American Type Culture Collection. MDA-MB231 cells were maintained in DMEM (Cat# MA0212, Meilunbio) with 10% (v/v) fetal bovine serum. 4T1 and BT549 cells were maintained in RPMI 1640 (Cat# MA0214, Meilunbio) medium with 10% (v/v) fetal bovine serum (Cat# PWL0001, Meilunbio). All cells were maintained at 37 °C under a humidified 5% CO_2_ incubator. All cell lines tested negative for Mycoplasma contamination (Mycoplasma Detection Kit, TransGen Biotech). The cells were passaged for fewer than 6 months after resuscitation and were authenticated prior to use by short tandem repeat profiling. *HDAC1*-knockdown, *FOSL2* or vector control stable cell lines were established by transient transfection and selected with puromycin. *FOSL2* and *Sp1* knockdown-rescue stable cell lines were established by transfection of RNAi-resistant FOSL2 and Sp1 expressing plasmid and selected with hygromycin. The human FOSL2, Sp1 and HDAC1 expression plasmids were amplified from human cDNA and inserted to pCDNA 3.0 vector. FOSL2 antibody was purchased from Absin (Cat# abs136586). Antibody against PCNA was purchased from Abcam (Cat# Ab13110). Antibodies against Actin (Cat# A5316) and Flag (Cat# F1804) were purchased from Sigma. Antibodies against Sp1 (Cat# 5931), acetyl-lysine antibody (Cat# 9441), acetyl-histone H3 (Cat# 9649 S), acetyl-histone H4 (Cat# 8647 S), Bcl-2 (Cat# 3498), Bcl-XL (Cat# 2792), E-cadherin (Cat# 4065), ZEB1 (Cat# 3396), cleaved PARP (Cat# 9544P), Histone-H3 (Cat# 4499), EphA2 (Cat# 6997), P21 (Cat# 64016) and HDAC1 (Cat# 2062) were purchased from Cell Signaling Technology. Antibody against Vimentin was purchased from Dako (Cat# M0725). DMSO, puromycin and hygromycin were obtained from Sigma. Protein A/G agarose was purchased from ThermoFisher Scientific (Cat# 20423). Matrigel was purchased from BD Bioscience. Compounds were dissolved in DMSO and stored at -20 °C as small aliquots.

### Animal experiments

BALB/c, Female, 6-8-week-old mice were obtained from the Institute of Laboratory Animal Science of Chinese Academy of Medical Sciences (Beijing, China). All animal experiments were performed according to guidelines approved by the Institutional Animal Care and performed in accordance with the guidelines for Animal Experimentation of Qingdao University (Qingdao, Shandong, P.R. China) and approved by the Committee for Animal Experimentation. 4T1 cells (1 × 10^5^) were injected into the mammary fat pad of female BALB/c mice. Treatment began after the tumor nodules grew to 100 mm^3^. Tumor-bearing BALB/c mice were randomly assigned to three groups (*n* = 5 per group) and received IP injection of ZN444B (30 mg/kg/day), SAHA (30 mg/kg/day), and the control group were injected with PBS. Primary tumor size was measured every week and tumor volume was calculated according to the formula: V = L×W^2^ × 0.52, where L and W refer to length and width, respectively. At the end of the experiment, the mice were euthanized by CO_2_. The primary tumors were removed from mice after administration of ZN444B or SAHA for 28 days. Primary tumors weight in each group was measured, and tumors were lysed and applied to immunoblotting with the indicated antibodies, or fixed and paraffin embedded by IHC analysis, respectively. To examine the anti-metastasis activity of ZN444B in vivo. 4T1-luc cells (1 × 10^5^) were injected into the mammary fat pad of female BALB/c mice, 6-8-week-old (Institute of Laboratory Animal Science of Chinese Academy of Medical Sciences (Beijing, China)). Mice were divided into 2 groups (*n* = 5 per group) on day 7 after tumor cell implantation and received IP injection of ZN444B (30 mg/kg/day) and control group were injected with PBS. Real-time images on treatment day 0, day 10, day 30 and day 60 were acquired using the Xenogen IVIS. Ex vivo bioluminescence images were obtained in each group to determine the effects of ZN444B against distant metastasis. Metastasis incidence in distant organ was quantified. Lung metastases were manually counted using a dissecting microscope by three individuals who do not have personal biases with the experiment. Primary tumors were lysed and applied to immunoblotting with the indicated antibodies. Another independent animal experiment was performed to determine survival curve.

Another independent animal experiment (*n* = 6 per group) was performed by using 12 female BALB/c mice for the toxicity study.

### Assay for inhibiting each HDAC isoform

The protease-coupled assay was used to measure HDACs enzyme activity in vitro. After incubating various concentrations of compounds with recombinant HDAC (BPS Biosciences, US) for 15 min at room temperature, trypsin and Ac-peptide-AMC substrates were added to Tris-based assay buffer to start the reaction. In SynergyMx (BioTek, US), fluorescent AMC released from substrate was measured using filter sets with excitation = 355 nm and emission = 460 nm. Software called GraphPad Prism (California, US) was used to calculate IC_50_values.

### Patients tissue collection

Patients were informed before the surgery and agreed by written consent to donate tissues. All experiments were performed in accordance with the IRB committee’s regulations of Qingdao University on human subject research. All procedures were performed at the Affiliated Hospital of Qingdao University (Permission number QDU-HEC-2,022,051).

### Immunoprecipitation and Western blotting

Cells were lysed in lysis buffer (50 mM Tris-HCl pH 8.0, 5 mM EDTA, 150 mM NaCl, 0.5% NP-40, 1 mM PMSF), centrifuged for 5 min at 10,000 g, and the insoluble debris was discarded. Cell lysates were further analyzed with SDS-PAGE and western blotting. For co-immunoprecipitation, cell lysates were immunoprecipitated with protein A/G agarose plus anti-HDAC1 or Sp1 antibodies for 4–6 h at 4 °C. The beads were washed extensively with lysis buffer, boiled in SDS sample buffer, fractionated by SDS-PAGE, and analyzed by western blotting using specific antibodies.

### Immunofluorescence staining

Immunofluorescence was operated as previously described [27]. Briefly, cells were fixed in 4% formaldehyde for 10 min, incubated with primary antibody for 2 h at room temperature or overnight at 4 °C, followed by a 2 h exposure to the fluorescently conjugated secondary antibody at room temperature. Immunofluorescence was visualized by fluorescence microscopy.

### Immunohistochemical staining

IHC staining analysis was performed as previously reported [28]. Antibodies against FOSL2, Ac-H3 and Ac-H4 were used for immunohistochemistry staining, respectively. Images were obtained with Leica microscope (Leica, DM4000b).

### Proteomics (LC-MS/MS) analyses

Proteomics assay was performed as previously reported [27]. Briefly, breast cancer cells were treated with ZN444B or PBS for 24 h, then cells were collected for proteomics analyses. For each sample, ~ 2 µg peptides were separated and analyzed with a nano-UPLC coupled to Q-Exactive mass spectrometry (Thermo Finnigan). Separation was performed using a reversed-phasecolumn. Mobile phases were H_2_O with 0.1% FA, 2% ACN (phase A) and 80% ACN, 0.1% FA. Separation of sample was executed with a 120 min gradient at 300 nL/min flow rate. Gradient B: 8–30% for 92 min, 30–40% for 20 min, 40 to 100% for 2 min, 100% for 2 min, 100–2% for 2 min and 2% for 2 min. Data dependent acquisition was performed in profile and positive mode with Orbitrap analyzer at a resolution of 70,000 (200 m/z) and m/z range of 350–1600 for MS1; For MS2, the resolution was set to 17,500 with a dynamic first mass. The dynamic exclusion time window was 30 s.

### HDAC inhibitory assay

HDACI activity assay was performed using the HDACI screening kit (BioVision, Inc, Cat# K340-100) according to the manufacturer’s instruction. Briefly, HeLa nuclear extract or MDA-MB231 cell lysate was incubated with the candidate HDACIs or SAHA in the presence of HDAC fluorometric substrate containing an acetylated lysine side chain at 37 °C for 1 h. The lysine developer was then added to stop the reaction and the fluorescence was measured. The fluorescence intensities were determined on a fluorometer with 350–380 nm excitation and 440–460 nm emission. Three independent experiments with triplicate were carried out.

### Cell transfection assay

Breast cancer cells were seeded in 6 cm dish, after 12 h, the indicated plasmids transfection was conducted with Lipofectamine 2000 (Thermo) following the manufacturer’s instruction. Briefly, serum-free medium (150 µL) with appropriate amounts of DNA and liposomes respectively were added into two 1.5 ml centrifuge tubes, then the two tubes were mixed into one and incubated for 20 min. The transfection mixture was added to the cells, then cells were placed for culture in a 37 °C CO_2_ incubator for 4–6 h, the old medium were replaced with fresh DMEM complete medium, 24 h after transfection, the cell lysates were collected and subjected to western blot.

### Chromatin immunoprecipitation (ChIP)

ChIP assays were performed with the MDA-MB231 cells using the Sp1 primary antibody to immunoprecipitate protein-DNA complexes. Briefly, the chromatin samples were precleared with protein A/G agarose/salmon sperm DNA beads for 1 h and then immunoprecipitated with antibodies against IgG and Sp1. After 12 h of incubation, the samples were incubated with protein A/G agarose beads for 2 h. After five sequential washes, cross-links were eluted with elution buffer plus proteinase K and reversed at 65 °C for 12 h. DNA was extracted with phenol-chloroform. Co-precipitated chromatin DNA was analyzed by PCR using a pair of primers that amplify the 463 to 318 bp region of *FOSL2* promoter. For each time point, 1% of the chromatin was assayed for equal loading (input). The input was conducted for each condition tested.

### Luciferase reporter assay

MDA-MB231 and 4T1 cells were transiently transfected with the pGL3 promoter luciferase reporter vector and the pGL3 promoter vector with the *FOSL2* promoter region for 12 h, then cells were treated with the indicated concentrations of ZN444B. Cells were lysed with cell culture lysis reagent (Promega) for 15 min at room temperature. Luciferase was measured using the Dual-Luciferase assay kit (Promega, Cat# E1960).

### RNA isolation analyses

RNA isolation was according to the manufacturer’s protocol. Briefly, MDA-MB231 cells were treated with or without ZN444B for 24 h, RNA was extracted via RNEasy Plus Mini Kit which eliminates genomic DNA and QIAshredder columns (Qiagen, Hilden, Germany).

### Cell death analyses

Cell death assay was performed as previously reported [29]. Briefly, breast cancer cells were treated with the indicated compounds for 24 h, then cells were stained using the Annexin V-FITC/PI apoptosis detection kit and measured using a BD Biosciences FACS Aria flow cytometer.

### Cell viability assay

Cell viability assay was performed as previously reported [30]. Briefly, breast cancer cells (5 × 10^3^ cells/well) were seeded in 96 well plates. After 24 h, cells were treated with different concentrations of ZN444B or SAHA. Cell viability was measured by MTS assay, the aqueous one solution (Promega) was used according to manufacturer instructions, and the absorption at 490 nm was measured.

### Colony formation assay

MDA-MB231 and 4T1 cells were seeded in a 6 well plate, 12 h later, cells were treated with the indicated concentrations of ZN444B. Culture medium was refreshed every other day. Cells were cultured for 1–2 weeks. Then the clones were fixed with 4% paraformaldehyde, stained with 0.1% crystal violet, and counted manually.

### Molecular docking and molecular dynamics simulation

Molecular docking study was carried out by using AutoDock 4.2.6 [31]. The crystal structure of human HDAC1 (PDB ID: 4BKX) retrieved from Protein Data Bank (http://www.pdb.org) was used for molecular docking. All missing terminal residues of protein structures were repaired by Swiss-PdbViewer v.4.10 software, then prepared by removing crystallographic waters, and adding polar hydrogen and Kollman charges for docking study [32]. The 3D structures of SAHA and ZN444B were built with Chemoffice 2019. The grid box center was set as coordinates of x, y, z= -49.617, 18.378, -4.783, and the grid size was 28.5 Å x 24.75 Å x 24.75 Å. The other parameters were set as default. The binding interaction of the protein-ligands complex has been observed by using UCSF Chimera 1.16 and BIOVIA Discovery Studio Visualizer v21.1.0.20298 (BIOVIA). The lowest energy conformation was selected for MD simulation analysis. All MD simulations were performed using the GROMACS (2020.6). The topology for the HDAC1-ZN444B complex was prepared using Amber99 and GAFF force fields respectively [33]. All molecules have been solvated into the water environment and added ions. HDAC1-ZN444B complex was performed the energy minimization to ensure no steric clashes of the system. Before the 10ns MD simulation, we performed NVT and NPT equilibration. The MD simulation of the HDAC1-ZN444B complex was performed at 10 ns with a time step of 2 fs. Finally, we analyzed the MD simulation results.

### Cell migration assay

Breast cancer cells were first starved overnight, and then cells were resuspended in 100 µl serum-free medium and added to each transwell (5 × 10^4^ cells/well), then cells were treated with ZN444B or not at 37 °C for 12 h. Migrated cells on the undersurface were fixed with 4% paraformaldehyde and stained with 0.1% crystal violet. Non-invaded cells on the upper side of the transwell were removed using cotton swabs. Images were acquired using an inverted microscope (Olympus, Tokyo, Japan) and cells were counted manually.

### Wound healing assay

Briefly, breast cancer cells were plated on 6 well plates (5 × 10^5^ cells/well), and a vertical scratch was created at the center of the well using 1 mL aseptic liquid remover, then cells were treated with ZN444B or not for 48 h. The migration of the breast cancer cells was assessed using Image-Pro Plus Analysis software (Media Cybemetics, Silver Spring, MD, USA, https://www.image pro plus 6.0).

### Three-dimensional on-top assay

Briefly, 100 µL matrigel solution per well was added into a 48-well plate and kept at 37 °C for 30 min to solidify. Then 1.5 × 10^4^ MDA-MB231 cells were re-suspended in 150 µL DMEM and seeded on solidified matrigel. After 15 min, 100 µL DMEM containing 10% matrigel and indicated concentrations of ZN444B was added on top of the plated culture. The upper matrigel-medium mixture was replaced every 2 days. Three independent experiments were carried out in triplicate.

#### In silico predictions with JASPAR Database

The usage method of JASPAR database was performed as previously reported [34]. Briefly, the transcription factor name (Sp1) was entered in the search box of JASPAR database, and database and species parameters (Homo sapiens) was set as needed. After retrieving the binding sequence of transcription factors, the ID that needs to be used for prediction was checked and click the “Scan” option, then the promoter sequence (*FOSL2*) to be analyzed was entered. The prediction results display the binding site information between transcription factors and promoter sequences, including comprehensive scores, sequence start and end positions, sense or antisense chain information, and predicted binding sequences. Lastly, the binding sequence with the highest comprehensive score was selected for further in vivo validation.

### RNA interference assay

SiRNAs targeting *HDAC1*,* FOSL2* or non-specific control were transfected into MDA-MB231 and 4T1 cells with lipofectamine 2000 reagent (ThermoFisher) according to the manufacturer’s instruction. SiRNAs were synthesized in TsingKe (Beijing, China), and 48 h after transfection, the cell lysates were collected and subjected to western blot analysis. SiRNA targeting *HDAC1* used is: 5′-TAAGGTTCTCAAACAGTCG-3′ and 5′-AAGCCGGUCAUGUCCAAAGUA-3′, siRNA targeting *FOSL2* used is: 5′-GGAUUAUCCCGGGAACUUUTT-3′ and 5′- AAAGUUCCCGGGAUAAUCCTT − 3′.

#### RT-PCR

RNA samples from breast cancer cells were prepared using Trizol (Invitrogen, Carlsbad, CA, USA) according to the manufacturer’s protocols. Total RNA (1 µg) was converted to cDNA using oligo dT primer. The relative expression of *FOSL2* was analyzed by RT-PCR with *GAPDH* as an internal control. The primer sequences used for PCR (TsingKe) for *FOSL2* were 5′-AAGTGCTGTAAGGGACGCTC-3′ and 5′-TACCCGGAATTTCTGCTGGC-3′. PCR products were separated on 1.2% agarose gel and then stained with ethidium bromide. Three independent experiments were carried out in triplicate.

#### Statistical analyses

All quantitative data are presented as the mean ± SD or SEM of at least three independent experiments. Significant differences between groups were assessed using Student’s *t*-test. Data with a *p* value of less than 0.05 were considered significant.

## Results

### ZN444B is a novel coumarin-derived HDACI with potential anti-breast cancer activity

**Fig. 1:**
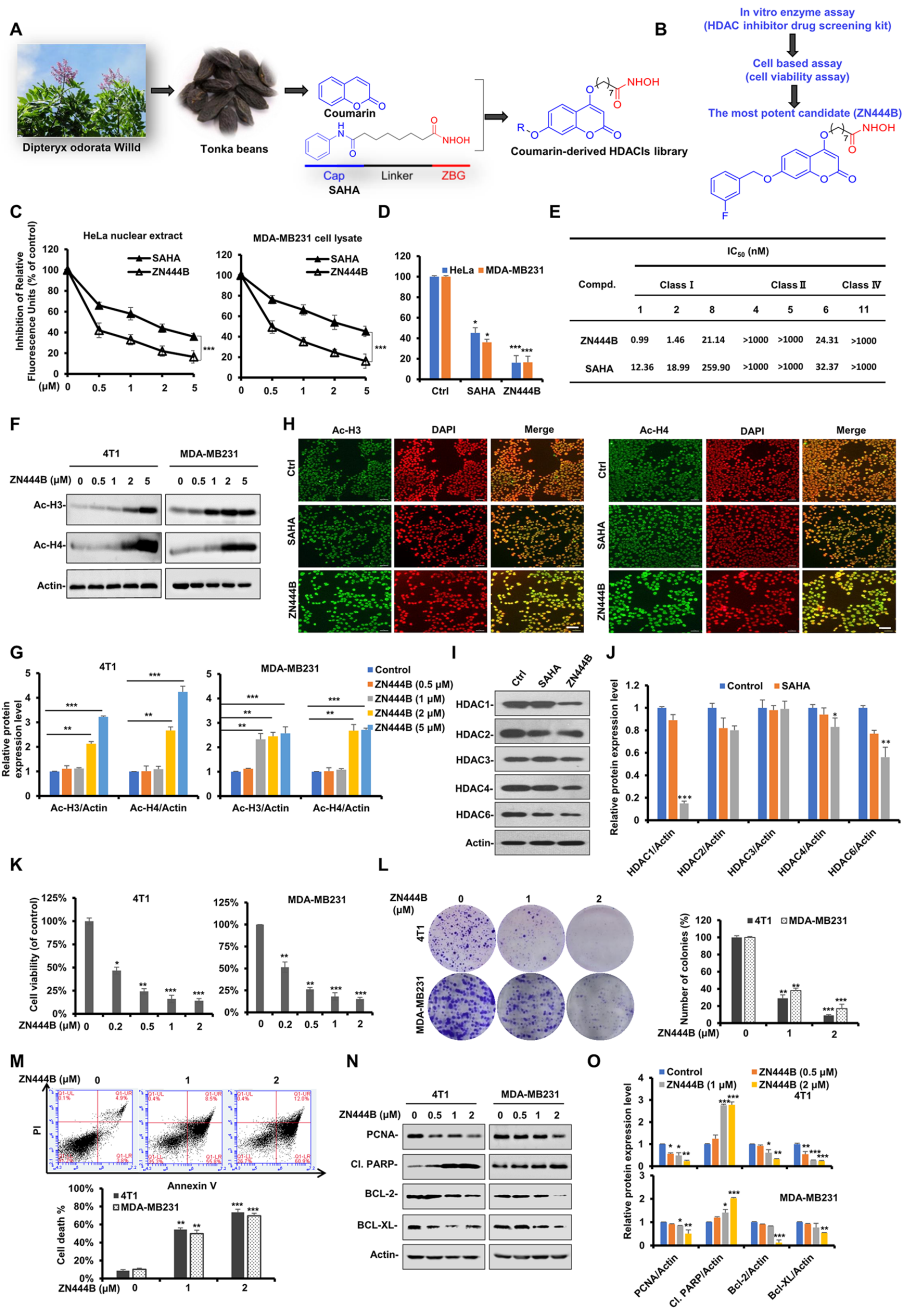
ZN444B is synthesized and identified as a novel coumarin-derived HDACI with potential anti-breast cancer activity. **A**, ** B** The design proposal of novel coumarins containing a hydroxamate-HDACIs moiety and the chemical structure of the new HDACI, ZN444B. **C**, ** D** HeLa cell nuclear extracts and MDA-MB231 cell lysates were treated with different dosages of ZN444B or SAHA, followed by co-incubation with HDAC fluorometric substrate, and HDAC activity was measured. SAHA was used as a positive control. **E** The enzyme inhibitory activity of ZN444B against HDAC1, HDAC2, HDAC4, HDAC5, HDAC6, HDAC8 and HDAC11. **F** Acetylated histone H3 (Ac-H3) and histone H4 (Ac-H4) protein level was induced by treated with indicated doses of ZN444B for 24 h in MDA-MB231 and 4T1 cell lines. Actin was used as loading control. **G** The statistical result of (**F**). All data are shown as mean ± SD, two-way ANOVA, *p < 0.05, **p < 0.01, ***p < 0.001. **H** 4T1 cells were treated with ZN444B or SAHA (2 µM) for 24 h, fixed, and stained with anti-Ac-H3 or Ac-H4 (green), and DAPI (red) and visualized by microscopy (scale bar 50 μm). **I ** MDA-MB231 cells were treated SAHA or ZN444B for 24 h and then whole cell lysates were collected. Western blot assays were performed using indicated antibodies. **J** The statistical result of (**I**). **K** 4T1 and MDA-MB231 cells were treated with ZN444B in the indicated concentration, after 48 h, the MTS assay was performed. The bars indicate the mean ± SD. **L** 4T1 and MDA-MB231 cells were seeded on 6 well, after 12 h, cells were treated with indicated concentrations of ZN444B. On day 10, number of colonies was counted in experiments repeated three times. Results represent the average of three replications. **M** 4T1 cells were treated with different dosages of ZN444B for 48 h. Cell death was assessed by Annexin V/PI staining and flow cytometry. **N** 4T1 and MDA-MB231 cells were treated with indicated concentrations of ZN444B, and the expression of PCNA, cleaved-PARP, Bcl-2 and Bcl-XL were detected by Western blot assay with indicated antibodies. **O** The statistical result of (**N**). All data are shown as mean ± SD, two-way ANOVA, *p < 0.05, **p < 0.01, ***p < 0.001

To acquire more effective therapeutic drugs for preventing breast cancer, a substituted coumarin moiety with a modification on the *p*-methoxyl group was incorporated into classic hydroxamate HDACI (SAHA) as a surface recognition group (CAP group) (Fig. 1A and Fig. S1).

Our previous study has reported coumarin-derived hydroxamate compounds, the representative compound YF349 as well as a series of more hydrophobic analogs modified on this structure [35–37]. In this study, considering that due to the different membrane permeability of compounds, the enzymatic activities and cellular activities of the compounds may not be consistent. We performed anti-breast cancer analysis of selected YF349 derivatives with HDAC1 inhibitory IC_50_ values less than 1 nM to identify their structure-activity relationship (SAR). The results in Table 1 displayed that most modified compounds exerted better enzyme and cellular inhibition rates. For the anti-proliferative activity, a general trend of increase in inhibitory activity was observed with increasing alkyl chain length of the target compounds (ZN377, ZN391A, ZN393), and the compound ZN393 displayed the best inhibition rate (29.2 ± 1.5% and 29.2 ± 1.5%). However, we can conclude that compounds with long and branched alkyl chain substitution decreased the HDAC1 inhibitory activity (ZN407, ZN469 and ZN391). When the alkyl chain was replaced by differently substituted phenyl, most compounds exhibiting reduced activities, however, ZN444B with meta-fluoro-phenyl substitution exhibits the strongest inhibitory activity against these two breast cancer cell lines (21.4 ± 1.2% and 25.4 ± 2.3%). At the same time, LogP values of compounds were carried out for prediction of the ADME properties through the molinspiration program (http://www.molinspiration.com/cgi-bin/properties). The results showed that introduction of alkyl chain and phenyl increased lipophilicity of target compounds (increase of the LogP value), and ZN444B with LogP value of 5.13 exhibited good cell permeability. The activity improvement of ZN444B may be explained by proper membrane permeability. Taken together, the appropriate linker length and the position of the substituent on the benzene ring have an impact on the activity of the compounds. Among all the compounds with inhibitory activity on both HDAC1 and two breast cancer cell lines, compound ZN444B displays the highest anti-proliferative activity, therefore, ZN444B was chosen for further biological evaluation. The chemical structure of ZN444B is shown in Fig. 1B. The synthesis scheme and characterization data of ZN444B are shown in our previous published article [36]. SAHA was used as a positive control as it is the first HDACI approved by The United States Food and Drug Administration. The HDAC inhibitory efficacy of ZN444B was stronger than that of SAHA (Fig. 1C, D). ZN444B was then tested for the enzyme inhibitory activity against HDAC1, HDAC2, HDAC4, HDAC5, HDAC6, HDAC8 and HDAC11, as shown in Fig. 1E, ZN444B exhibits a stronger binding ability with HDAC1 than other HDAC isoforms. A hallmark index of HDAC inhibition in vivo is the increased acetylation of histones H3 and H4 [38]. ZN444B significantly increased the acetylation of H3 and H4 compared with SAHA (Fig. 1F-H). In addition, we also investigated the effect of ZN444B on HDACs expression, ZN444B significantly suppressed HDAC1 expression. However, the expression of HDAC3 remained relatively constant and the expression of other HDACs (HDAC2, HDAC4, and HDAC6) displayed a slight decrease compared to the control group (Fig. 1I, J). Collectively, these results suggest that ZN444B is a novel coumarin-derived HDAC1 inhibitor with optimal anti-tumor activity for breast cancer.

**Table 1:**
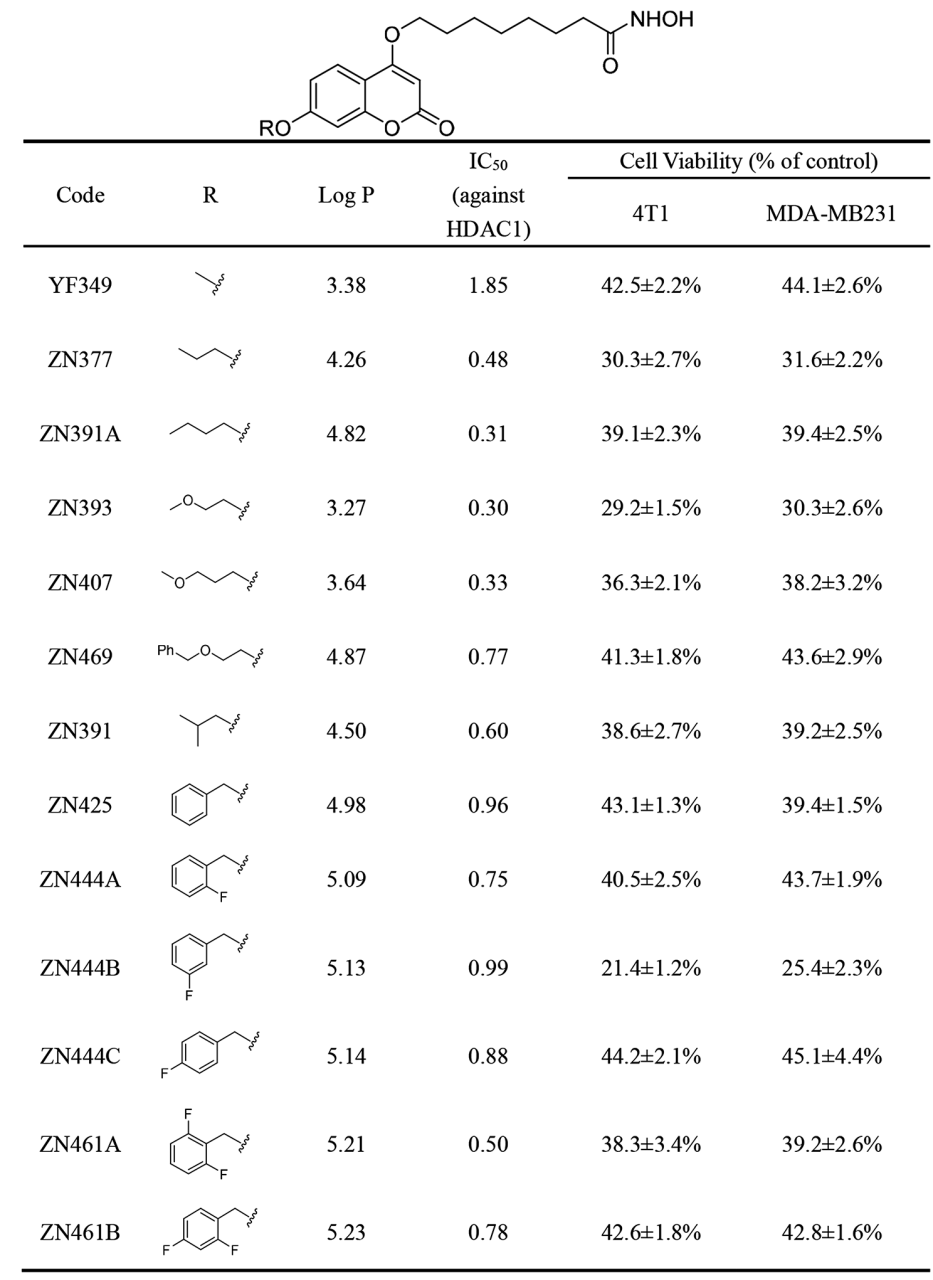
Structure–activity relationship for coumarin-derived HDACIs and inhibition rate on breast cancer cells

### ZN444B inhibits cell proliferation and colony formation and induces apoptosis in breast cancer cells

Next, we examined the effects of ZN444B on breast cancer cells. ZN444B significantly decreased cell proliferation in a dose-dependent manner (Fig. 1K and Fig. S2A) and inhibited colony formation (Fig. 1L). In addition, we also examined the responses of other breast cancer cells (MCF-7, MDA-MB436, BT20), other tumor cell lines (A549, HCT116, BGC-823) and non-tumorigenic breast epithelial cell line MCF10A with regard to ZN444B treatment, and ascertained the low cytotoxicity of ZN444B on non-breast cancer cells (Fig. S2B). Additionally, Annexin V/PI assays confirmed that ZN444B induced cell death in a dose-dependent manner (Fig. 1M). We then assessed the expression of proliferating cell nuclear antigen (PCNA) and apoptosis-related proteins with western blotting. ZN444B significantly increased the expression of cleaved-poly ADP-ribose polymerase (cleaved-PARP (Cl. PARP)) and decreased the expression of PCNA, B-cell lymphoma 2 (Bcl-2), and B-cell lymphoma extra-large (Bcl-XL) (Fig. 1N, O). ZN444B significantly inhibited cell growth and survival and induced apoptosis of breast cancer cells, indicating its promising anti-breast cancer activity.

### ZN444B inhibits migration and invasion and reverses the epithelial–mesenchymal transition (EMT) in breast cancer cells

**Fig. 2:**
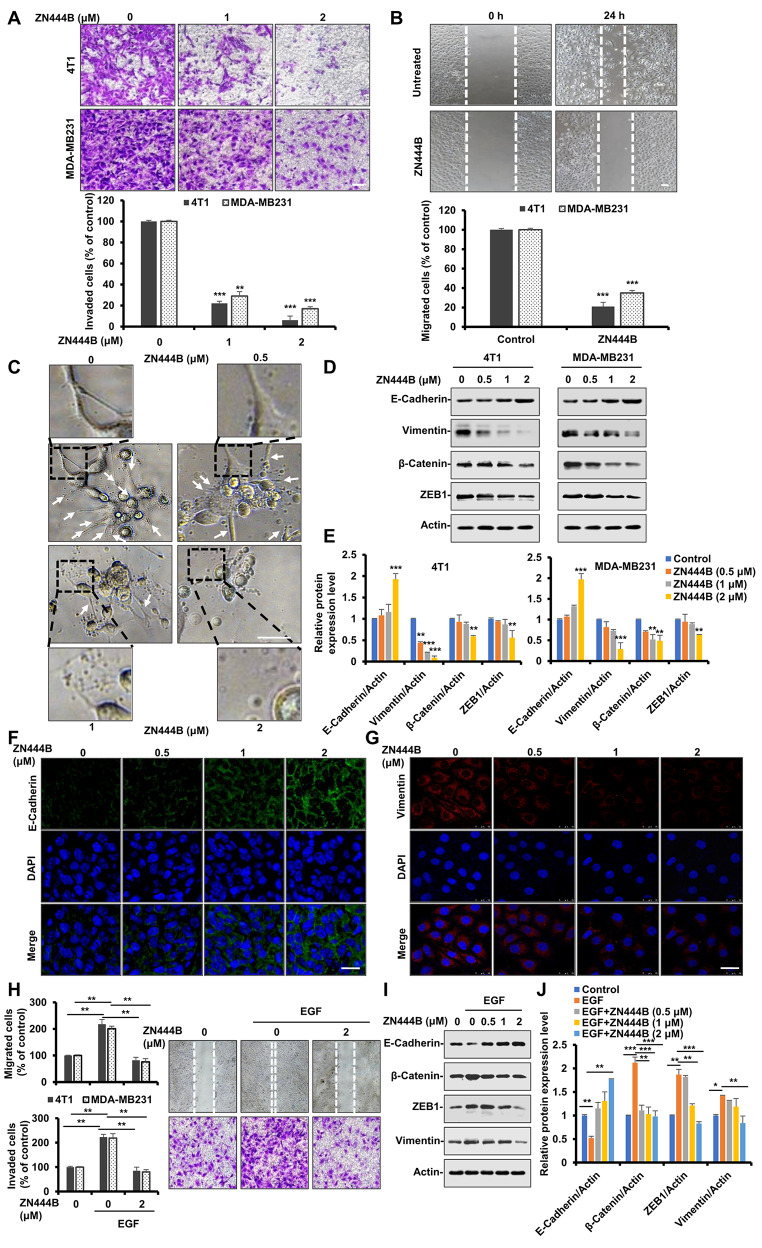
ZN444B inhibits migration, invasion and reverses EMT in breast cancer cells. **A** 4T1 and MDA-MB231 cells were treated with different concentrations of ZN444B and allowed to invade through the matrigel. Images were obtained after 12 h of incubation (upper). Invaded cell number was counted and expressed as % untreated control (lower). Data show the mean ± SD from three independent experiments. **B** MDA-MB231 cells were seeded in six-well plates. A “wound” was created after the cells grew into full confluence, then different concentrations of ZN444B were added. Images were taken after 12 h of incubation at 37 °C. **C** 3D culture assay. MDA-MB231 cells were seeded onto solidified matrigel. After 30 min, medium containing 10% Matrigel and different concentrations of ZN444B was added on top of the cells. The matrigel-medium mixture was replaced every other day. Four days later, cells were photographed. Arrows, stellate invasive structure (scale bar, 20 μm). **D** 4T1 and MDA-MB231 cells were treated with indicated concentrations of ZN444B, and the expression of EMT-related proteins were detected by Western blot assay with indicated antibodies. **E** The statistical result of (**D**). All data are shown as mean ± SD, two-way ANOVA, *p < 0.05, **p < 0.01, ***p < 0.001. **F**,** G** MDA-MB231 cells were treated with ZN444B at the indicated doses. Cells were examined for the expression of E-cadherin (green) and Vimentin (red) by immunofluorescence (IF) staining. Nuclei were stained with DAPI (blue). Scale bar, 50 μm. **H** MDA-MB231 cells in 6-well plates were scratched to create a wound and starved in serum-free medium overnight followed by exposure to EGF and ZN444B. Images were taken after 12 h of incubation at 37 °C (upper right). Cell migration was quantified manually (upper left). MDA-MB231 cells (1 × 10^5^) were resuspended in serum-free medium and seeded into the upper side of the transwell inserts precoated with Matrigel. ZN444B and EGF (50 ng/µL) were added in the bottom well. Images were obtained after 12 h of incubation (lower right). Inhibition of cell invasion by ZN444B was expressed as % untreated control. **I** Western blot analysis of MDA-MB231 cells undergoing EGF-induced EMT and ZN444B treatment (*n* = 3). In each panel, n indicates the number of independent experiments performed. **J** The statistical result of (**I**). All data are shown as mean ± SD, two-way ANOVA, *p < 0.05, **p < 0.01, ***p < 0.001

Breast cancer cells can develop invasive capability and then rapidly metastasize to other organs, many patients experience treatment failure at the metastatic stage [39]. Therefore, developing effective therapeutics for preventing breast cancer metastasis is urgently needed. For this reason, we next assessed the effects of ZN444B on breast cancer metastasis. ZN444B significantly decreased cell invasion and migration in a dose-dependent manner in different breast cancer cells (Fig. 2A, B and Fig. S2C). Three-dimensional culture systems were used to study cancer invasion and metastasis and revealed that MDA-MB231 breast cancer cells spontaneously invaded into the Matrigel and showed multiple invasive stellate structures. However, few invasive structures were found in the ZN444B-treated groups (Fig. 2C). Epithelial–mesenchymal transition (EMT) is considered to be a critical mechanism regulating the initial steps in metastatic progression [40]. We then measured the expression of EMT-related proteins with western blotting. ZN444B increased the expression of the epithelial marker E-cadherin and decreased the expression of β-catenin, vimentin, and zinc-finger E-box binding homeobox 1 (ZEB1) (Fig. 2D, E). Furthermore, an immunofluorescence assay also showed that ZN444B reversed EMT, as indicated by increased E-cadherin and decreased vimentin expression (Fig. 2F, G).

Soluble growth factors, such as TGF-β, platelet-derived growth factor, and EGF, have been found to trigger EMT [41]. Thus, we tested the effects of ZN444B on EGF-directed cell migration and invasion. ZN444B-treated cancer cells were unable to respond to the EGF stimulus to migrate and invade (Fig. 2H). Additionally, in an EGF-induced EMT model, ZN444B impaired the ability of breast cancer cells to undergo EMT (Fig. 2I, J). Taken together, these results indicate that ZN444B exerts promising inhibitory effects on breast cancer metastasis in vitro. This supports further testing the anti-metastasis efficacy of ZN444B in breast cancer in vivo.

### ZN444B inhibits breast cancer tumor growth and metastasis in vivo

**Fig. 3:**
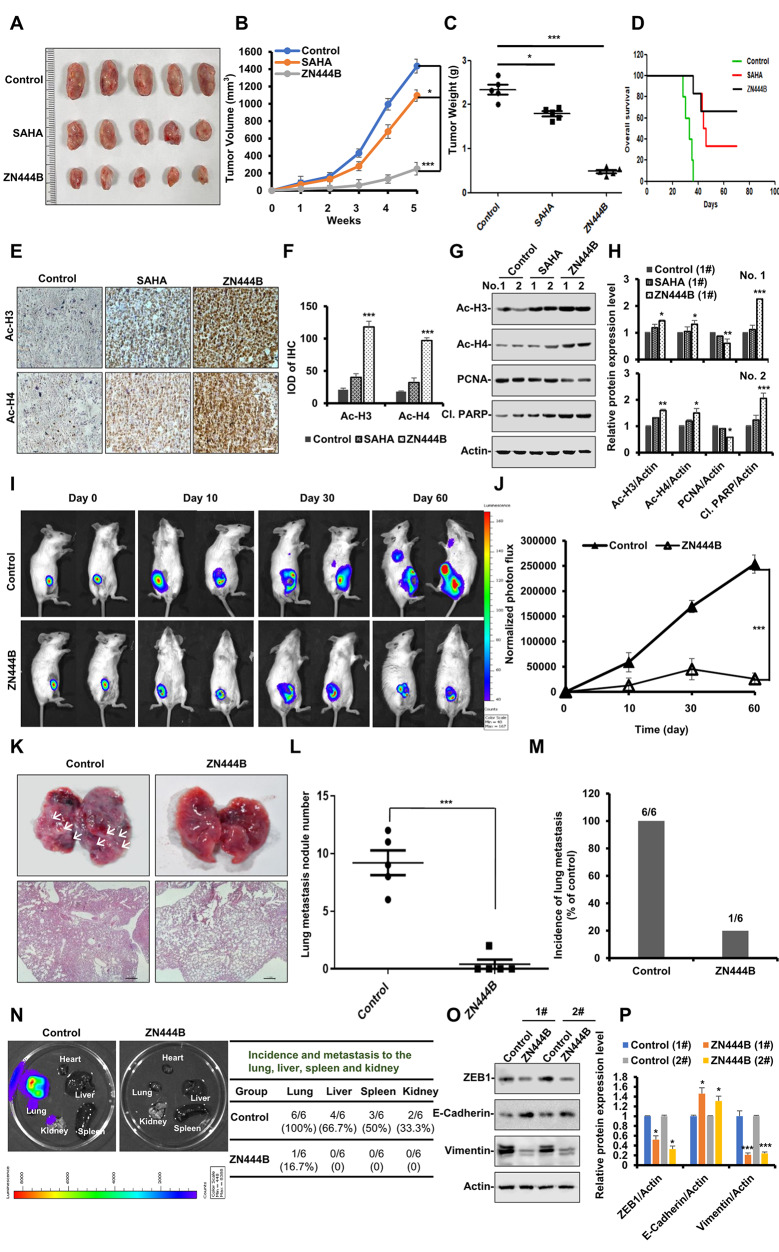
ZN444B inhibits breast tumor growth and metastasis in vivo. **A** 4T1 cells (1 × 10^5^) were injected into the mammary fat pad of female BALB/c mice, and mice were allocated to 3 groups (*n* = 5 per group), 7 days after tumor-cell implantation. After 35 days, all mice were sacrificed. Representative images of the primary tumors removed from mice after administration of ZN444B or SAHA for 28 days. **B** Primary tumor volume was measured each week (* *p* < 0.05, *** *p* < 0.001). **C** Primary tumor weight in each group was measured (* *p* < 0.05, *** *p* < 0.001). **D** Overall survival rate in the 4T1 breast tumor animal model. **E** Primary tumors were fixed and paraffin embedded. Five-micrometer (5 μm) sections were analyzed by IHC using an anti-Ac-H3 and Ac-H4 antibodies (scale bar, 100 μm). **F** The IHC results were analyzed by Image-Pro Plus 6.0 (*n* = 5 fields of view). **G** Primary tumors were lysed and applied to immunoblots using the indicated antibodies, with actin as a loading control. Acetylated histone H3/H4 protein levels and the expression of PCNA and Cleaved-PARP in primary tumor tissue were detected using western blot analysis. **H** The statistical result of (**G**). All data are shown as mean ± SD, two-way ANOVA, *p < 0.05, **p < 0.01, ***p < 0.001. **I** 4T1-Luc cells were injected into the 4th mammary fat pad of female BALB/c mice, resulting in a primary breast tumour. Mice were divided into 2 groups (*n* = 5 per group) on day 7. Real-time images on treatment day 0, day 10, day 30 and day 60 were acquired using the Xenogen IVIS. **J** Quantification of bioluminescence. Data shown are mean ± SD. *** *p* < 0.001. **K-M** Metastatic lung nodules were visualized and then counted manually, and differences were evaluated with Student’s *t* test; *** *p* < 0.001. **N** Ex vivo bioluminescence images were obtained in each group to determine the effects of ZN444B against distant metastasis. Metastasis incidence in distant organ was quantified. **O** Primary tumors were lysed and applied to immunoblotting with the indicated antibodies. Actin was used as a loading control. **P** The statistical result of (**O**). All data are shown as mean ± SD, two-way ANOVA, *p < 0.05, **p < 0.01, ***p < 0.001

To investigate the anti-breast cancer effect of ZN444B in vivo, we used an orthotopic autograft model. Treatment of mice with ZN444B significantly inhibited tumor growth and decreased tumor volume compared with the control group and SAHA treatment (Fig. 3A-C), this result was consistent with our in vitro data that ZN444B exerts stronger anti-breast cancer activity than SAHA (Fig. S3). In addition, ZN444B significantly prolonged the overall survival of mice with breast cancer compared with the control group and SAHA treatment group (Fig. 3D). The acetyl-histone H3 (Ac-H3) and acetyl-histone H4 (Ac-H4) levels in these primary tumor tissues confirmed a stronger inhibitory effect of ZN444B on histone deacetylases compared with SAHA, which confirmed the HDAC inhibitory effect of ZN444B on breast cancer in vivo (Fig. 3E, F). Furthermore, western blotting revealed that ZN444B significantly decreased the expression of PCNA and increased the expression of cleaved PARP in these primary tumor tissues (Fig. 3G, H). These results were consistent with our in vitro data observed in ZN444B-treated breast cancer cells.

We next examined the in vivo efficacy of ZN444B on breast cancer metastasis. We used an orthotopic autograft model in which 4T1 cells encoding a luciferase reporter gene were injected into the mammary fat pad of BALB/c mice and monitored by bioluminescence. ZN444B resulted in substantially decreased tumor growth compared to the control (Fig. 3I, J). Intriguingly, tumor growth was more diminished after prolonged ZN444B treatment (60 days) than after shorter treatment (30 days). These results indicate that prolonged exposure to ZN444B attenuated the incidence of breast tumor formation and ultimately inhibited tumor growth. In addition, the breast tumor began to appear in the lungs after 30 days, and after 60 days, the tumor cells grew aggressively in the mouse mammary fat pad and spontaneously metastasized to distant organs in the control group but not in the ZN444B-treated groups (Fig. 3I, J). Furthermore, in the ZN444B-treated group, metastasis nodules were hardly observed in the lungs (Fig. 3K), and the occurrence of lung metastases was significantly reduced (Fig. 3L, M).

Next, the major organs of all mice were removed and imaged for tumor presence. Only one mouse in the ZN444B group was found to bear lung metastasis, and no other distant metastases were observed (Fig. 3N). By contrast, the untreated group progressed in a more aggressive manner, and all six mice developed metastases, which were observed in the lungs (*n* = 6), liver (*n* = 4), spleen (*n* = 3), and kidneys (*n* = 2). Western blotting was performed to detect the levels of EMT-related proteins in primary tumor tissue (Fig. 3O, P). ZN444B increased the expression of the epithelial marker E-cadherin and decreased the expression of ZEB1 and vimentin. Altogether, consistent with our in vitro data observed in breast cancer cells, ZN444B significantly inhibited breast tumor growth and spontaneous metastasis in vivo.

### ZN444B significantly inhibits FOSL2 expression and suppresses the growth and metastasis of breast cancer in a FOSL2-dependent manner

**Fig. 4:**
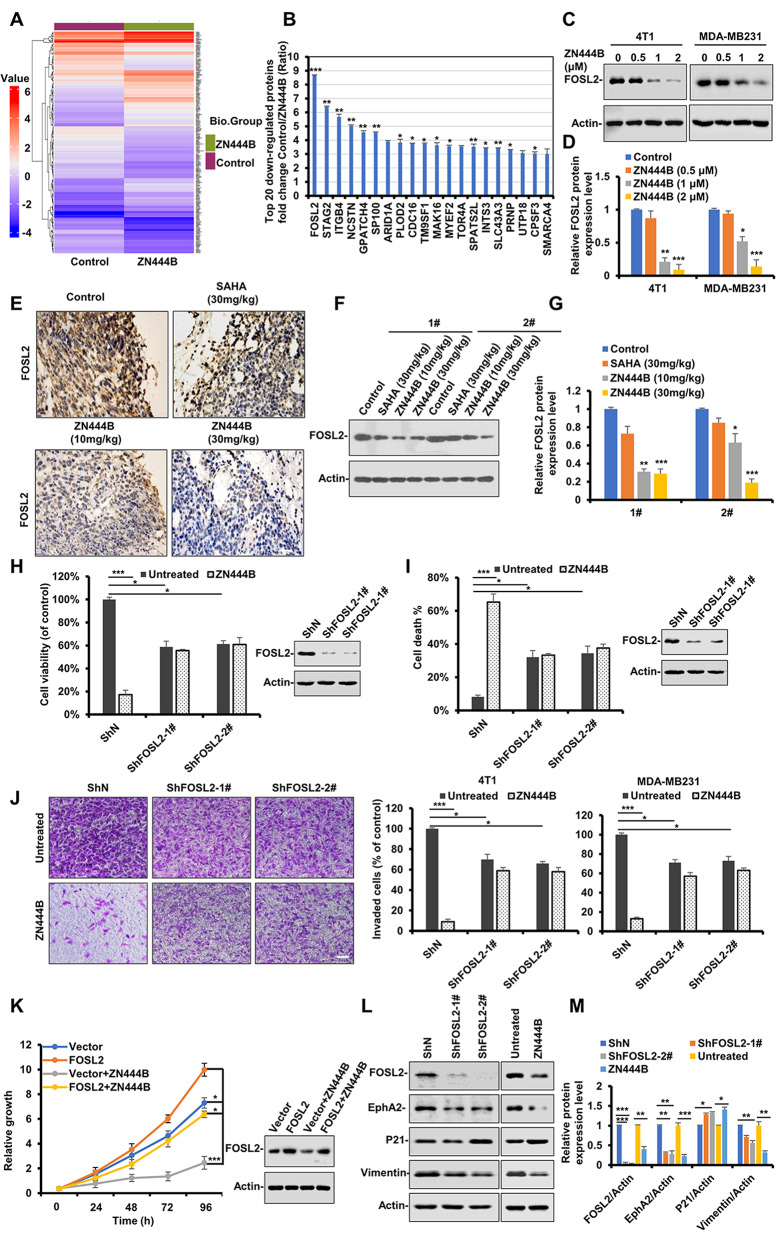
ZN444B significantly inhibits FOSL2 expression and suppresses the growth and metastasis of breast cancer in a FOSL2-dependent manner. **A** lustering heatmap of all significant proteins after MDA-MB231 cells were treated with ZN444B. **B** The fold changes of the top 20 downregulated proteins in MDA-MB231 cells treated with or without ZN444B by LC/MS-MS analysis (fold changes < 0.5; *p* < 0.05, ZN444B vs. Control). **C** MDA-MB231 and 4T1 cells were treated with the indicated concentrations of ZN444B, and the expression of FOSL2 protein was analyzed by western blot assay. Actin was used as a loading control. **D** The statistical result of (**C**). All data are shown as mean ± SD, two-way ANOVA, *p < 0.05, **p < 0.01, ***p < 0.001. **E-G** FOSL2 protein levels in 4T1 primary tumor tissue were detected using IHC staining and western blot analysis. Scale bar, 100 μm. Actin was used as a loading control. The FOSL2 protein level in primary tumor tissue was detected using western blot analysis. **H** MDA-MB231 cells with stable knockdown of FOSL2 (ShFOSL2-1#, ShFOSL2-2#) or vector control (ShN) were treated with ZN444B for 48 h or not, the MTS assay was performed. The bars indicate the mean ± SD. **I** MDA-MB231 cells with stable knockdown of FOSL2 (ShFOSL2-1#, ShFOSL2-2#) or vector control (ShN) were treated with ZN444B for 24 h or not. Cell death was assessed by Annexin V/PI staining and flow cytometry. **J** MDA-MB231 and 4T1 cells with stable knockdown of FOSL2 (ShFOSL2-1#, ShFOSL2-2#) or vector control (ShN) were treated with ZN444B for 24 h, and then cells were allowed to invade through matrigel. Images were obtained after 12 h of incubation (upper). Invaded cell number was counted and expressed as % untreated control (lower). Data show the mean ± SD from three independent experiments. **K** MDA-MB231 cells were transfected with Flag-FOSL2 plasmids for 12 h, then cells were treated with ZN444B for 24 h or not, then cell growth were detected by MTS assay, the bars indicate the mean ± SD. **L** 4T1 cells with stable knockdown of FOSL2 (ShFOSL2-1#, ShFOSL2-2#) or vector control (ShN) and 4T1 cells treated with ZN444B or not, and the expression of the indicated proteins were analyzed by western blotting. Actin was used as a loading control. **M** The statistical result of (**L**). All data are shown as mean ± SD, two-way ANOVA, *p < 0.05, **p < 0.01, ***p < 0.001

We next explored the detailed mechanisms by which ZN444B suppresses cell growth and metastasis of breast cancer via proteomic analysis using Liquid chromatography tandem mass spectrometry (LC-MS/MS) (Fig. 4A and Fig. S4A, B). Using clustering analysis and heatmaps, we observed significant differences in protein expression between ZN444B-treated and untreated MDA-MB231 cells: 120 upregulated proteins and 135 downregulated proteins. To identify novel potential therapeutic targets and clinical prognostic indicators for breast cancer, we mainly focused on the significantly downregulated proteins after ZN444B treatment. Among the top 20 significantly downregulated proteins (Fig. 4B and Fig. S4C), FOSL2 was identified to be the most significantly downregulated protein in ZN444B-treated breast cancer cells.

Former studies have reported that FOSL2 plays a pivotal role in the progression of diverse human tumors and is amplified in a variety of human tumors, including breast cancer [42, 43]. These results suggest that FOSL2 plays essential roles in the anti-tumor effects of ZN444B. A comprehensive consideration of these results led us to focus on FOSL2 for further study. We then tested the effects of ZN444B on FOSL2 expression in an in vitro cell model and 4T1 breast cancer nude mice model. in vitro, ZN444B significantly downregulated FOSL2 protein levels in 4T1 and MDA-MB231 cells in a dose-dependent manner (Fig. 4C, D). in vivo, ZN444B markedly downregulated FOSL2 protein expression in primary tumors in the 4T1 breast cancer model (Fig. 4E-G). FOSL2 knockdown significantly weakened the inhibitory effect of ZN444B on breast cancer cell growth and metastasis in vitro, resulting in breast cancer cells being insensitive to ZN444B treatment (Fig. 4H-J).

To further confirm that ZN444B inhibits breast cancer in a FOSL2-dependent manner, we transfected cells with the pcDNA3.1-FOSL2 vector to rescue ZN444B-induced FOSL2 inhibition. FOSL2 overexpression significantly abrogated the ZN444B-induced inhibition of breast cancer cell viability (Fig. 4K). A recent study reported that the FOSL2 transcription factor could drive the expression of oncogene EphA2 to induce cell proliferation, metastasis, and tumor progression in various tumors [44]. FOSL2 knockdown significantly downregulated EphA2 and vimentin expression and upregulated P21 expression (Fig. 4L, M). We found similar results when cells were exposed to increasing ZN444B concentrations. Overall, these results demonstrate that ZN444B significantly downregulated FOSL2 expression and may inhibit the proliferation and metastasis of breast cancer cells in a FOSL2-dependent manner.

### FOSL2 is closely associated with breast cancer progression

**Fig. 5:**
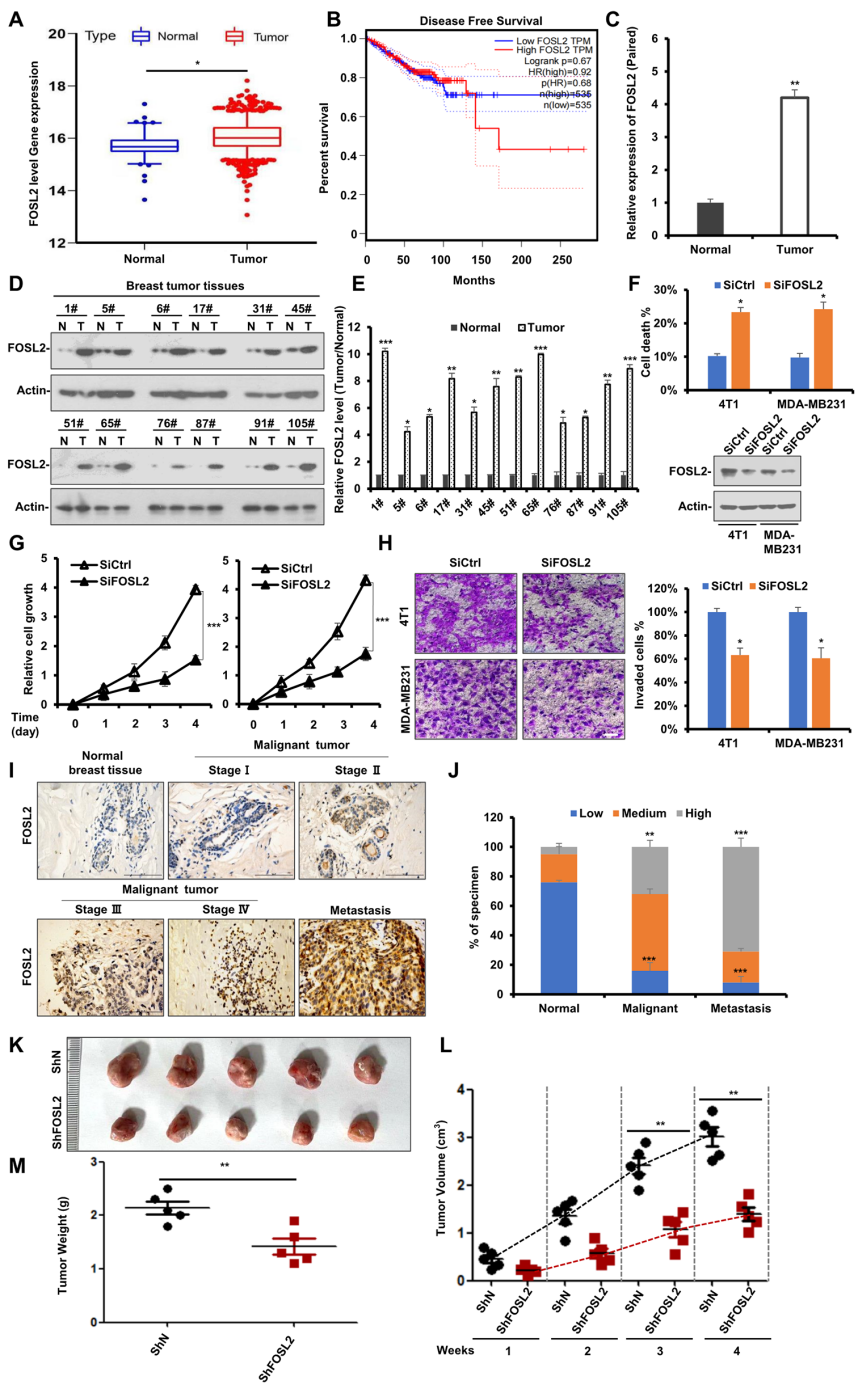
FOSL2 is closely associated with breast cancer progression. **A** FOSL2 is highly expressed in tumor by analysis of the TCGA database. **B** Kaplan-Meier survival curve of overall survival in patients with breast cancer according to FOSL2 expression. **C** FOSL2 levels were detected by RT-PCR in normal ductal tissue from normal peritumoral specimens and tumor tissues from breast reduction surgery. **D** Western blot analysis to determine the levels of FOSL2 protein in 12 paired tissues from normal peritumoral specimens and breast tumor. Actin was used as a loading control. **E** The statistical result of (**D**). All data are shown as mean ± SD, two-way ANOVA, *p < 0.05, **p < 0.01, ***p < 0.001. **F** 4T1 and MDA-MB231 cells were transfected with siRNAs targeting *FOSL2* or nonspecific control (Sictrl) for 24 h. Cell death was assessed by Annexin V/PI staining and flow cytometry. **G** 4T1 and MDA-MB231 cells were transfected with siRNAs targeting *FOSL2* or nonspecific control (Sictrl) for 24 h, the MTS assay was performed. The bars indicate the mean ± SD. **H** 4T1 and MDA-MB231 cells were transfected with siRNAs targeting *FOSL2* or nonspecific control (Sictrl) for 24 h, and then cells were allowed to invade through matrigel. Images were obtained after 12 h of incubation (upper). Invaded cell number was counted and expressed as % untreated control (lower). Data show the mean ± SD from three independent experiments. **I** Immunohistochemical (IHC) staining analysis on the expression of FOSL2 in normal ductal tissue from peri-tumor normal specimens and tissues from different tumor stages of human breast cancer, lymph node tissues were used for metastasis research. Scale bar, 100 μm. **J** The proportion of FOSL2 expression level was calculated in 3 different breast cancer stage samples (normal, malignant and metastasis). **K** 4T1 cells with stable knockdown of FOSL2 (ShFOSL2) or vector control (ShN) were injected into the mammary fat pad of female BALB/c mice (*n* = 5 per group), 7 days after tumor-cell implantation. After 35 days, all mice were sacrificed. Representative images of the primary tumors removed from mice. **L** Primary tumor volume was measured each week (** *p* < 0.01). **M** Primary tumor weight in each group was measured (** *p* < 0.01)

Previous studies have demonstrated that FOSL2 is essential for tumor growth and metastasis [45]. To further investigate the role of FOSL2 in breast cancer, The Cancer Genome Atlas database was exploited. FOSL2 was highly expressed in breast tumor tissues than in adjacent normal tissues (Fig. 5A). Furthermore, Kaplan-Meier survival curves indicated that high FOSL2 expression is closely related to the poor prognosis of breast cancer patients (Fig. 5B). Next, FOSL2 levels were determined in clinical breast cancer specimens. Compared to normal breast tissues, breast cancer tissues exhibited upregulated FOSL2 mRNA and protein levels (Fig. 5C-E). Moreover, transient FOSL2 knockdown by siRNA significantly decreased breast cancer cell growth and metastasis and increased the percentage of cell death (Fig. 5F-H). Furthermore, immunohistochemical analysis was conducted on tissues at different tumor stages of human breast cancer. FOSL2 expression was increased in breast cancer, and FOSL2 was more highly expressed in patients with advanced and metastatic malignancies (Fig. 5I, J and Table S1).

To further explore the effects of FOSL2 on tumorigenesis in vivo, FOSL2 stable knockdown MDA-MB231 and control cells were injected into the flanks of nude mice. The diameters of the tumors were measured every 7 days from the 10th day after cell transplantation. Compared with tumors formed by injection of control cells, tumors formed by FOSL2 knockdown cells were much smaller in size and weight (Fig. 5K-M). Collectively, these results demonstrate that FOSL2 expression levels are positively correlated with breast tumor progression and that targeting FOSL2 may be a promising strategy for advanced breast cancer treatment.

### Molecular docking and molecular dynamics simulation

**Fig. 6:**
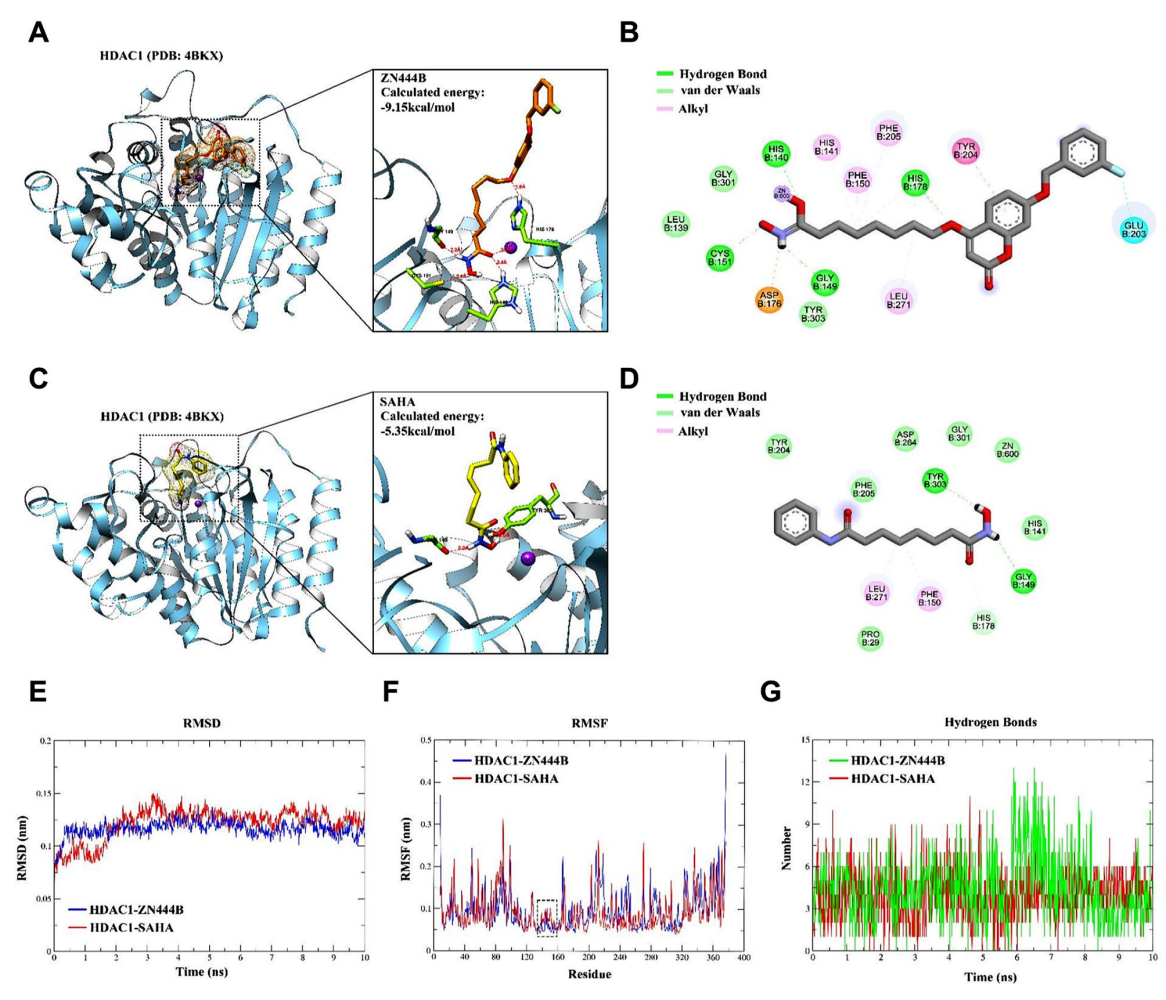
Molecular binding model and molecular dynamic simulation of ZN444B and SAHA with HDAC1. **A** The 3D binding mode of ZN444B (orange) in the active site of HDAC1. The protein and ligand HDAC1 and ZN444B are shown by cartoon and stick respectively with key residues labeled and demonstrated as green sticks, and the hydrogen bonds are labeled by red dashed lines. **B** Diagrammatic illustration of the interaction between HDAC1 binding site residues and ZN444B by BIOVIA Discovery Studio Visualizer software. Ligand is presented by gray stick, green dashed line is conventional hydrogen bonds, light green dashed line is Van der Waals, deep pink dashed line is Pi-Pi stacked, and light pink dashed line is Alkyl. **C** The 3D binding mode of SAHA (yellow) in the active site of HDAC1. The protein and ligand HDAC1 and SAHA are shown by cartoon and stick respectively with key residues labeled and demonstrated as green sticks, and the hydrogen bonds are labeled by red dashed lines. **D** 2D binding mode of SAHA into the HDAC1. Ligand is presented by gray stick, the green dashed line is conventional hydrogen bonds, the light green dashed line is Van der Waals, and the pink dashed line is Pi-Alkyl. **E** Backbone Root-mean-square deviations (RMSD) of HDAC1 during the 10ns MD simulation, the ZN444B-HDAC1 complex is presented by the blue line, and the SAHA-HDAC1 complex is presented by the red line. **F** Root mean square fluctuation (RMSF) values of HDAC1 in complex with ZN444B (green) and in complex with SAHA (red) over 10 ns. **G** The number of hydrogen bonds formed between the protein and ligands during the structural rearrangement

To better elucidate the effective HDAC inhibitory activity of ZN444B, molecular docking was performed to explore the binding mode of ZN444B with HDAC1 (PDB ID: 4BKX) [46–48]. As illustrated in (Fig. 6A, C), ZN444B and SAHA shared similar combination patterns with HDAC1. However, compared with positive drug SAHA, ZN444B exhibited stronger binding affinity to HDAC1  with a lower docking score of -9.15, while the binding affinity of SAHA was moderate with a docking score of -5.35. The cap group accommodated at the surface while making hydrogen bond interactions with ASP178 and GLU 203. A π-π stacking was observed between the phenyl of ZN444B and TYR204 sitting in the surface region of HDAC1, which contributed to enhancing the stability of the binding model between ZN444B and HDAC1 (Fig. 6B). The hydroxamic acid group of ZN444B effectively interacted with GLY149, HIS140, CYS151, and bound to the zinc^2+^ ion in a chelating manner, which contributed to the stronger binding capacity of ZN444B (Fig. 6B, D).

To further validate the stability of the binding pose mentioned above, we conducted molecular dynamics simulation to elucidate the potential mechanism underlying the interaction between ZN444B, SAHA and HDAC1. To this end, we determined the binding stability between receptor and ligand using short durational molecular dynamic simulation (10 ns) of HDAC1-ZN444B and HDAC1-SAHA, which were obtained by docking result. Prior to the start of the simulation, we prepared the topology file of HDAC1 using Amber99 force field, meanwhile, topology files of ligands were prepared by GAFF force field. The Receptor-ligand complex was conducted energy minimization and achieved NVT and NPT equilibrium state in advance.

Root-Mean-Square Deviation: Here, we carried out root-mean-square deviation (RMSD) analysis of the backbone atoms of HDAC1 and heavy atoms of ZN444B and SAHA for monitoring the complex dynamic stabilities. As given in Revised Fig. 6E, RMSD profile indicated that the HDAC1-SAHA system achieved an equilibrium state at 2.2 ns and the average RMSD among 2.2–10 ns simulation is approximately 0.13 nm. Compared with HDAC1-SAHA system, the HDAC1-ZN444B system exhibited enhanced conformational stability and achieved equilibrium at about 0.4 ns. The average RMSD between 0.4 and 10 ns was approximately 0.11 nm. Therefore, ligand ZN444B binds to HDAC1 and fast achieved to equilibrium with lower fluctuation of RMSD value. These results suggest that the docked ZN444B molecule may stabilize the HDAC1 conformation more effectively than SAHA.

Root-Mean-Square Fluctuation: To identify the critical residues of protein interacting with ligand, we computed the Root Mean Square Fluctuation (RMSF) of HDAC1-ZN444B and HDAC1-SAHA. As given in Revised Fig. 6F, there were several critical residues identified as high association with protein-ligand interaction, Trp135-Ile160 amino acid regions (labeled by black box). In this region, the ZN444B-HDAC1 system displayed lower RMSF values compared to the SAHA-HDAC1, which means the active site was stabilized by ZN444B. In summary, molecular dynamics simulation results clearly showed that ZN444B effectively stabilizes the protein conformation compared to SAHA.

Hydrogen bond: Analysis of the hydrogen bond count between two ligands and HDAC1 reveals that compared to SAHA, ZN444B forms a significantly higher number of hydrogen bonds with the binding pocket during the 6-7ns simulation process, displayed higher binding efficiency to HDAC1 (Fig. 6G).

These above results indicated that ZN444B had a high affinity close to the active site pocket of HDAC1 and inhibited the catalytic activity of HDAC1.

### ZN444B decreases FOSL2 expression by inhibiting the deacetylase activity of HDAC1 on Sp1 and disrupting Sp1 binding to the *FOSL2 *promoter in breast cancer cells

**Fig. 7:**
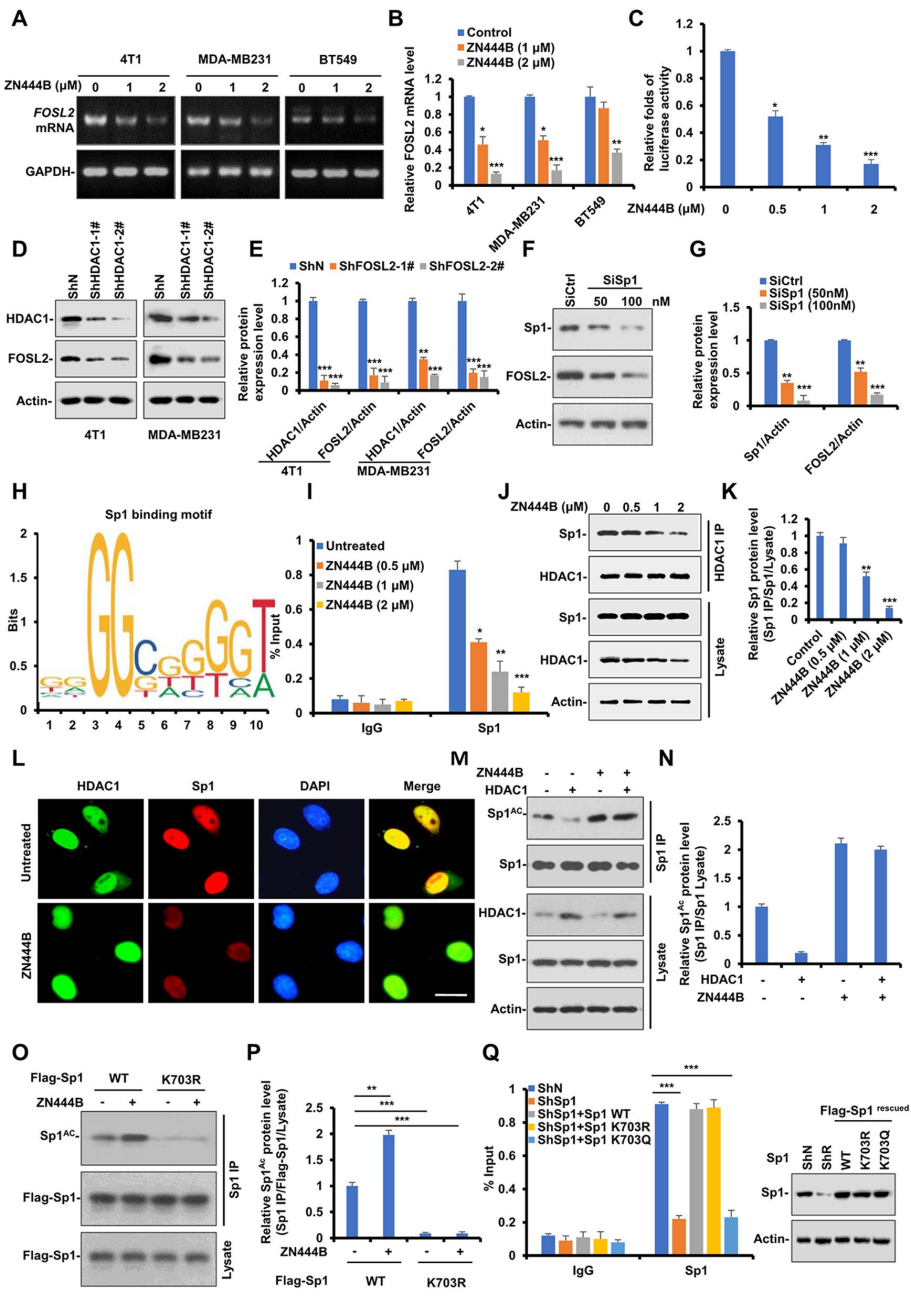
ZN444B decreases FOSL2 expression through inhibiting the deacetylase activity of HDAC1 on Sp1 and disrupting Sp1 binding to the *FOSL2 *promoter in breast cancer cells. **A** A panel of breast cancer cells (MDA-MB231, 4T1 and BT549) were treated with ZN444B with the indicated concentrations for 24 h and then analyzed for FOSL2 mRNA expression by RT-PCR. **B** The statistical result of (**A**). All data are shown as mean ± SD, two-way ANOVA, *p < 0.05, **p < 0.01, ***p < 0.001. **C** Human FOSL2 promoter luciferase reporter was transfected in 4T1 cells, 12 h later, cells were treated with increased concentrations of ZN444B for 24 h, then relative luciferase activity was analyzed. Data represent mean ± S.D., ** *p* < 0.01. **D** 4T1 and MDA-MB231 cells were transfected with siRNAs targeting HDAC1 (Si-HDAC1 1# and 2#) or non-specific control (Si-Ctrl). Western blot analysis was performed to detect the level of indicated proteins. **E** The statistical result of (**D**). All data are shown as mean ± SD, two-way ANOVA, *p < 0.05, **p < 0.01, ***p < 0.001. **F** 4T1 cells were transfected with siRNAs targeting Sp1 (50 and 100 nM) or non-specific control (Si-Ctrl). Western blot analysis was performed to detect the level of indicated proteins. **G** The statistical result of (**F**). All data are shown as mean ± SD, two-way ANOVA, *p < 0.05, **p < 0.01, ***p < 0.001. **H** Schematic diagram of canonical Sp1-binding motif (JASPAR Database) and potential FOSL2 responsive elements in the FOSL2 promoter. **I** 4T1 cells were treated with increased concentrations of ZN444B for 24 h, ChIP assays were conducted to detect the binding capability of Sp1 to the promoter of FOSL2. **J** MDA-MB231 cells were treated with increased concentrations of ZN444B for 24 h, then cells were lysed and immunoprecipitated using HDAC1 antibody followed by anti-Sp1 western blot. To ensure equal expression of HDAC1, more cell lysates were added in the groups treated with increased concentrations of ZN444B. **K** The statistical result of (**J**). All data are shown as mean ± SD, two-way ANOVA, *p < 0.05, **p < 0.01, ***p < 0.001. **L** 4T1 cells were treated with ZN444B for 24 h, the colocalization of HDAC1 and Sp1 were immunostained for Sp1 (red) and HDAC1 (green), and then visualized by fluorescence microscopy (scale bar 20 μm). **M** 4T1 cells were transfected with HDAC1 or not for 24 h, then cells were treated with ZN444B for 24 h or not, then cells were lysed and immunoprecipitated using Sp1 antibody followed by anti-acetylated Sp1 western blot. **N** The statistical result of (**M**). All data are shown as mean ± SD, two-way ANOVA, *p < 0.05, **p < 0.01, ***p < 0.001. **O** MDA-MB231 cells were transfected with Flag-Sp1 WT, K703R, 12 h later, cells were treated with different concentrations of ZN444B for 24 h and then whole cell lysates were collected. Western blot assays were performed using indicated antibodies. **P** The statistical result of (**O**). All data are shown as mean ± SD, two-way ANOVA, *p < 0.05, **p < 0.01, ***p < 0.001. **Q** ChIP assays were conducted in MDA-MB231 Sp1-knockdown (ShSp1) cells rescued with Sp1-WT, -K703R, or -K703Q to detect the binding capability of Sp1 to the promoter of FOSL2 (** *p* < 0.01)

Because FOSL2 protein expression was so markedly decreased by ZN444B in vitro and in vivo (Fig. 4C-G), we queried whether ZN444B treatment could attenuate FOSL2 at the mRNA level. A panel of breast cancer cells were treated with increasing ZN444B concentrations, and conventional RT-PCR revealed the suppression of *FOSL2* mRNA (Fig. 7A, B). Next, the cells were transiently transfected with the *FOSL2* promoter–luciferase construct and treated with or without ZN444B. ZN444B inhibited the promoter activity of FOSL2 in a dose-dependent manner (Fig. 7C). These data suggest that the decreased FOSL2 expression was due to the transcriptional inhibition by ZN444B.

As a novel HDACI, ZN444B could interact with the active site of HDAC1 (Fig. 6A, B) and exhibit a stronger binding ability with HDAC1 than other HDAC isoforms (Fig. 1E). Therefore, we hypothesized that HDAC1 affects FOSL2 expression. After transfection with siRNAs targeting HDAC1, FOSL2 expression was remarkably decreased (Fig. 7D, E). This suggests that HDAC1 plays a key role in sustaining FOSL2 expression.

We then sought to clarify the detailed mechanism of ZN444B-induced downregulation of *FOSL2* mRNA via inhibiting HDAC1. The transcription factor Sp1 can regulate the expression of multiple genes [49], and thus, we investigated whether Sp1 could modulate FOSL2 expression. As expected, depletion of Sp1 with siRNA resulted in FOSL2 protein downregulation (Fig. 7F, G). This indicates that Sp1 may be a transcription factor of FOSL2. Next, we determined the Sp1-binding site with *in silico* predictions using the JASPAR database [34]. Sequence analysis revealed that “ccccgccccgc” at the *FOSL2* promoter is a putative Sp1-binding site (Fig. 7H). To further confirm this result, we performed quantitative ChIP assay. Sp1 was enriched at the promoter region covering the binding site, and ZN444B inhibited its binding to the *FOSL2* promoter (Fig. 7I). Former studies have proven that the aberrant recruitment of HDAC1 to promoters through interaction with Sp1 could regulate gene transcription [50]. As shown in Fig. 7J-K, after normalization for the amount of protein present, ZN444B significantly blocked the interaction between HDAC1 and Sp1. In addition, immunofluorescence assay showed that ZN444B inhibited the association of HDAC1 with Sp1 in breast cancer cells (Fig. 7L).

We then sought to clarify the detailed mechanism of ZN444B-induced FOSL2 mRNA downregulation via abrogating the HDAC1-Sp1 complex. A former study reported that post-translational modifications such as acetylation influence the transcriptional activity of Sp1 [51]. Sp1 acetylation was significantly increased by ZN444B and decreased in HDAC1-overexpressing cells (Fig. 7M, N). Previous studies reported that Sp1 is constitutively acetylated at lysine 703 (K703) and that the acetylation at K703 plays an essential role in regulating the transcriptional activity of Sp1 [52]. Site-directed mutagenesis (K-to-R) analysis shows that Sp1 acetylation could not be detected after ZN444B treatment (Fig. 7O, P). These results demonstrate that ZN444B induced Sp1 acetylation at K703 via inhibiting HDAC1.

Next, we tested whether Sp1 acetylation could regulate its transcriptional activity on FOSL2. We generated stable Sp1-knockdown and Flag-Sp1 (including WT, acetylation-deficient K703R, and acetylation-mimicking K703Q) rescued cell lines. Recomplementation of K703Q led to markedly decreased transcriptional activity of Sp1 on FOSL2 compared with recomplementation of Sp1-WT or -K703R (Fig. 7Q), suggesting that the increased acetylation of Sp1 at K703 abrogated its binding ability to the *FOSL2* promoter. Collectively, these results suggest that ZN444B blocked the interaction between HDAC1 and Sp1, induced the acetylation of Sp1 at K703 by inhibiting the deacetylase activity of HDAC1, and abrogated the binding ability of Sp1 to the *FOSL2* promoter, finally contributing to decreased FOSL2 expression.

### ZN444B shows no toxicity in mice

We then investigated the potential toxicity of ZN444B. For this, female BALB/c mice were administered PBS or ZN444B (30 mg/kg) for 28 days, and body weight was measured once a week. No significant changes in mice body weight or major organ weight were observed (Fig. S5A, B). Histological analyses revealed no obvious damage to any major organs (i.e., the heart, liver, spleen, lungs, or kidneys) (Fig. S55C). In addition, levels of alanine aminotransferase (ALT) and aspartate aminotransferase (AST) are representative indicators of liver function, and blood urea nitrogen (BUN) is an indicator of kidney and liver conditions. Thus, we examined their levels in ZN444B-treated mice (Fig. S5D). However, ZN444B did not ALT, AST, or BUN levels. Cytochrome P450 enzymes are membrane-bound hemoproteins that play a pivotal role in the detoxification of xenobiotics, cellular metabolism, and homeostasis [53], as shown in Fig. S5E, no evident changes in cytochrome P450 protein levels were observed in ZN444B-treated mice. In addition, ZN444B showed no effects on the hematological parameters assessed by routine blood chemistry analysis (Table S2).

To investigate the long-term toxicity of ZN444B, female BALB/c mice were administered PBS or ZN444B (30 mg/kg) each day for 60 days. All mice were killed on day 60, and the organs and tissues were fixed and paraffin-embedded for H&E staining. ZN444B showed no obvious damage to the brain, bone marrow, thymocytes, or small-intestine epithelium (Fig. S5F). Collectively, these results implied that ZN444B showed superior pharmacodynamic properties and few side effects on mice in vivo at therapeutic concentrations, providing evidence that ZN444B could act as an effective anti-tumor candidate for breast cancer therapy.

## Discussion

Breast cancer is a serious health problem and the leading cause of cancer-related death among women. Many potential molecular targets inhibitors have been assessed as single agents or combined with conventional therapies in clinical trials for breast cancer. However, they eventually experience treatment failure due to severe side effects and chemotherapy resistance [54–56]. Natural compounds have demonstrated various pharmacological activities and been identified as a paramount source for novel drugs. Coumarin is a natural compound that is widely distributed in the natural kingdom. Previous studies have confirmed that natural coumarins exhibit extensive anti-tumor activity in vivo with low toxicity.

Many studies have suggested the involvement of HDACs in cancer initiation and progression and HDACIs have shown promising anti-cancer activities, e.g., the inhibition of tumor cell proliferation, survival, and metastasis [57]. To date, several HDACIs have shown excellent anti-cancer effects when combined with conventional therapies in clinical trials for breast cancer. However, most current HDACIs have shown limited effectiveness in solid tumors when used as a single agent [15]. Therefore, it is necessary to develop novel HDACIs with improved activity, investigate their exact mechanisms, and to explore new molecular therapeutic targets for breast cancer. In this study, we incorporated a substituted coumarin moiety into classic hydroxamate HDACIs and synthesized and identified a novel coumarin-hydroxamate hybrid, ZN444B, which showed promising anti-breast tumor activity both in vitro and in vivo.

We have previously developed another coumarin-derived HDACI, YF349, which exhibits excellent anti-tumor effects [37]. Nevertheless, functional studies have revealed that ZN444B inhibits breast cancer metastasis more effectively than YF349 under the same dose treatment (Fig. S6A). Docking simulation showed that the 3-fluorobenzyl of ZN444B exactly filled in the hydrophobic cavity of the enzyme surface, which could preliminarily rationalize the better inhibitory activity of ZN444B compared to YF349 (Fig. S6B). The fluorine atom is characterized by small atomic volume and high negative charge. The introduction of the fluorine atom redistributes the electronic arrangement of ZN444B and forms intermolecular hydrogen bonds with a benzene ring. This can optimize the three-dimensional conformation of molecules, making them easier to combine with HDAC1 and thus increasing the drug’s bioavailability and enhancing the drug’s effect. Furthermore, the modification of methoxy of YF349 to 3-fluorophenyl of ZN444B may have improved the lipid solubility of the compound, which could enhance the permeability of cell membranes to ZN444B. Based on the above results, we believe that ZN444B certainly warrants further investigation. A former study reported that FOSL2 is linked to ovarian cancer (OC) and inhibition of FOSL2 promotes the apoptosis of OC cells by mediating the formation of an inflammasome [58]. Another study unveiled a critical role of FOSL2 in promoting metastasis in colon cancer [59]. In addition, FOSL2 could regulate the epithelial-mesenchymal transitions (EMT), invasion, and migration by transcriptional regulation of SNAI2 in non-small cell lung cancer [60]. These results indicated that FOSL2 may be important in conferring the oncogenic potential and tumor progression. As explored in this study, FOSL2 is upregulated in breast cancer and a high FOSL2 level is associated with breast cancer development, and the protein level of FOSL2 was also remarkably downregulated under ZN444B treatment in breast cancer cells and primary tumors from 4T1 breast cancer model (Figs. 4C-G and 5). In addition. silencing FOSL2 expression decreased the sensitivity of breast cancer cells to ZN444B treatment (Fig. 4H-G). Therefore, the ability of ZN444B to block breast cancer growth and metastasis may be mainly achieved by suppressing the aberrant FOSL2 expression in breast cancer cells.

In our study, we found that FOSL2 expression positively correlates with breast cancer progression and metastasis (Fig. 5I, J). Additionally, FOSL2 downregulation remarkably decreased tumor volume in the 4T1 animal model and cell growth and metastasis in 4T1 and MDA-MB231 cells (Fig. 5K–M and F–H). Moreover, ZN444B can reduce both FOSL2 mRNA and protein levels in a concentration-dependent manner (Figs. 4C, D and 7A, B). These results demonstrate that FOSL2 plays an essential role in tumor progression and also support our conclusion that ZN444B exerts its anti-breast cancer effect via downregulating FOSL2 expression. At present, the precise regulation of FOSL2 and the detailed mechanisms underlying its inhibition of breast cancer have not yet elucidated. The results of this study revealed that the effects of ZN444B on breast cancer are in a FOSL2-dependent manner and warrant further investigation on the clinical association of FOSL2 during breast cancer development.

Several additional biological implications emerge from our present work. The novel coumarin-derived HDACI, ZN444B, was first identified as an inhibitor of breast cancer with no obvious side effects (at the treatment dose (30 mg/kg)). This study also explored the detailed mechanism of the anti-breast cancer effects of ZN444B and provided the first evidence that FOSL2 may play a key role in breast tumors. We found that ZN444B significantly inhibited FOSL2 expression, resulting in breast cancer growth and metastasis regression.

Coumarin, as a potential drug candidate for anti-tumor therapies, can cause alterations of the expression of some genes or proteins within cells. However, in this study, coumarin was inserted into the cap region of the classic HDACI, thereby strengthening the affinity between the compound ZN444B and HDAC1, which enhanced the inhibitory activity against HDAC1, ultimately downregulating the expression of FOSL2. According to the results of proteomic analysis, there are also other significantly altered proteins in response to ZN444B treatment. However, among the top 20 downregulated proteins listed in Fig. 4B, there are no reported targets for coumarin. In addition, in this study, we found that the expressions of FOSL2 and HDAC1 were not changed apparently by coumarin moiety treatment (Fig. S7), this result suggested that alterations of these proteins may mainly achieved by the enhanced HDACs inhibitory activity of ZN444B.

**Fig. 8:**
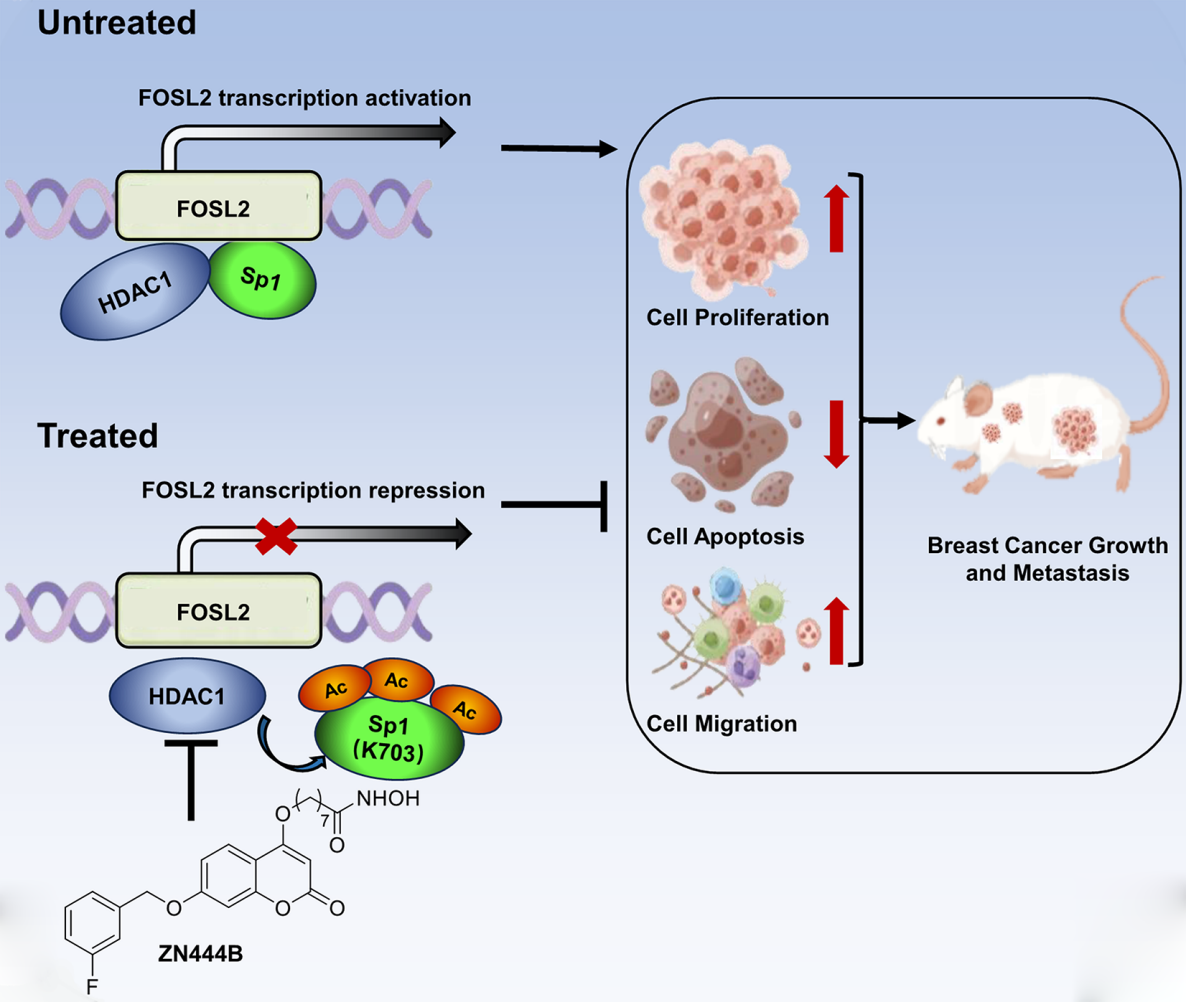
A schematic map of the role of ZN444B in breast cancer. A novel coumarin-derived HDACI, termed ZN444B, was identified in our new synthesized small molecule library. Further cell and animal experiments confirmed the promising anti-breast cancer activity of ZN444B both in vitro and in vivo. Molecular docking model shows that ZN444B binds to HDAC1 with a high affinity. Mechanistically, ZN444B inhibits the deacetylase activity of HDAC1 on Sp1, which induces the acetylation of Sp1 at K703 and abrogates the binding ability of Sp1 to the *FOSL2* promoter region, subsequently decreases the expression of FOSL2, and the low level of FOSL2 thus eventually impeding the growth and metastasis of breast cancer, eventually inhibits breast cancer development

In this study, we demonstrated that ZN444B exerts its inhibitory effect on breast cancer by regulating the HDAC1-Sp1-FOSL2 axis mainly through the following ways (Fig. 8): (1) ZN444B binds to the deacetylase domain of HDAC1, blocks the interaction of HDAC1 and Sp1, and inhibits the deacetylase activity of HDAC1 on Sp1, subsequently increases the acetylation of Sp1 at K703. Our results showed that the interaction between Sp1 and HDAC1 was disrupted, and thus, the acetylation level of Sp1 was increased after ZN444B treatment. (2) The increased acetylation at K703 disrupts the transcriptional potency of Sp1, reduces the binding of Sp1 to the *FOSL2* promoter, causes the transcriptional suppression of FOSL2, alters the activity of downstream FOSL2 targets, and finally inhibits breast cancer cell growth and metastasis. In addition, we found that genetic or pharmacologic inhibition of FOSL2 markedly decreases the level of its downstream target EphA2 (Fig. 4L, M), which is consistent with the previous research [44]. Moreover, ZN444B treatment induces P21 expression and decreases vimentin expression, implying that FOSL2 is involved in tumor progression and metastasis. Together, our findings suggest that targeting the HDAC1-Sp1-FOSL2 signal axis by ZN444B may be a promising therapeutic strategy for breast cancer.

In summary, our study synthesized and identified a novel coumarin-hydroxamate hybrid, ZN444B, with improved activity and provided mechanistic insights into the relationship between ZN444B and the HDAC1-Sp1-FOSL2 signal axis. Our findings demonstrated that ZN444B inhibits breast cancer cell growth and metastasis by specifically suppressing FOSL2 expression. In addition, ZN444B (at a therapeutic dose) shows no systemic toxicity in mice. The results of the present work lay a solid foundation for the clinical association of FOSL2 and breast cancer progression. Our findings support FOSL2 as a promising drug target for breast cancer and ZN444B as a potential candidate for the treatment of advanced breast cancer.

### Electronic supplementary material

Below is the link to the electronic supplementary material.

Supplementary Material 1

Supplementary Material 2

## Data Availability

No datasets were generated or analysed during the current study.

## Abbreviations

IHC: Immunohistochemistry
IF: Immunofuorescence
IP: Immunoprecipitation
ChIP: Chromatin immunoprecipitation
PBS: Phosphatebufered saline
PVDF: Polyvinylidene difuoride
SDSPAGE: Sodium dodecyl sulfate polyacrylamide gel electrophoresis
siRNA: Small interfering RNA
ECM: Extracellular matrix
EMT: Epithelial to mesenchymal transition
FBS: Fetal bovine serum
HDAC: Histone deacetylase
HDACI: Histone deacetylase inhibitor
FOSL2: Fos-related antigen-2
H&E: hematoxylin-eosin staining
MEM: Minimum essential medium
PBS: Phosphatebufered saline
PCR: Polymerase chain reaction
TGFβ: Transforming growth factorβ
SAHA: Suberoylanilide hydroxamic acid
PI: Propidine iodide
PCNA: Proliferation cell nuclear antigen
Bcl-2: B-cell lymphoma 2
Bcl-XL: B-cell lymphoma extra-large
EGF: Epidermal growth factor
Ac-H3: Acetylation-histone3
Ac-H4: acetylation-histone4
ZEB1: Zinc-finger E-box binding homeobox 1
ALT: Alanine aminotransferase
AST: Aspartate aminotransferase
BUN: Blood urea nitrogen
DAPI: 4′, 6-diamidino-2-phenylindole
DMSO: Dimethyl sulfoxide
WT: Wildtype

## References

[1] Siegel RL, Miller KD, Fuchs HE, Jemal A (2022). Cancer statistics, 2022. CA-A CANCER J Clin.

[2] Perou CM, Sorlie T, Eisen MB, van de Rijn M, Jeffrey SS, Rees CA, Pollack JR, Ross DT, Johnsen H, Akslen LA (2000). Molecular portraits of human breast tumours. Nature.

[3] Prat A, Pineda E, Adamo B, Galvan P, Fernandez A, Gaba L, Diez M, Viladot M, Arance A, Munoz M (2015). Clinical implications of the intrinsic molecular subtypes of breast cancer. Breast.

[4] Lee HJ, Seo AN, Kim EJ, Jang MH, Suh KJ, Ryu HS, Kim YJ, Kim JH, Im SA, Gong G (2014). HER2 heterogeneity affects trastuzumab responses and survival in patients with HER2-positive metastatic breast cancer. Am J Clin Pathol.

[5] Cesca MG, Vian L, Cristovao-Ferreira S, Ponde N, de Azambuja E (2020). HER2-positive advanced breast cancer treatment in 2020. Cancer Treat Rev.

[6] Tripathy D, Im SA, Colleoni M, Franke F, Bardia A, Harbeck N, Hurvitz SA, Chow L, Sohn J, Lee KS (2018). Ribociclib plus endocrine therapy for premenopausal women with hormone-receptor-positive, advanced breast cancer (MONALEESA-7): a randomised phase 3 trial. Lancet Oncol.

[7] Huang ML, Zhang J, Yan CJ, Li XH, Zhang JL, Ling R. Small molecule HDAC inhibitors: promising agents for breast cancer treatment. Bioorg Chem 2019, 91. 10.1016/j.bioorg.2019.103184 31408831

[8] Bots M, Johnstone RW (2009). Rational combinations using HDAC inhibitors. Clin Cancer Res.

[9] Byler S, Goldgar S, Heerboth S, Leary M, Housman G, Moulton K, Sarkar S (2014). Genetic and epigenetic aspects of breast Cancer progression and therapy. Anticancer Res.

[10] Tang F, Choy E, Tu C, Hornicek F, Duan Z (2017). Therapeutic applications of histone deacetylase inhibitors in sarcoma. Cancer Treat Rev.

[11] Munster PN, Marchion D, Thomas S, Egorin M, Minton S, Springett G, Lee JH, Simon G, Chiappori A, Sullivan D, Daud A (2009). Phase I trial of vorinostat and doxorubicin in solid tumours: histone deacetylase 2 expression as a predictive marker. Br J Cancer.

[12] de la Vega M, Diaz-Canton E, Alvarez RH (2012). Novel targeted agents for the treatment of advanced breast cancer. Future Med Chem.

[13] Catley L, Weisberg E, Tai YT, Atadja P, Remiszewski S, Hideshima T, Mitsiades N, Shringarpure R, LeBlanc R, Chauhan D (2003). NVP-LAQ824 is a potent novel histone deacetylase inhibitor with significant activity against multiple myeloma. Blood.

[14] Qiu TZ, Zhou L, Zhu W, Wang TS, Wang J, Shu YQ, Liu P (2013). Effects of treatment with histone deacetylase inhibitors in solid tumors: a review based on 30 clinical trials. Future Oncol.

[15] Vancurova I, Uddin MM, Zou Y, Vancura A (2018). Combination therapies targeting HDAC and IKK in solid tumors. Trends Pharmacol Sci.

[16] Gryder BE, Sodji QH, Oyelere AK (2012). Targeted cancer therapy: giving histone deacetylase inhibitors all they need to succeed. Future Med Chem.

[17] Han B, Wang M, Li J, Chen Q, Sun N, Yang X, Zhang Q. Perspectives and new aspects of histone deacetylase inhibitors in the therapy of CNS diseases. Eur J Med Chem 2023, 258. 10.1016/j.ejmech.2023.115613 37399711

[18] Wu B, Fathi S, Mortley S, Mohiuddin M, Jang YC, Oyelere AK (2020). Pyrimethamine conjugated histone deacetylase inhibitors: design, synthesis and evidence for triple negative breast cancer selective cytotoxicity. Bioorg Med Chem.

[19] Gryder BE, Rood MK, Johnson KA, Patil V, Raftery ED, Yao L-PD, Rice M, Azizi B, Doyle DF, Oyelere AK (2013). Histone deacetylase inhibitors equipped with estrogen receptor modulation activity. J Med Chem.

[20] Huang M, Zhang J, Yan C, Li X, Zhang J, Ling R (2019). Small molecule HDAC inhibitors: promising agents for breast cancer treatment. Bioorg Chem.

[21] Garg SS, Gupta J, Sharma S, Sahu D. An insight into the therapeutic applications of coumarin compounds and their mechanisms of action. Eur J Pharm Sci 2020, 152. 10.1016/j.ejps.2020.105424 32534193

[22] Song F, Huo X, Guo Z (2021). Anti-breast Cancer potential of natural and synthetic coumarin derivatives. Curr Top Med Chem.

[23] Sashidhara KV, Avula SR, Sharma K, Palnati GR, Bathula SR (2013). Discovery of coumarin-monastrol hybrid as potential antibreast tumor-specific agent. Eur J Med Chem.

[24] Bhatia R, Rawal RK (2019). Coumarin hybrids: promising scaffolds in the treatment of breast Cancer. MINI-REVIEWS Med Chem.

[25] Musa MA, Cooperwood JS, Khan MOF (2008). A review of coumarin derivatives in pharmacotherapy of breast cancer. Curr Med Chem.

[26] Cho JH, Shin JC, Cho JJ, Choi YH, Shim JH, Chae JI (2015). Esculetin (6,7-dihydroxycoumarin): a potential cancer chemopreventive agent through suppression of Sp1 in oral squamous cancer cells. Int J Oncol.

[27] Shan P, Yang F, Qi H, Hu Y, Zhu S, Sun Z, Zhang Z, Wang C, Hou C, Yu J (2021). Alteration of MDM2 by the small molecule YF438 exerts Antitumor effects in Triple-negative breast Cancer. Cancer Res.

[28] Ma Q, Xu Q, Zhao J, Zhang W, Wang Q, Fang J, Lu Z, Liu J, Ma L (2021). Coupling HDAC4 with transcriptional factor MEF2D abrogates SPRY4-mediated suppression of ERK activation and elicits hepatocellular carcinoma drug resistance. Cancer Lett.

[29] Gao Y, Zhang D, Wang F, Zhang D, Li P, Wang K. BRAF V600E protect from cell death via inhibition of the mitochondrial permeability transition in papillary and anaplastic thyroid cancers. J Cell Mol Med 2022. 10.1111/jcmm.17443 PMC9279591 35748101

[30] Yang LJ, Han DL, Zhan Q, Li XP, Shan PP, Hu YJ, Ding H, Wang Y, Zhang L, Zhang Y (2019). Blood TfR plus exosomes separated by a pH-responsive method deliver chemotherapeutics for tumor therapy. Theranostics.

[31] Morris GM, Huey R, Lindstrom W, Sanner MF, Belew RK, Goodsell DS, Olson AJ (2009). AutoDock4 and AutoDockTools4: automated docking with selective receptor flexibility. J Comput Chem.

[32] Forli S, Huey R, Pique ME, Sanner MF, Goodsell DS, Olson AJ (2016). Computational protein-ligand docking and virtual drug screening with the AutoDock suite. Nat Protoc.

[33] Wang J, Wolf RM, Caldwell JW, Kollman PA, Case DA (2004). Development and testing of a general amber force field. J Comput Chem.

[34] Fan C, Meng X, Yang W, Wang P, Chang W, Li P, Wang J (2023). Chemical labeling achieves 8-oxo-7,8-dihydroguanine mapping in the microRNA transcriptome. Chem Commun (Camb).

[35] Yang F, Zhao N, Song J, Zhu K, Jiang C-s, Shan P, Zhang H. Design, Synthesis and Biological Evaluation of Novel Coumarin-Based Hydroxamate Derivatives as Histone Deacetylase (Hdac) Inhibitors with Antitumor Activities. *MOLECULES* 2019, 24. 10.3390/molecules24142569 PMC6680717 31311163

[36] Zhao N, Yang F, Han L, Qu Y, Ge D, Zhang H. Development of coumarin-based hydroxamates as histone deacetylase inhibitors with Antitumor activities. Molecules 2020, 25. 10.3390/molecules25030717 PMC7036849 32046013

[37] Shan P, Yang F, Yu J, Wang L, Qu Y, Qiu H, Zhang H, Zhu S (2022). A novel histone deacetylase inhibitor exerts promising anti-breast cancer activity via triggering AIFM1-dependent programmed necrosis. Cancer Commun.

[38] Somech R, Izraeli S, Simon J (2004). Histone deacetylase inhibitors–a new tool to treat cancer. Cancer Treat Rev.

[39] Hosseini H, Obradovic MMS, Hoffmann M, Harper KL, Sosa MS, Werner-Klein M, Nanduri LK, Werno C, Ehrl C, Maneck M (2016). Early dissemination seeds metastasis in breast cancer. Nature.

[40] Zhong B-H, Dong M. The implication of ciliary signaling pathways for epithelial-mesenchymal transition. MOLECULAR AND CELLULAR BIOCHEMISTRY; 2023. 10.1007/s11010-023-04817-w PMC11224103 37490178

[41] Demirkan B (2013). The roles of epithelial-to-mesenchymal transition (EMT) and mesenchymal-to-epithelial transition (MET) in breast Cancer bone metastasis: potential targets for Prevention and Treatment. J Clin Med.

[42] Milde-Langosch K, Janke S, Wagner I, Schroder C, Streichert T, Bamberger A-M, Janicke F, Loning T (2008). Role of Fra-2 in breast cancer: influence on tumor cell invasion and motility. Breast Cancer Res Treat.

[43] Nakayama T, Hieshima K, Arao T, Jin Z, Nagakubo D, Shirakawa AK, Yamada Y, Fujii M, Oiso N, Kawada A (2008). Aberrant expression of Fra-2 promotes CCR4 expression and cell proliferation in adult T-cell leukemia. Oncogene.

[44] Cui S, Wu Q, Liu M, Su M, Liu S, Shao L, Han X, He H. EphA2 super-enhancer promotes tumor progression by recruiting FOSL2 and TCF7L2 to activate the target gene EphA2. CELL DEATH & DISEASE; 2021. p. 12. 10.1038/s41419-021-03538-6 PMC7955082 33712565

[45] Ji C, Hong X, Lan B, Lin Y, He Y, Chen J, Liu X, Ye W, Mo Z, She Z, Lin S (2021). Circ_0091581 promotes the Progression of Hepatocellular Carcinoma through Targeting miR-591/FOSL2 Axis. Dig Dis Sci.

[46] Millard CJ, Watson PJ, Celardo I, Gordiyenko Y, Cowley SM, Robinson CV, Fairall L, Schwabe JWR (2013). Class I HDACs share a common mechanism of regulation by Inositol Phosphates. Mol Cell.

[47] Lee J-H, Bollschweiler D, Schaefer T, Huber R (2021). Structural basis forthe regulation of nucleosome recognition and HDAC activity by histone deacetylase assemblies. Sci Adv.

[48] Turnbull RE, Fairall L, Saleh A, Kelsall E, Morris KL, Ragan TJ, Savva CG, Chandru A, Millard CJ, Makarova OV (2020). The MiDAC histone deacetylase complex is essential for embryonic development and has a unique multivalent structure. Nat Commun.

[49] Kaczynski J, Cook T, Urrutia R (2003). Sp1- and kruppel-like transcription factors. Genome Biol.

[50] Varshochi R, Halim F, Sunters A, Alao JP, Madureira PA, Hart SM, Ali S, Vigushin DM, Coombes RC, Lam EWF (2005). ICI182,780 induces p21Waf1 gene transcription through releasing histone deacetylase 1 and estrogen receptor alpha from Sp1 sites to induce cell cycle arrest in MCF-7 breast cancer cell line. J Biol Chem.

[51] Tan NY, Khachigian LM (2009). Sp1 phosphorylation and its regulation of gene transcription. Mol Cell Biol.

[52] Hung J-J, Wang Y-T, Chang W-C (2006). Sp1 deacetylation induced by phorbol ester recruits p300 to activate 12(S)-lipoxygenase gene transcription. Mol Cell Biol.

[53] Manikandan P, Nagini S (2018). Cytochrome P450 structure, function and clinical significance: a review. Curr Drug Targets.

[54] Deroanne CF, Bonjean K, Servotte S, Devy L, Colige A, Clausse N, Blacher S, Verdin E, Foidart JM, Nusgens BV, Castronovo V (2002). Histone deacetylases inhibitors as anti-angiogenic agents altering vascular endothelial growth factor signaling. Oncogene.

[55] Munster PN, Thurn KT, Thomas S, Raha P, Lacevic M, Miller A, Melisko M, Ismail-Khan R, Rugo H, Moasser M, Minton SE (2011). A phase II study of the histone deacetylase inhibitor vorinostat combined with tamoxifen for the treatment of patients with hormone therapy-resistant breast cancer. Br J Cancer.

[56] Sun YJ, Sun YY, Yue SC, Wang YH, Lu FH (2018). Histone deacetylase inhibitors in Cancer Therapy. Curr Top Med Chem.

[57] Parveen R, Harihar D, Chatterji BP (2023). Recent histone deacetylase inhibitors in cancer therapy. Cancer.

[58] Li J, Zhou L, Jiang H, Lin L, Li Y (2022). Inhibition of FOSL2 aggravates the apoptosis of ovarian cancer cells by promoting the formation of inflammasomes. Genes Genomics.

[59] Li S, Fang XD, Wang XY, Fei BY (2018). Fos-like antigen 2 (FOSL2) promotes metastasis in colon cancer. Exp Cell Res.

[60] Yin J, Hu W, Fu W, Dai L, Jiang Z, Zhong S, Deng B, Zhao J (2019). HGF/MET regulated epithelial-mesenchymal transitions and Metastasis by FOSL2 in Non-small Cell Lung Cancer. Onco Targets Ther.

