# Supplementary material for: Discovery of a novel hybrid coumarin-hydroxamate conjugate targeting the HDAC1-Sp1-FOSL2 signaling axis for breast cancer therapy
Source: Cell Commun Signal. 2024 Jul 15;22:361. doi: 10.1186/s12964-024-01733-4 (PMC11247895; doi:10.1186/s12964-024-01733-4)
Supplement: Supplementary file 1 — Supplementary Material 1 [file 12964_2024_1733_MOESM1_ESM.docx]

**Discovery of a novel hybrid coumarin-hydroxamate conjugate targeting the HDAC1-Sp1-FOSL2 signaling axis for breast cancer therapy**

Sujie Zhu^a,1^, Wenjing Zhu^b,1^, Kaihua Zhao^c,1^, Jie Yu^c^, Wenxia Lu^d^, Rui Zhou^e^, Shule Fan^d^, Weikaixin Kong^f,g*^, Feifei Yang^d,*^, Peipei Shan^a,*^

^a^ Institute of Translational Medicine, The Affiliated Hospital of Qingdao University, College of Medicine, Qingdao University. Qingdao 266021, China

^b^ Clinical Research Center, Qingdao Municipal Hospital, Qingdao 266071, China

^c^ Qingdao Central Hospital, University of Health and Rehabilitation Sciences (Qingdao Central Hospital）, Qingdao 266042, China

^d^ School of Biological Science and Technology, University of Jinan, Jinan 250022, China

^e^ Department of Marine Bio-Pharmacology, College of Food Science and Technology, Shanghai Ocean University, Shanghai 201306, China.

^f^ Department of Molecular and Cellular Pharmacology, School of Pharmaceutical Sciences, Peking University Health Science Center, Beijing 100191, China;

^g^ Institute for Molecular Medicine Finland (FIMM), HiLIFE, University of Helsinki, Helsinki 00250, Finland)

^1^ These authors contributed equally: Su-jie Zhu, Wen-jing Zhu, Kai-hua Zhao

Correspondence: Peipei Shan ([shanpeipei@qdu.edu.cn](mailto:shanpeipei@qdu.edu.cn)) or Feifei Yang (bio_yangff@ujn.edu.cn) or Weikaixin Kong ([1510307407@pku.edu.cn](mailto:1510307407@pku.edu.cn))

**Table of Contents**

Figure S1. Synthesis of compound ZN444B.

Figure S2. ZN444B retards cell growth and metastasis of breast cancer cells.

Figure S3. ZN444B shows stronger anti-breast cancer activity than SAHA *in vitro*.

Figure S4. The protein expression of FOSL2 is significantly decreased by ZN444B treatments in MDA-MB231 cells.

Figure S5. ZN444B shows no potential toxicity on mice.

Figure S6. ZN444B exerts stronger anti-breast cancer activity than YF349.

Figure S7. Coumarin shows no effects on the expression of FOSL2 and HDAC1.

**Table S1.** The percentages of tissues with different levels of staining for FOSL2 in normal ductal tissues and breast cancer tissues at different tumor stages.

**Table S2.** Routine blood chemistry analysis of DMSO (vehicle) or ZN444B treated mice.

**Supplementary Fig. S1**

**
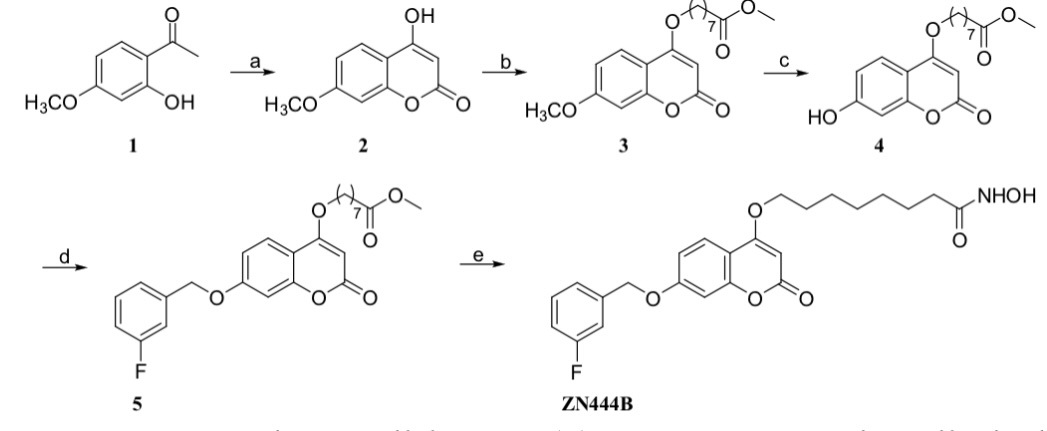
**

**Reagents and conditions:** (a) NaH, CH_3_Ph, diethyl carbonate, 115 °C, reflux; (b) Br(CH_2_)_7_CO_2_CH_3_, K_2_CO_3_, DMF; (c) BBr_3_, DCM; (d) RBr, K_2_CO_3_, DMF; (e) NH_2_OH·HCl, KOH, MeOH.

**Supplementary Fig. S1 Synthesis of compound ZN444B.** The synthetic routes of compound ZN444B was shown in Scheme 1. Intermediate 2 was prepared by a ring-closing reaction using starting materials paeonol 1 and diethyl carbonate, which was subsequently reacted with methyl 8-bromooctanoate in DMF to give compound 3. The resulting ester was demethylated with BBr_3_ to afford intermediate 4, an aromatic nucleophilic substitution reaction was performed to give compound 5. Finally, 5 was converted to target compound ZN444B by hydroxylamine treatment under basic conditions. The relevant characterization data of this compound can be found in the previous published article.

**Supplementary Fig. S2**

**
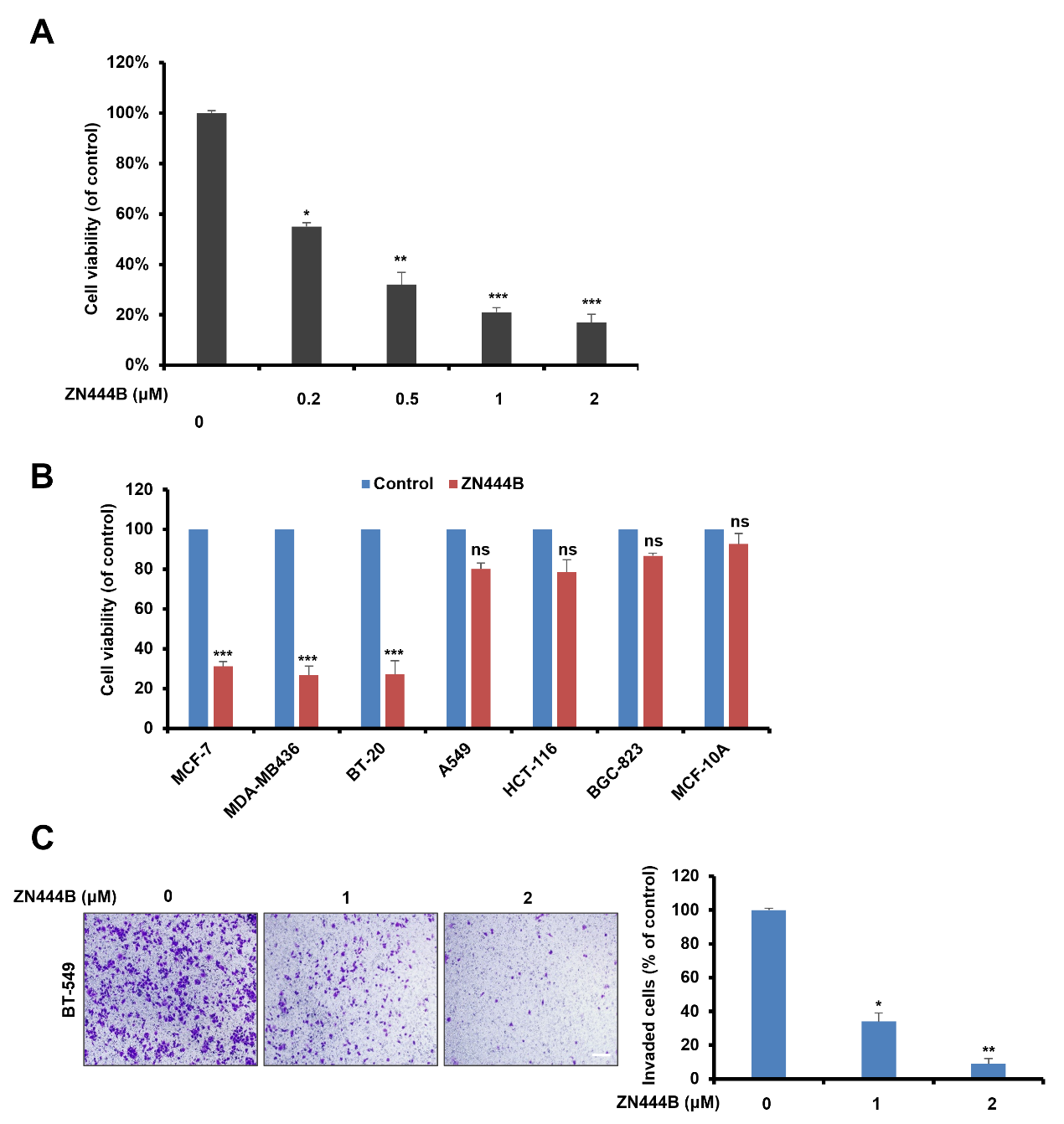
**

**Supplementary Fig. S2 ZN444B retards cell growth and metastasis of breast cancer cells. A** A panel of tumor cell lines and MCF10A were treated with ZN444B, after 48 h, the MTS assay was performed. The bars indicate the mean ± SD. **B** A panel cell lines (MDA231, 4T1, BT549, MDA-MB468, HCC-1937, A549, H1299, HCT116 and MCF10A) were treated with ZN444B (0.5 μM). After 48 h, the MTS assay was performed.BT-549 cells were treated with ZN444B in the indicated concentration, after 48 h, the MTS assay was performed. The bars indicate the mean ± SD. **C** BT-549 cells were treated with different concentrations of ZN444B and allowed to invade through matrigel. Images were obtained after 12 h of incubation (upper). Invaded cell number was counted and expressed as % untreated control (lower) (scale bar 100 μm). Data show the mean ± SD from three independent experiments.

**Supplementary Fig. S3**

**
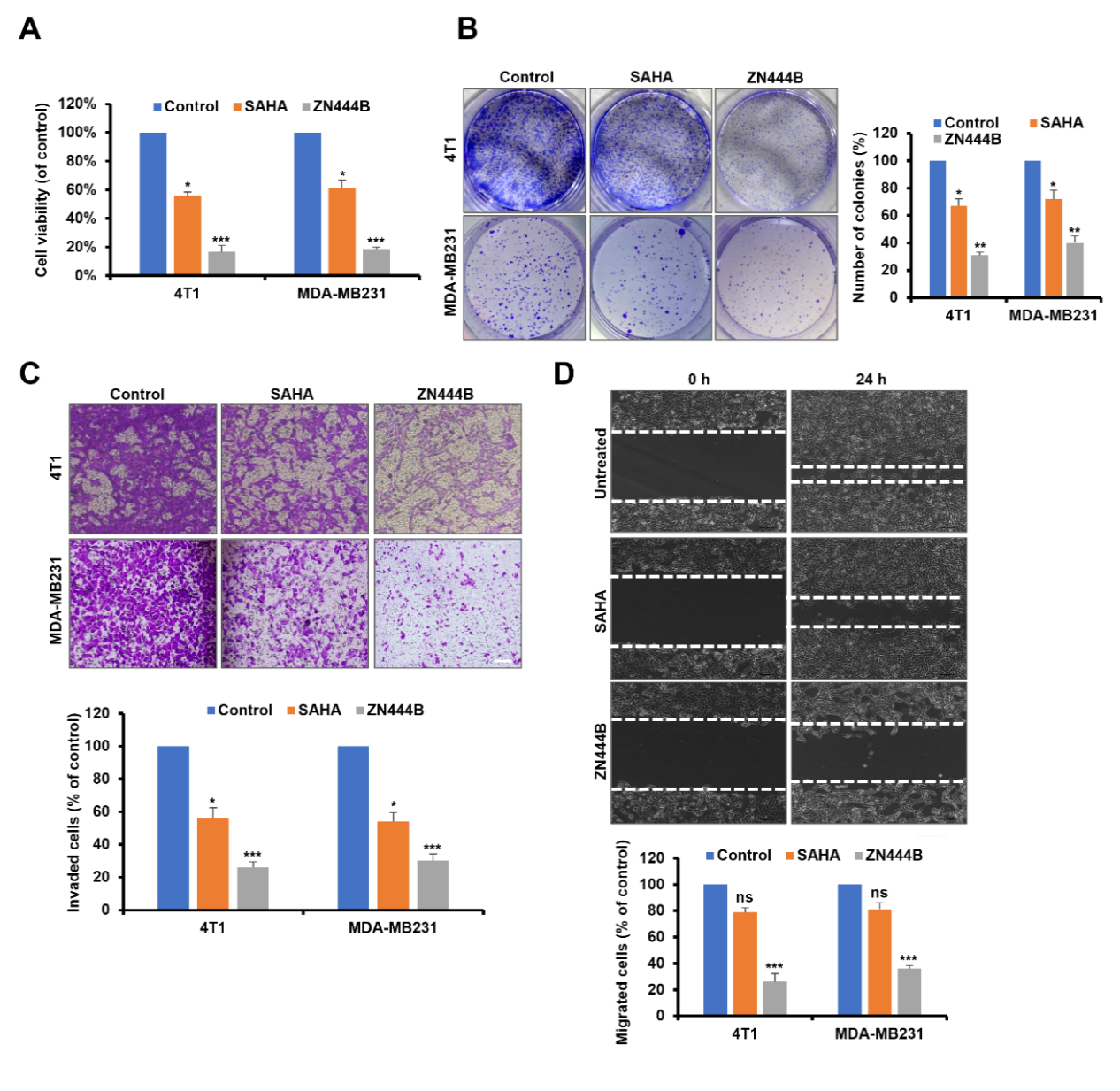
**

**Supplementary Fig. S3. ZN444B shows stronger anti-breast cancer activity than SAHA *in vitro*. A** 4T1 and MDA-MB231 cells were treated with ZN444B (1 μM) or SAHA (1 μM), after 48 h, the MTS assay was performed. The bars indicate the mean ± SD. **B** 4T1 and MDA-MB231 cells were seeded on 6 well plates. After 12 h, cells were treated with ZN444B (1 μM) or SAHA (1 μM). On day 10, Number of colonies was counted in experiments repeated three times. Results represent the average of three replications. **C** 4T1 and MDA-MB231 cells were treated with ZN444B (1 μM) or SAHA (1 μM) and allowed to invade through the matrigel. Images were obtained after 12 h of incubation (upper). Invaded cell number was counted and expressed as % untreated control (lower). Data show the mean ± SD from three independent experiments. **D** 4T1 and MDA-MB231 cells were seeded in six-well plates. A “wound” was created after the cells grew into full confluence, then ZN444B (1 μM) or SAHA (1 μM) were added. Images were taken after 12 h of incubation at 37°C.

**Supplementary Fig. S4**

**
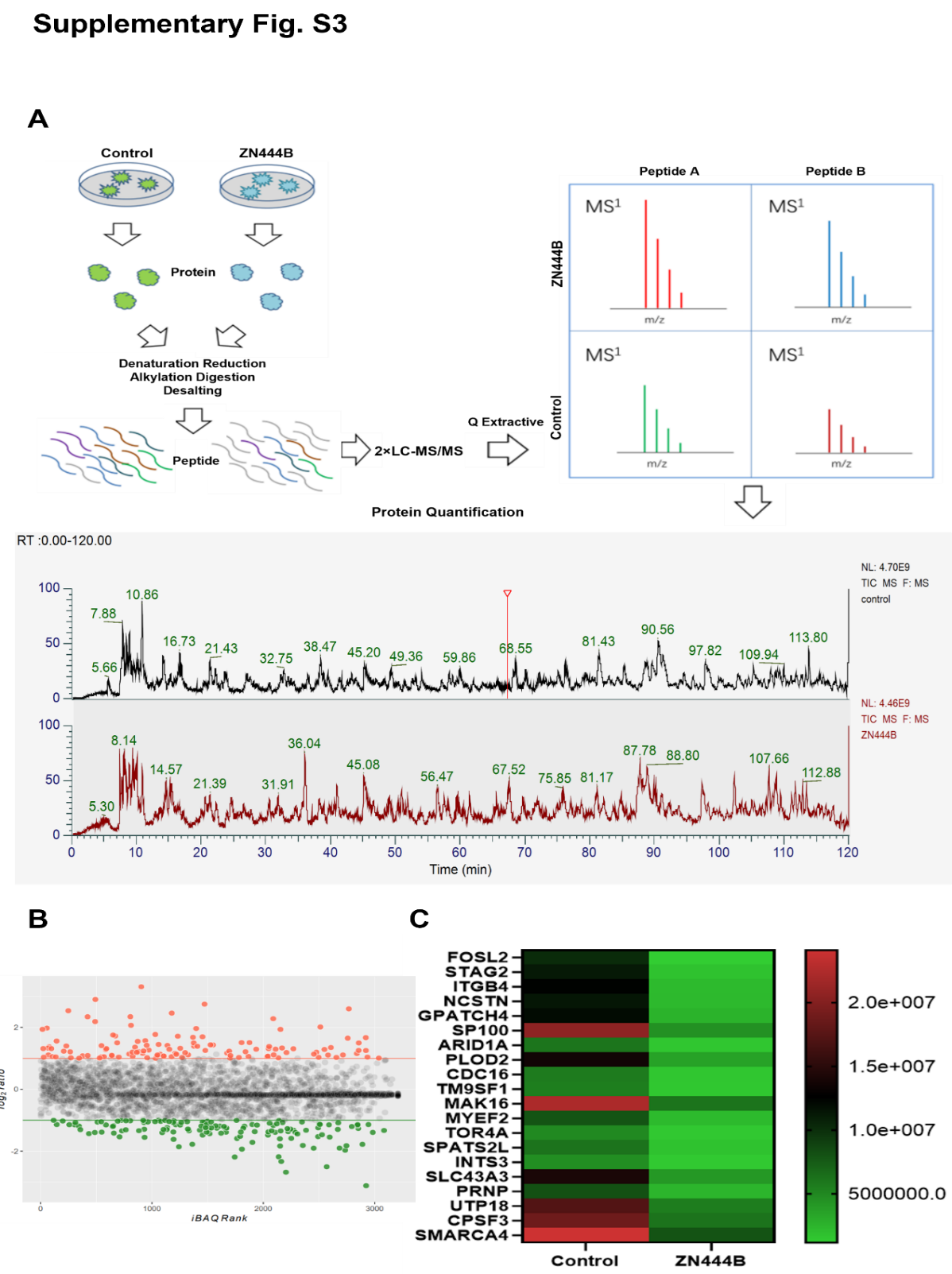
**

**Supplementary Fig. S4 The protein expression of FOSL2 is significantly decreased by ZN444B treatments in MDA-MB231 cells. A** Protocol for LC-MS/MS Analysis of MDA-MB231 cells treated with ZN444B or not. **B** Volcano plot shows the significantly upregulated and downregulated proteins in MDA-MB231 cells treated with ZN444B or not by LC/MS-MS. **C** Heatmap of the top 20 downregulated proteins in MDA-MB231 cells treated with ZN444B or not by LC/MS-MS.

**Supplementary Fig. S5**

**
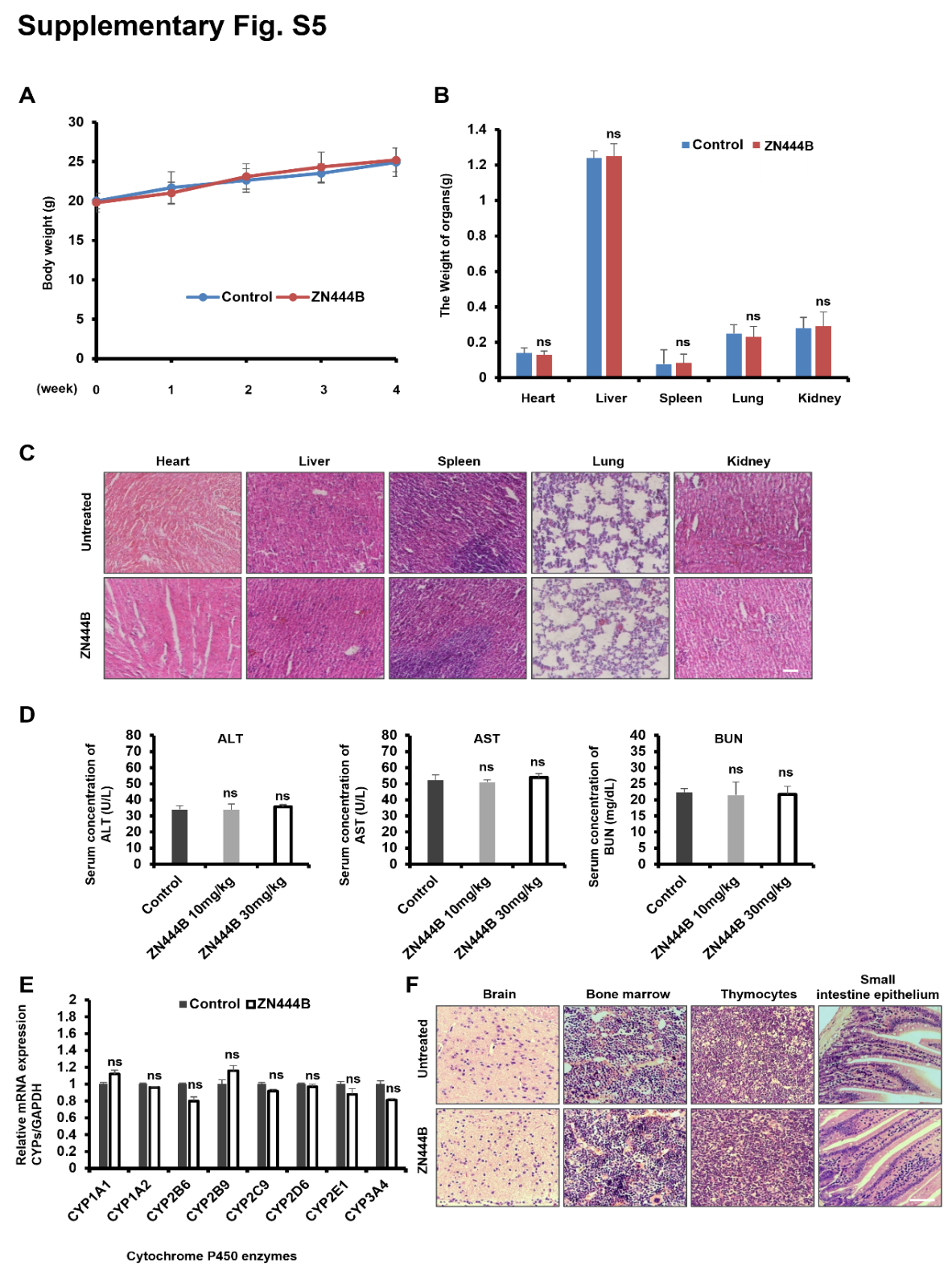
**

**Supplementary Fig. S5 ZN444B shows no potential toxicity on mice. A** ZN444B was administrated at the dose of 30 mg/kg each day for 28 days. Mice body weight was monitored once a week. **B** Major organs weight was evaluated when all mice were executed. **C** Major organs from the different groups of animals were stained with H&E (scale bar 100 μm). **D** BALB/c mice were daily administrated with PBS (vehicle) or ZN444B (10, 30 mg/kg) for 28 days. Mice were sacrificed 24 h after the last treatment. Each treatment group consisted of six mice. Blood samples were collected to Serum concentrations of ALT, AST and BUN were measured using Fuji DRI-CHEM 7000i (Fujifilm). **E** BALB/c mice were daily administrated with PBS (vehicle) or ZN444B (30 mg/kg) for 28 days. Mice were sacrificed 24 h after the last treatment and the liver was immediately excised to prepare total RNA. The expression of the Cyp1A1, Cyp1A2, Cyp2B6, Cyp2B9, Cyp2C9, Cyp2D6, Cyp2E1, and Cyp3A4 mRNA were performed using real-time RT-PCR and normalized to those of GAPDH mRNA. **F** ZN444B was administrated at the dose of 30 mg/kg each day for 60 days. Major organs from the different groups of animals were stained with H&E (scale bar 100 μm).

**Supplementary Fig. S6**

**
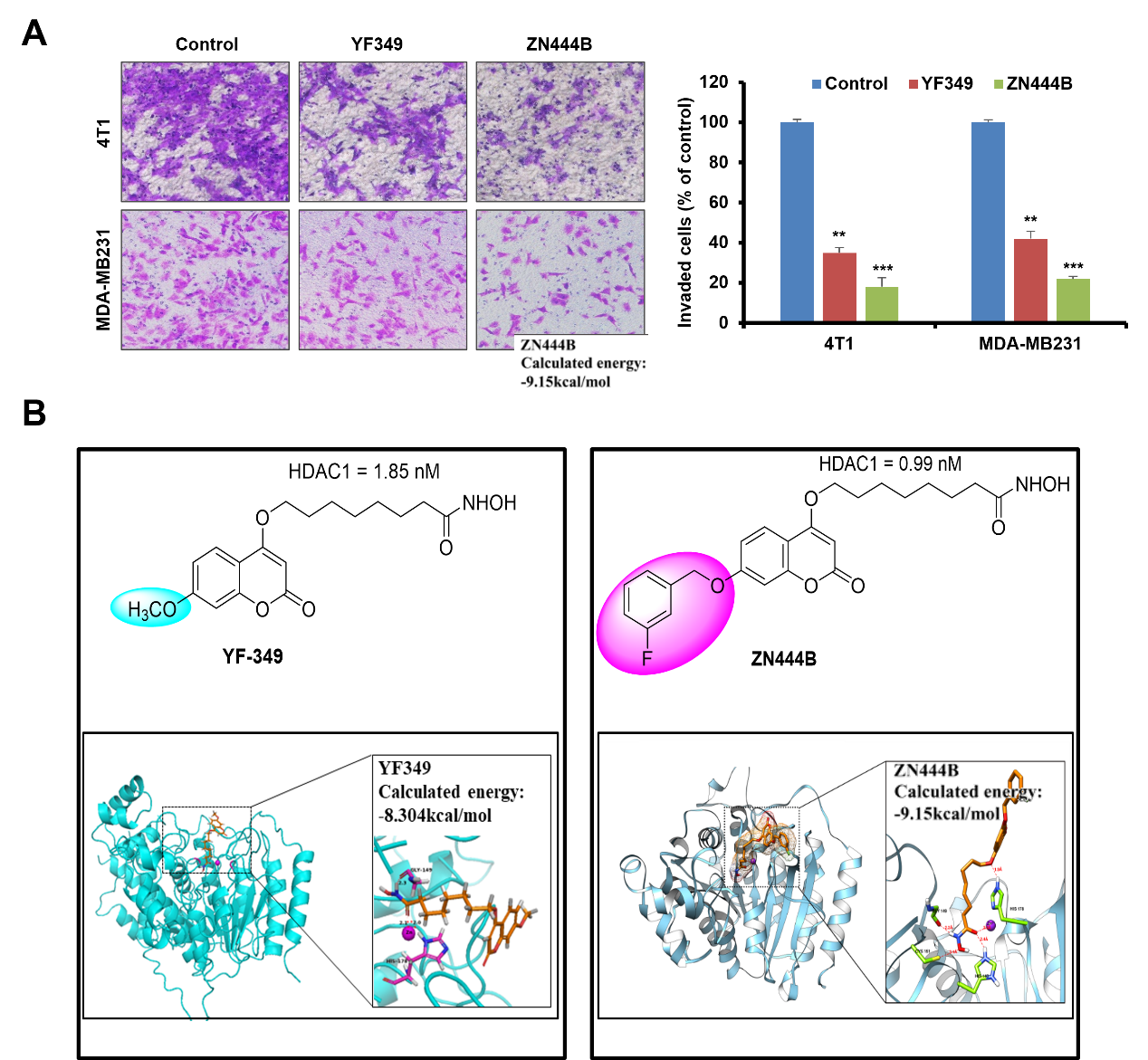
**

**Supplementary Fig. S6 ZN444B exerts stronger anti-breast cancer activity than YF349. A** 4T1 and MDA-MB231 cells were treated with YF349 or ZN444B (0.5 μM), then cells were allowed to invade through matrigel. Images were obtained after 12 h of incubation (upper). Invaded cell number was counted and expressed as % untreated control (lower). Data show the mean ± SD from three independent experiments. **B** Molecular surface of the HDAC1 binding pocket with YF349 and ZN444B.

**Supplementary Fig. S7**

**
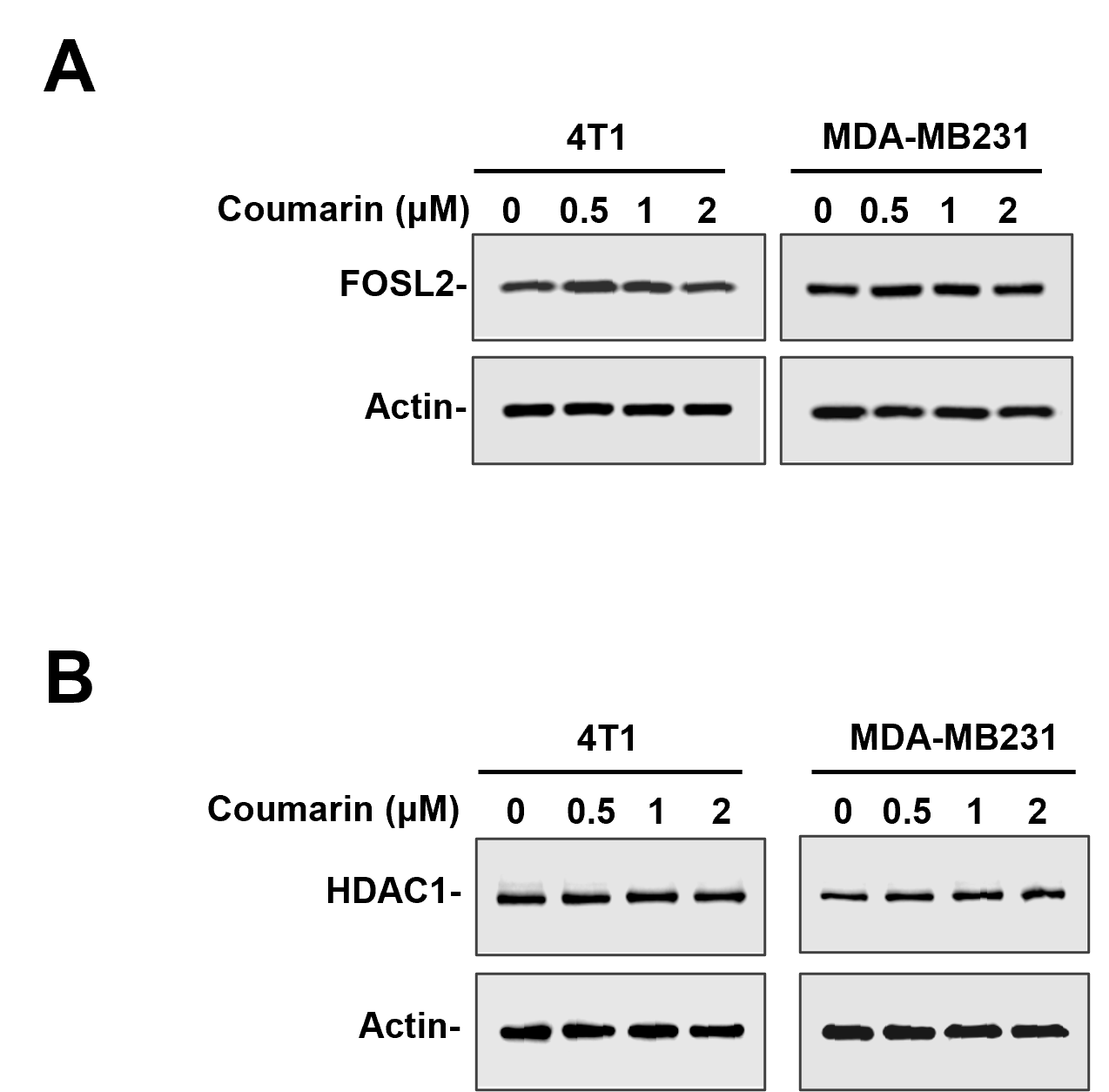
**

**Supplementary Fig. S7 Coumarin shows no effects on the expression of FOSL2 and HDAC1. A** MDA-MB231 and 4T1 cells were incubated with various concentrations of coumarin for 24 h. Effects on the expression of FOSL2 was determined by western blot. Actin was used as a loading control. **B** MB231 and 4T1 cells were incubated with various concentrations of coumarin for 24 h. Effects on the expression of HDAC1 was determined by western blot. Actin was used as a loading control.

**Supplementary Table S1**
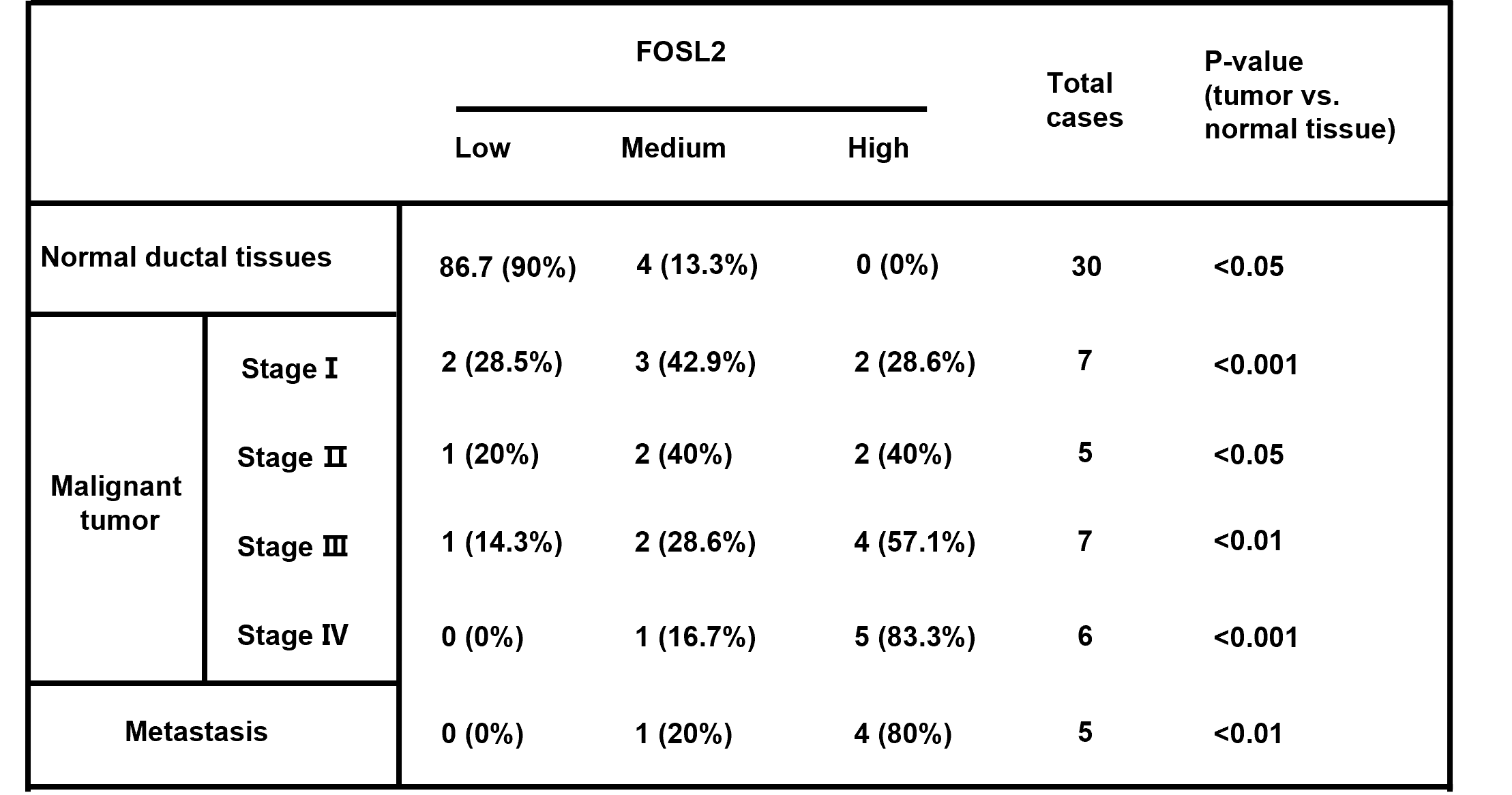


**Supplementary Table S1.** The percentages of tissues with different levels of staining for FOSL2 in normal ductal tissues and breast cancer tissues at different tumor stages.**Supplementary Table S2**
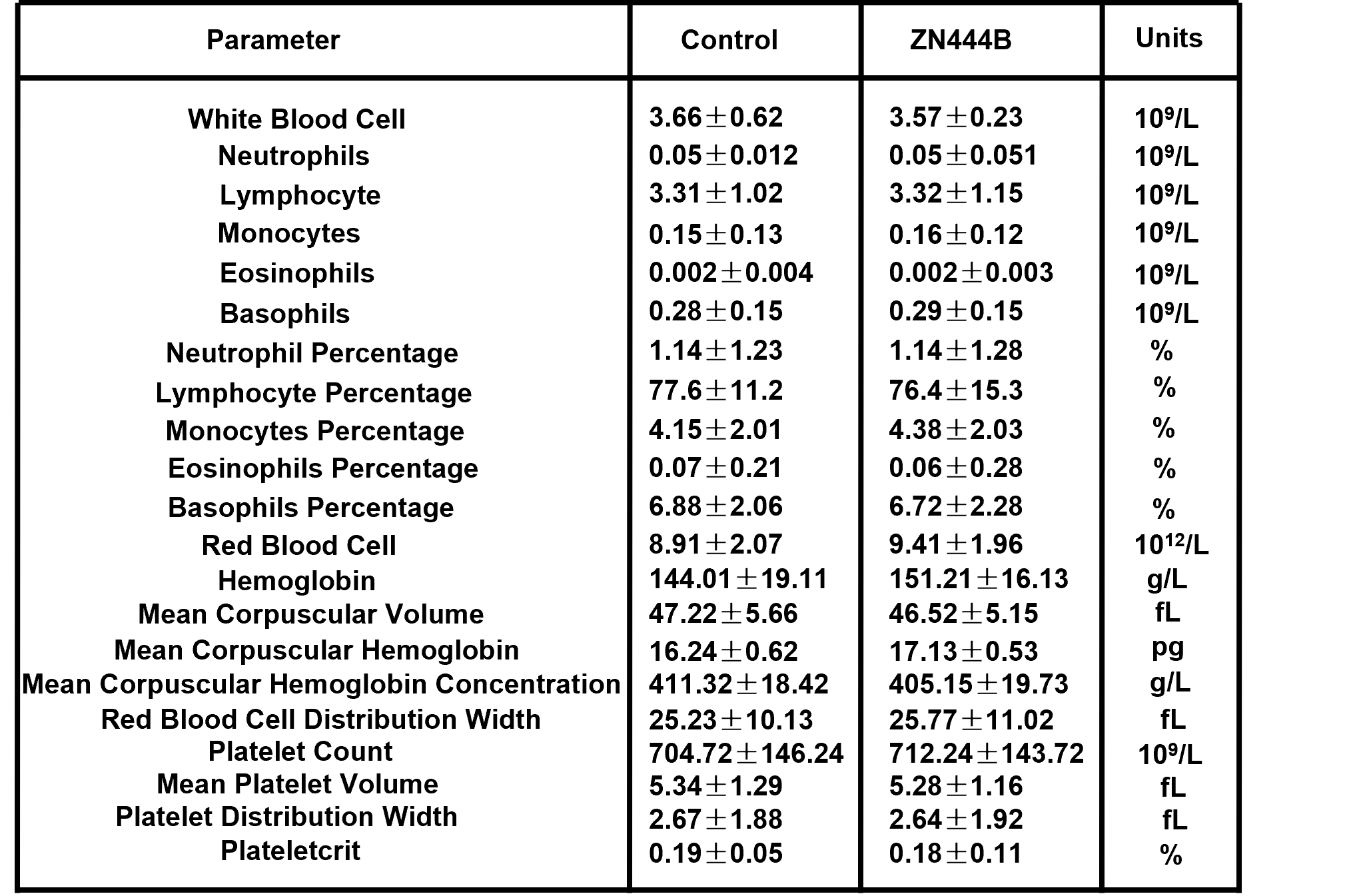


**Supplementary Table S2. Routine blood chemistry analysis of DMSO (vehicle) or ZN444B treated mice.** BALB/c mice were daily administrated with DMSO (vehicle) or ZN444B (30 mg/kg) for 28 days. Mice were sacrificed 24 h after the last treatment. Blood samples from each mouse were collected for blood chemistry analysis. Each treatment group consisted of six mice.
