# Supplementary material for: Discovery of a novel hybrid coumarin-hydroxamate conjugate targeting the HDAC1-Sp1-FOSL2 signaling axis for breast cancer therapy
Source: Cell Commun Signal. 2024 Jul 15;22:361. doi: 10.1186/s12964-024-01733-4 (PMC11247895; doi:10.1186/s12964-024-01733-4)

Figure 1F

Protein marker (#26616，Thermo)

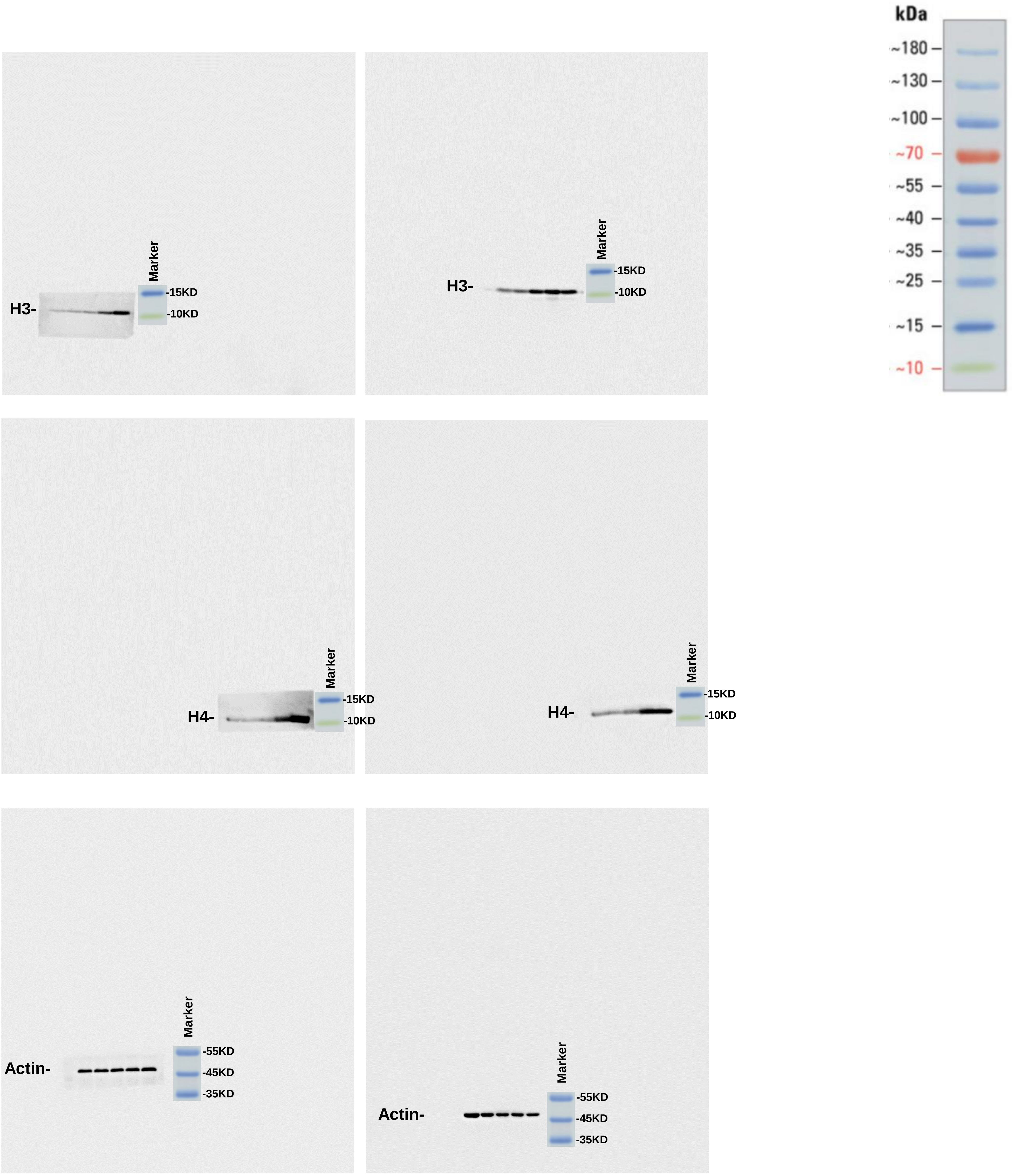

Figure 1I

Protein marker (#26616，Thermo)

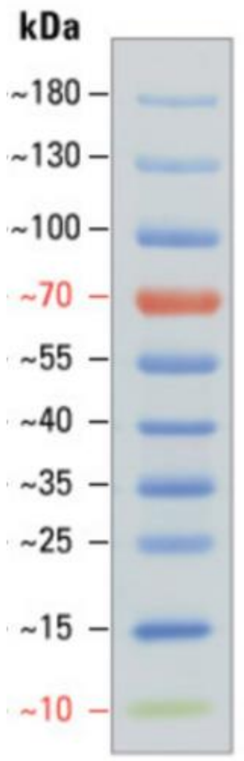

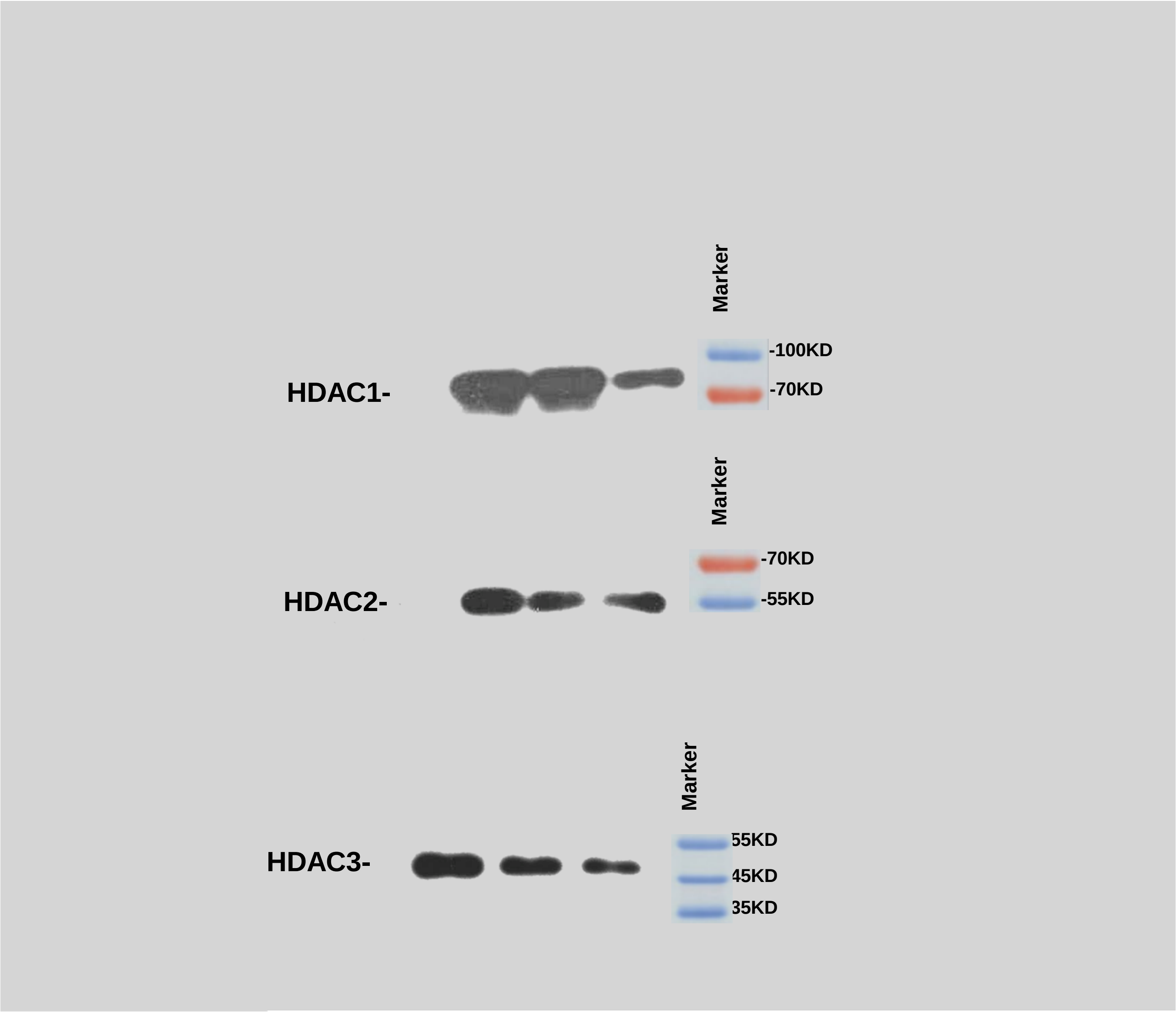

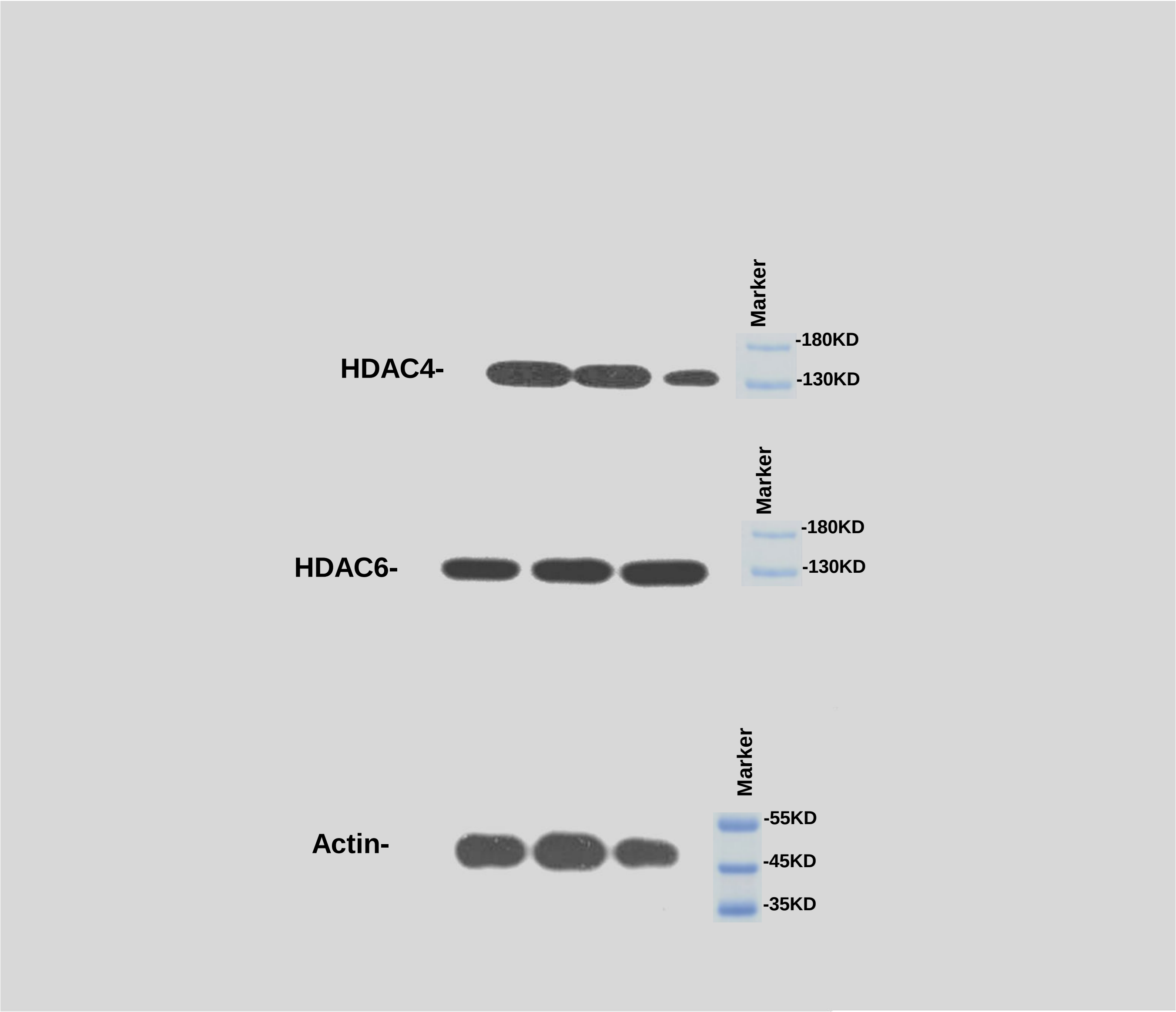

Figure 1N

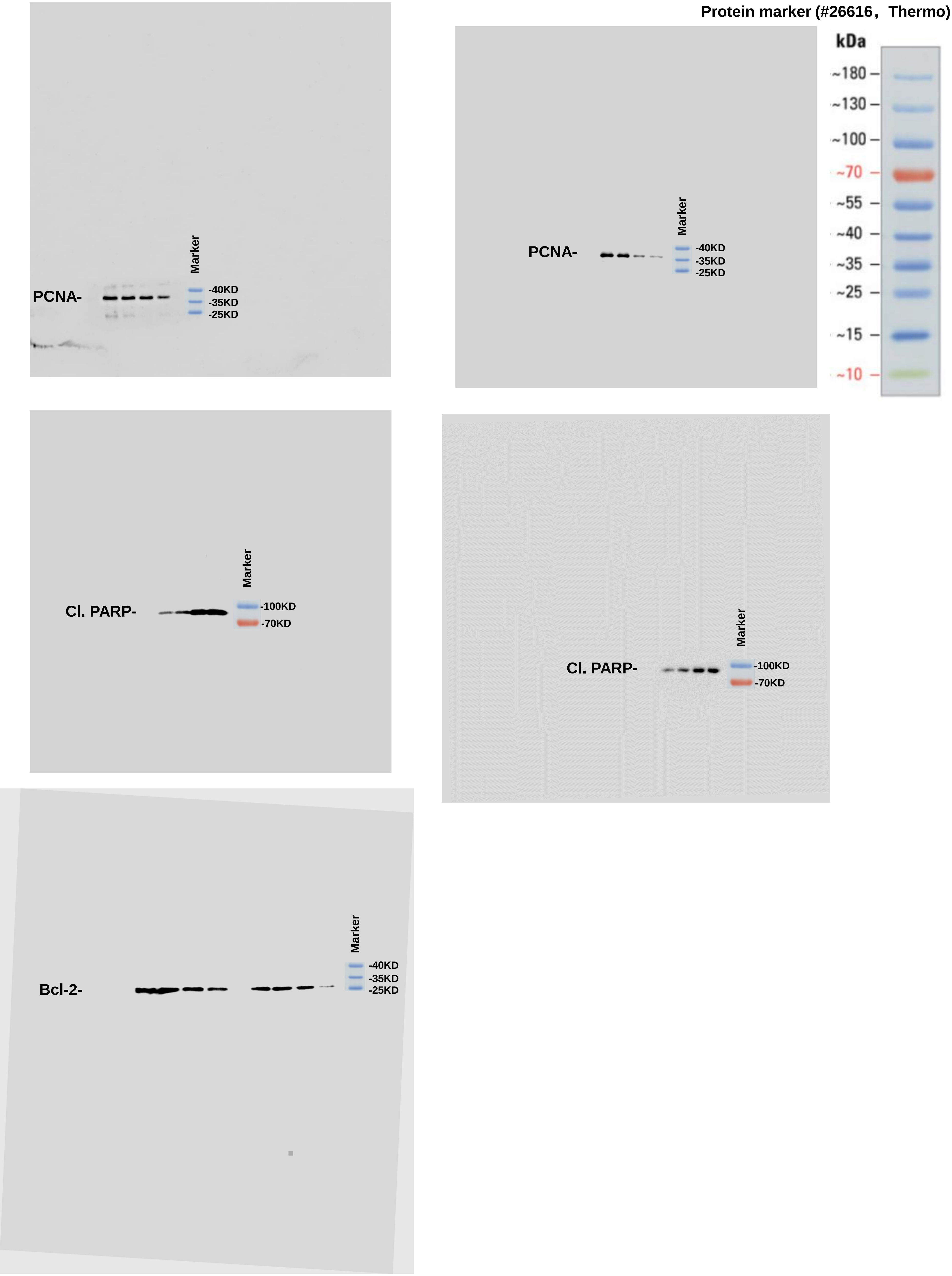

Protein marker (#26616，Thermo)
kDa
~180
~130
~100
~70
~55
~40
~35
~25
~15
~10
PCNA-
Marker
-40KD
-35KD
-25KD
PCNA-
Marker
-40KD
-35KD
-25KD
Cl. PARP-
Marker
-100KD
-70KD
Cl. PARP-
Marker
-100KD
-70KD
Bcl-2-
Marker
-40KD
-35KD
-25KD

Figure 1N

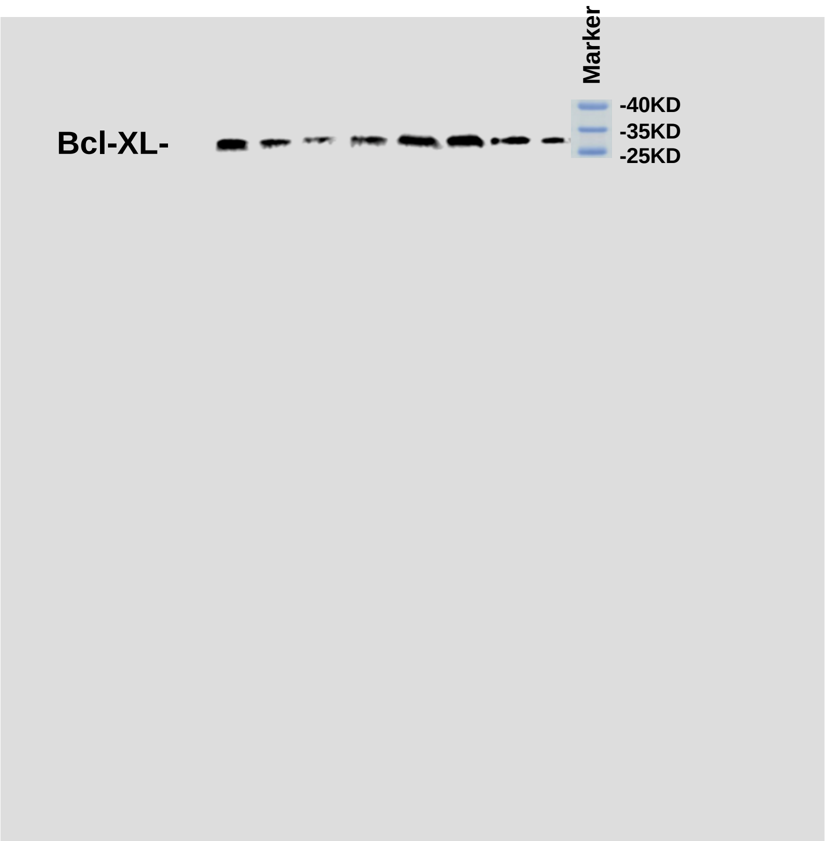

Marker
Bcl-XL-
-40KD
-35KD
-25KD

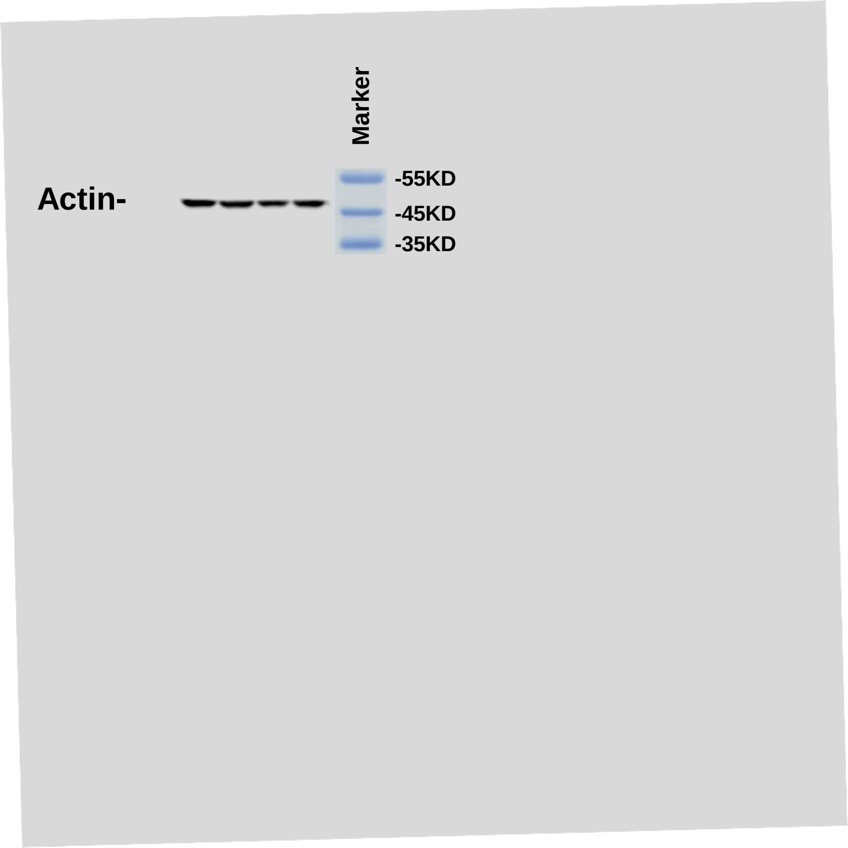

Marker
Actin-
-55KD
-45KD
-35KD

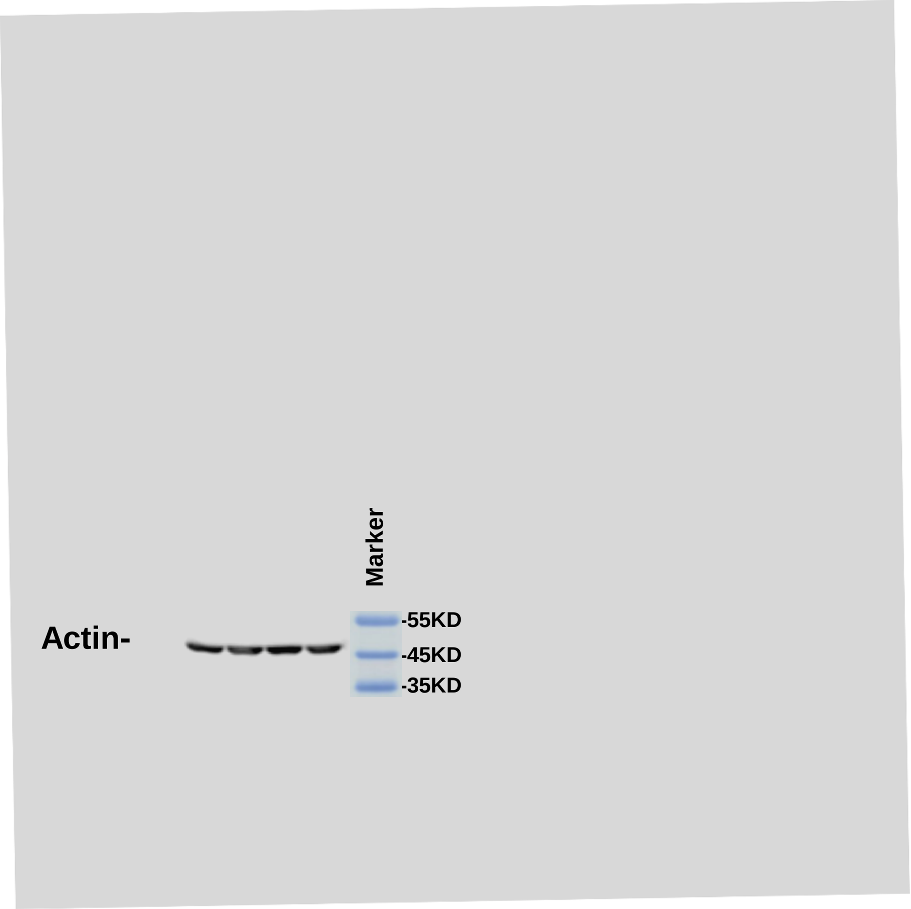

Marker
Actin-
-55KD
-45KD
-35KD

Figure 2D

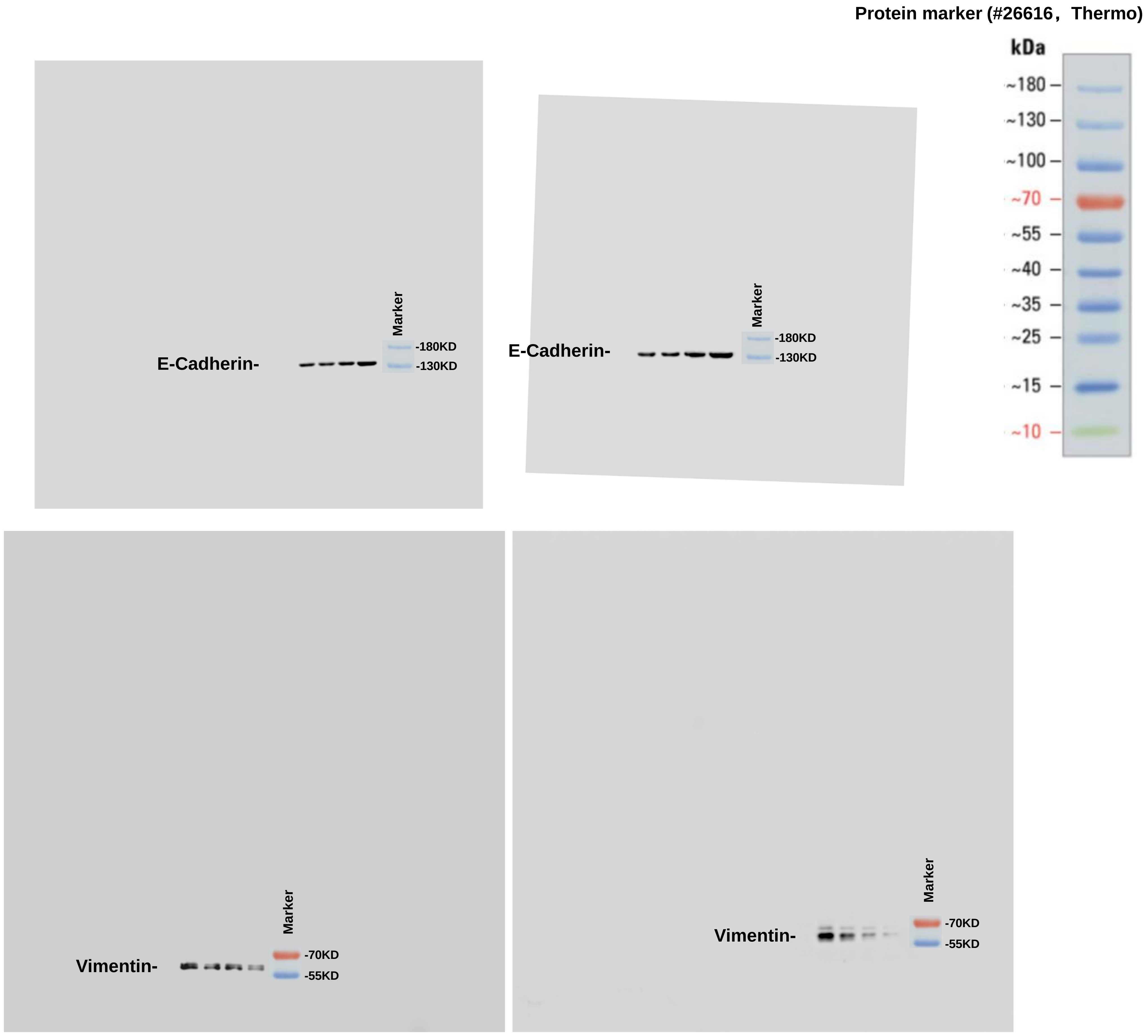

Protein marker (#26616，Thermo)
kDa
~180
~130
~100
~70
~55
~40
~35
~25
~15
~10
Marker
E-Cadherin-
-180KD
-130KD
Marker
E-Cadherin-
-180KD
-130KD
Marker
Vimentin-
-70KD
-55KD
Marker
Vimentin-
-70KD
-55KD

Figure 2D

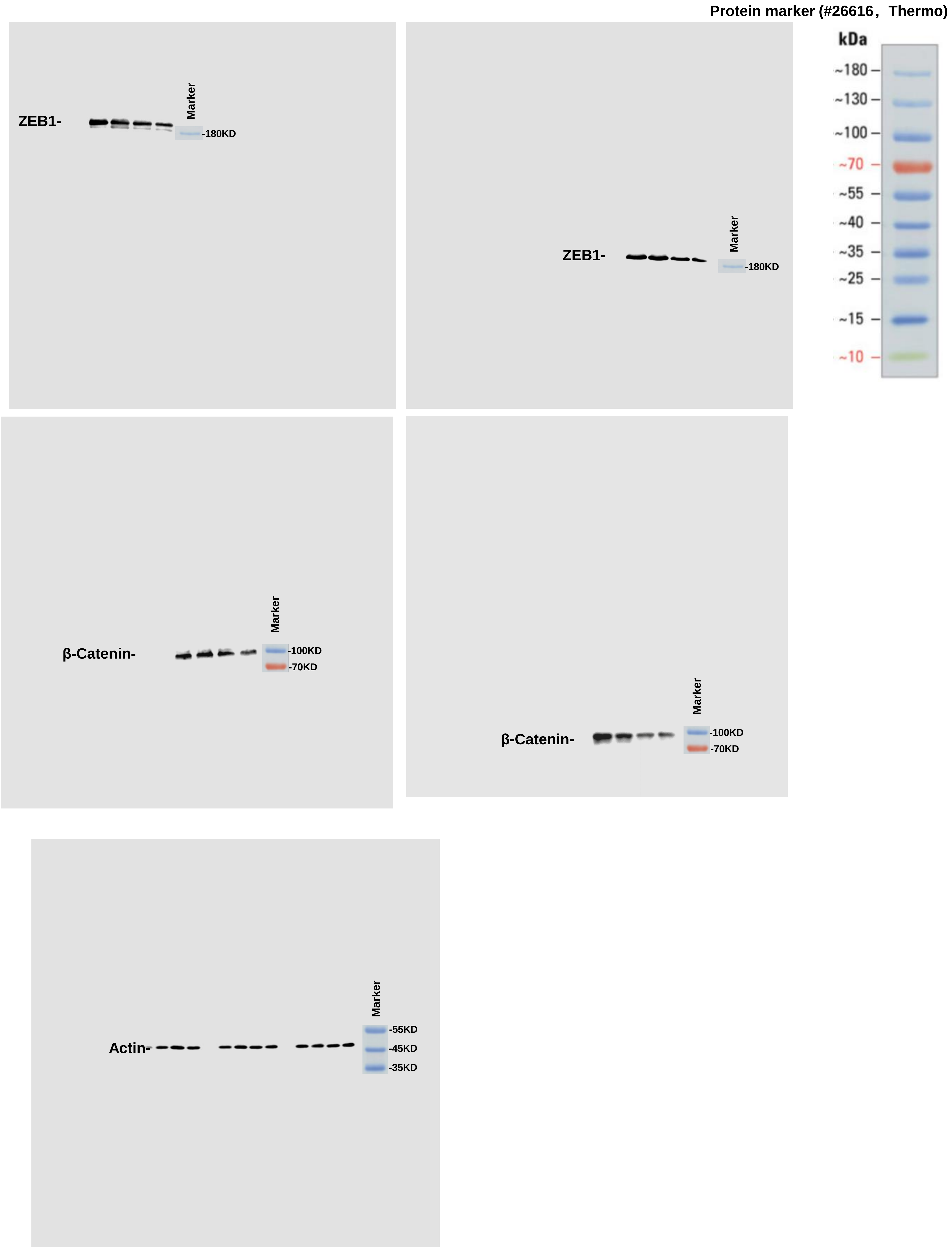

Protein marker (#26616，Thermo)
kDa
~180
~130
~100
~70
~55
~40
~35
~25
~15
~10
ZEB1-
Marker
-180KD
ZEB1-
Marker
-180KD
β-Catenin-
Marker
-100KD
-70KD
β-Catenin-
Marker
-100KD
-70KD
Actin-
Marker
-55KD
-45KD
-35KD

Figure 2I

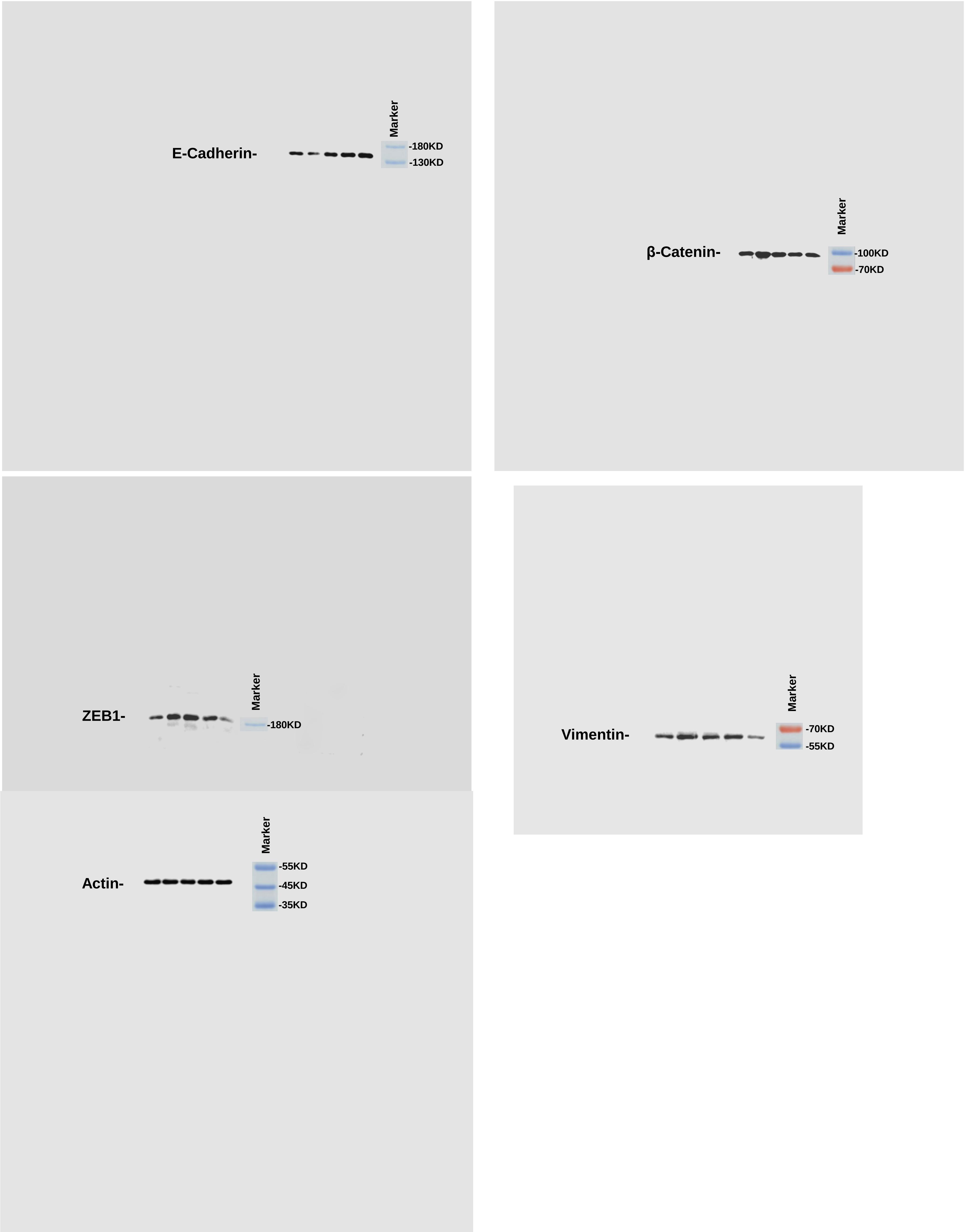

E-Cadherin-
Marker
-180KD
-130KD
β-Catenin-
Marker
-100KD
-70KD
ZEB1-
Marker
-180KD
Vimentin-
Marker
-70KD
-55KD
Actin-
Marker
-55KD
-45KD
-35KD

Figure 3G

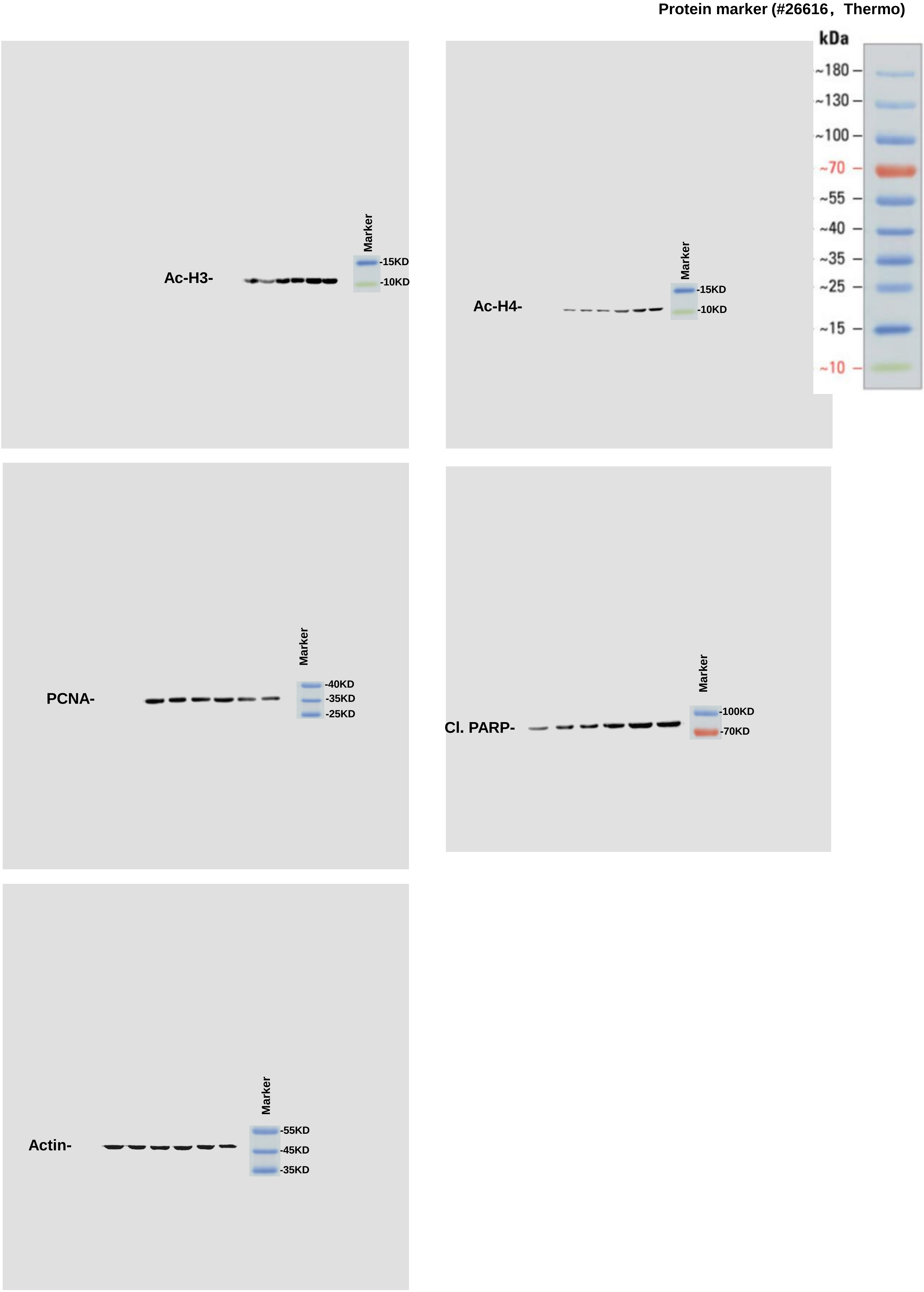

Protein marker (#26616，Thermo)
Ac-H3-
Marker
-15KD
-10KD
Ac-H4-
Marker
-15KD
-10KD
kDa
~180
~130
~100
~70
~55
~40
~35
~25
~15
~10
PCNA-
Marker
-40KD
-35KD
-25KD
Cl. PARP-
Marker
-100KD
-70KD
Actin-
Marker
-55KD
-45KD
-35KD

Figure 3O

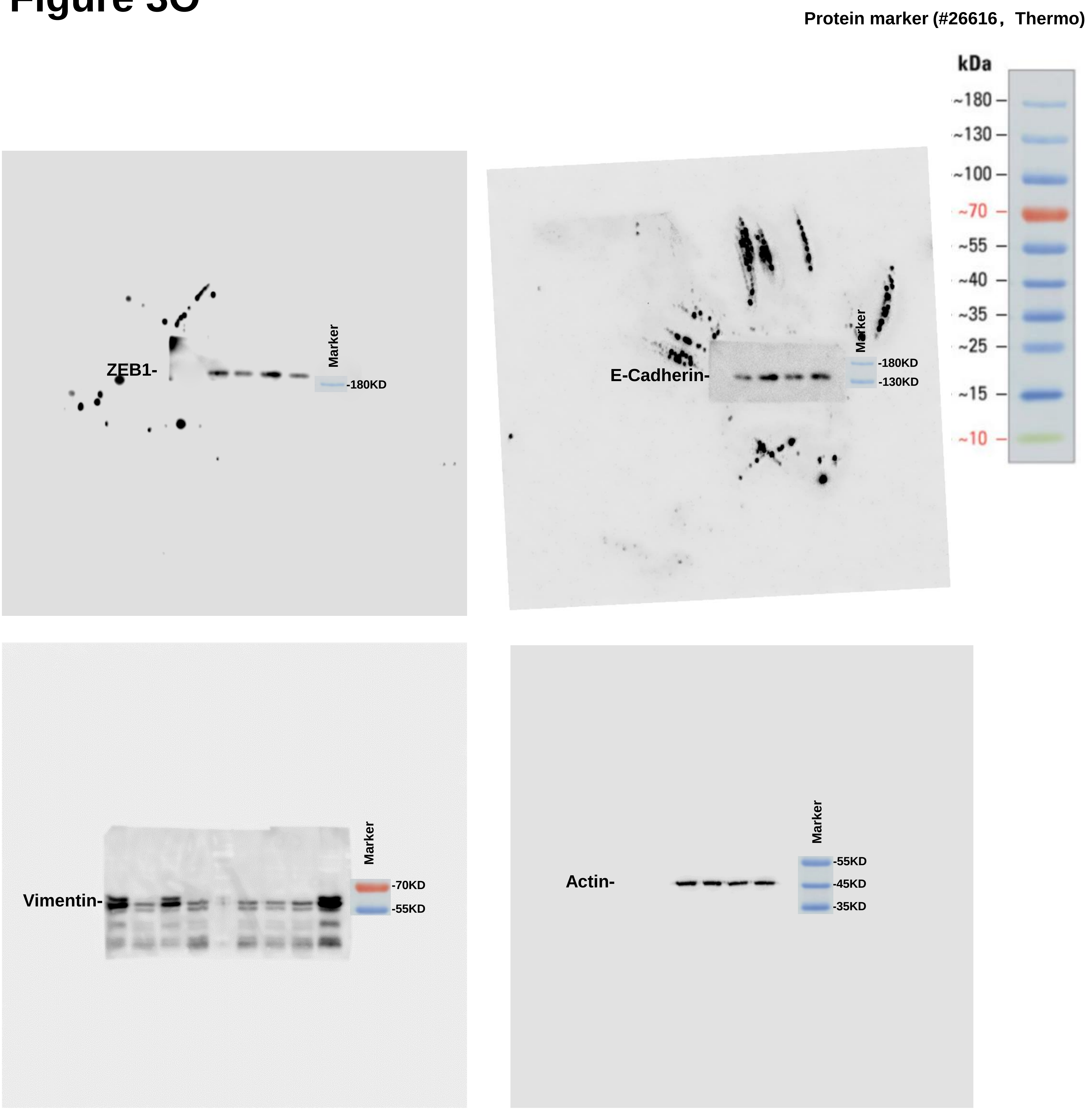
Protein marker (#26616，Thermo)
kDa
~180
~130
~100
~70
~55
~40
~35
~25
~15
~10
ZEB1-
Marker
-180KD
E-Cadherin-
Marker
-180KD
-130KD
Vimentin-
Marker
-70KD
-55KD
Actin-
Marker
-55KD
-45KD
-35KD

Figure 4C

Protein marker (#26616，Thermo)

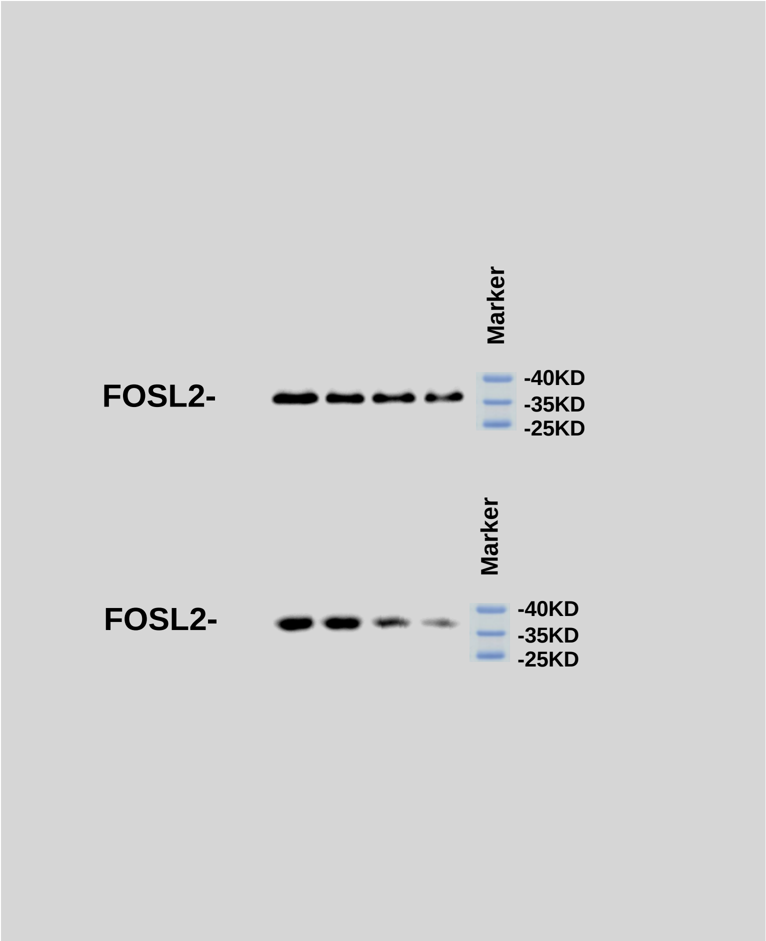

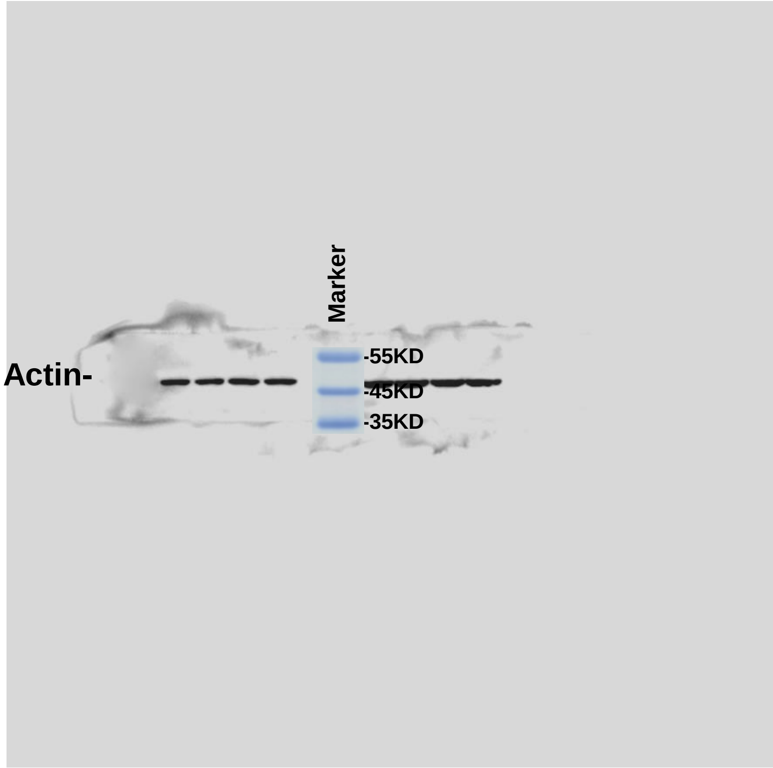

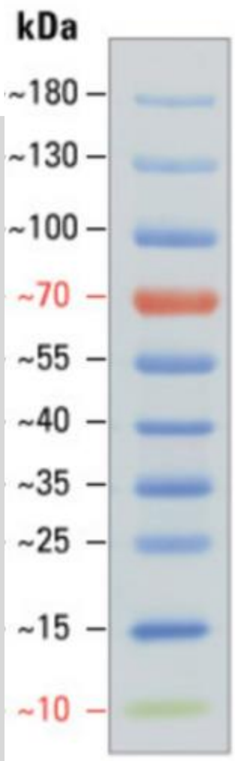

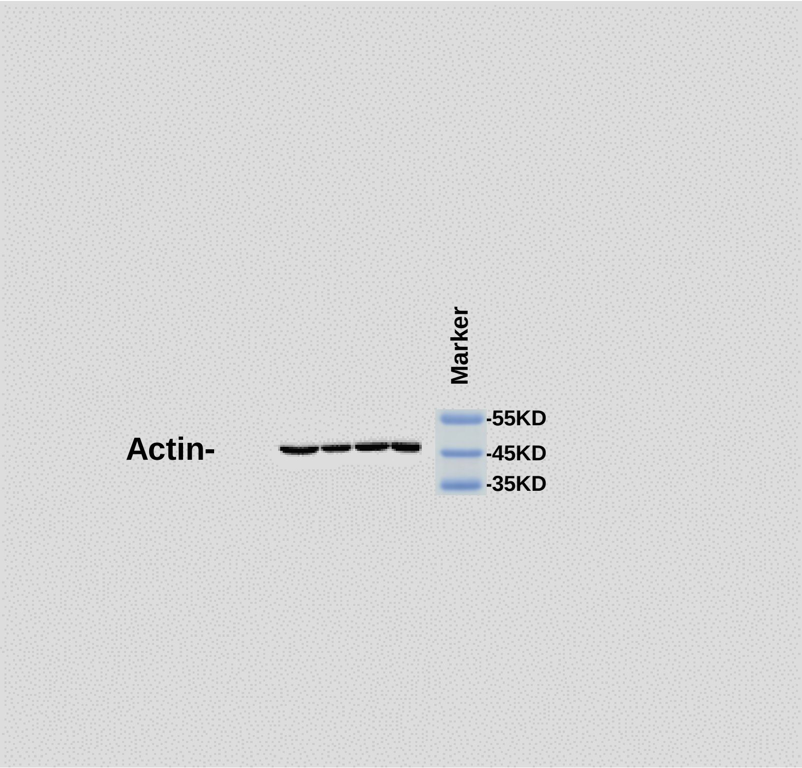

Figure 4F

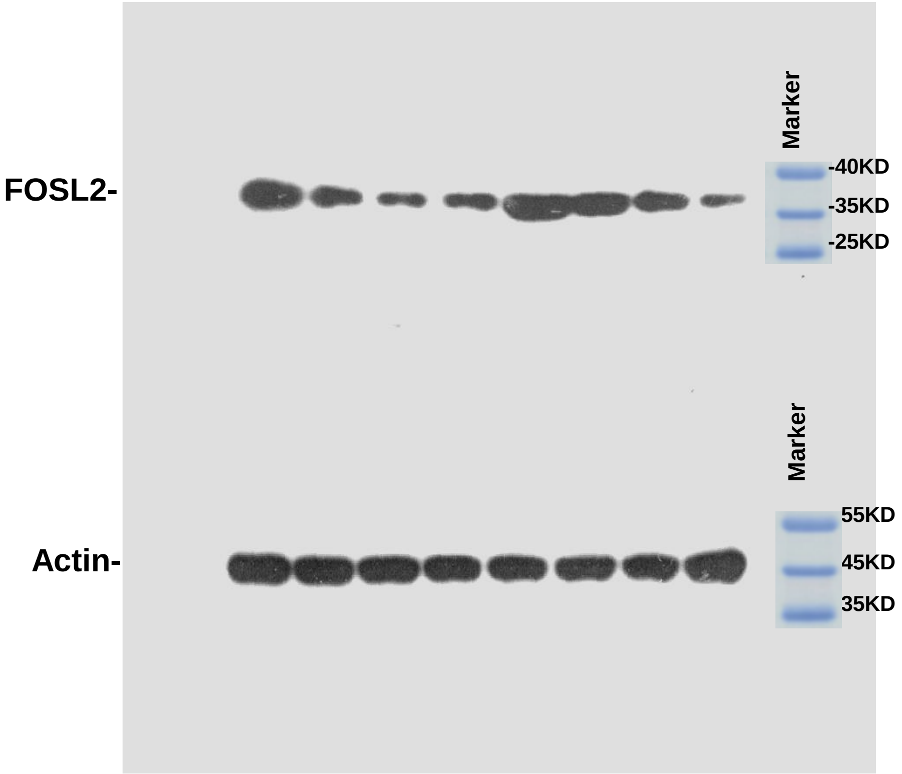

Protein marker (#26616，Thermo)

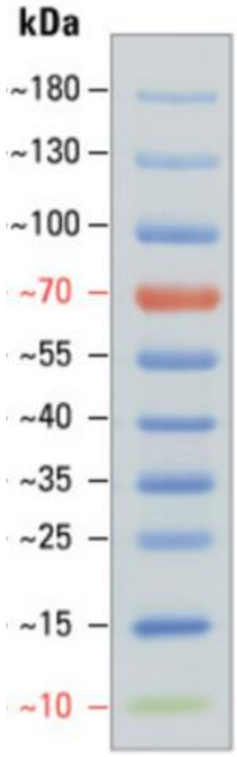

Figure 4H

Protein marker (#26616，Thermo)

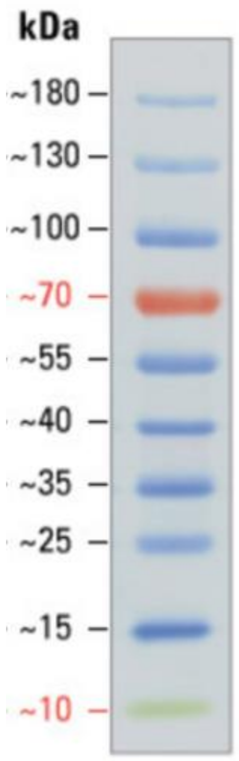

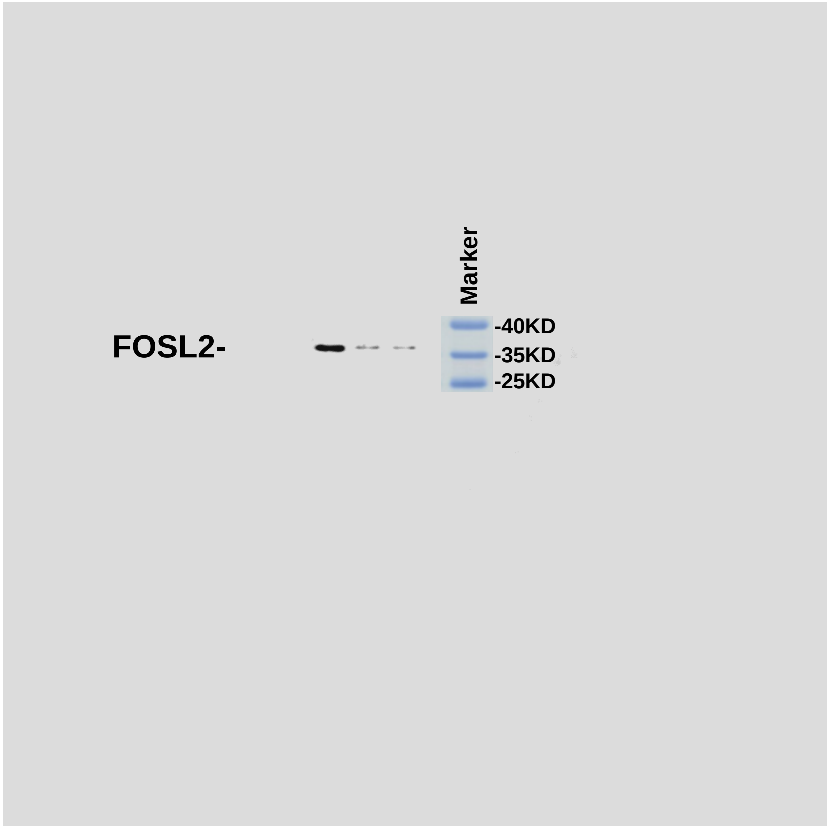

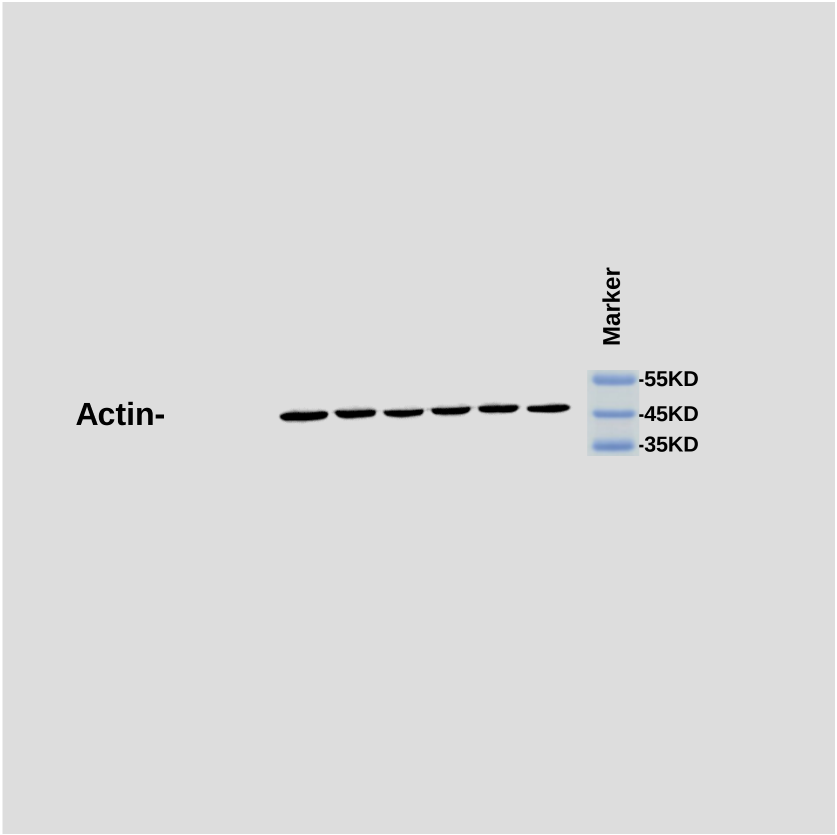

Figure 4I

Protein marker (#26616，Thermo)

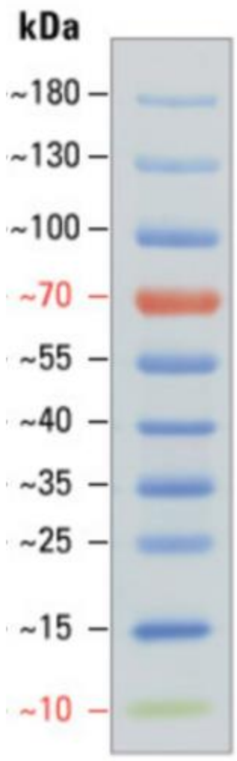

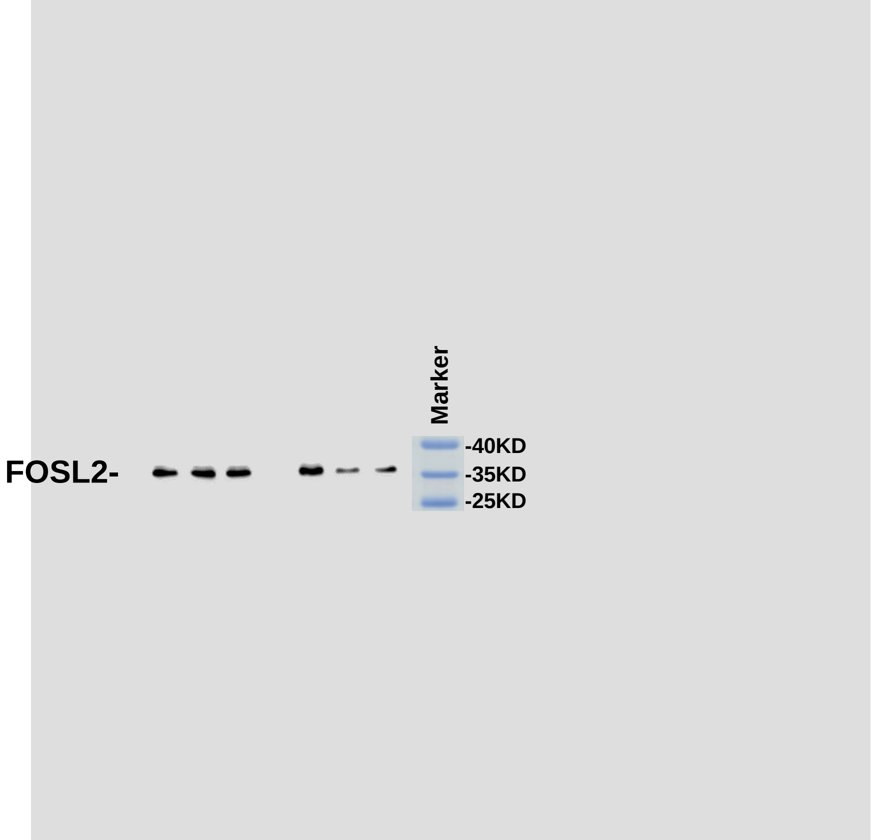

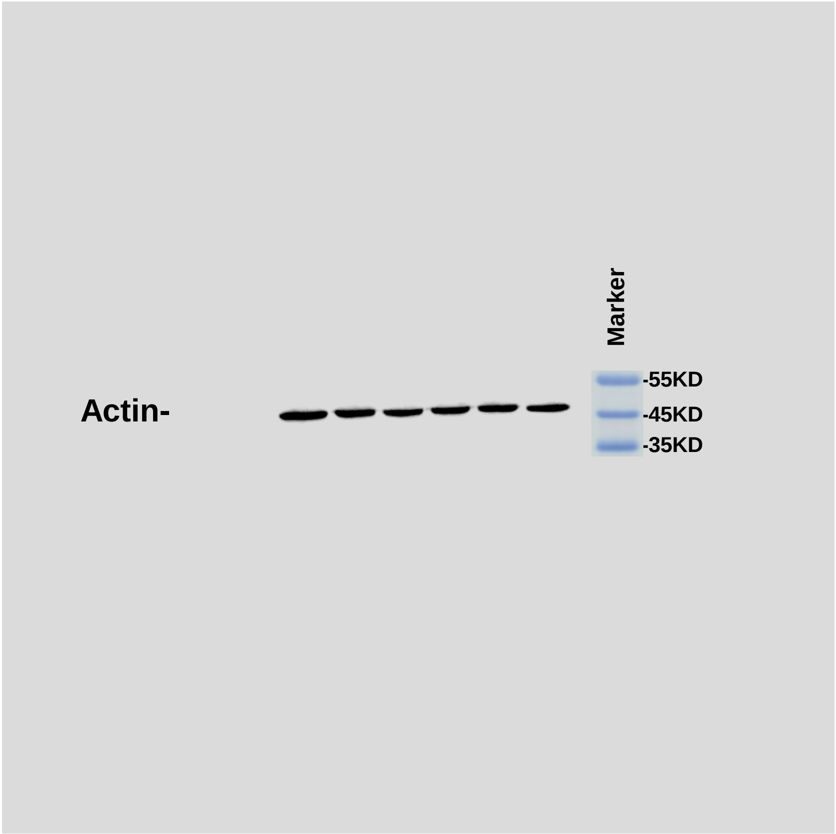

Figure 4K

Protein marker (#26616，Thermo)

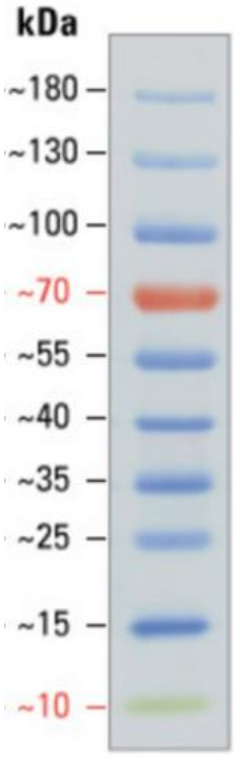

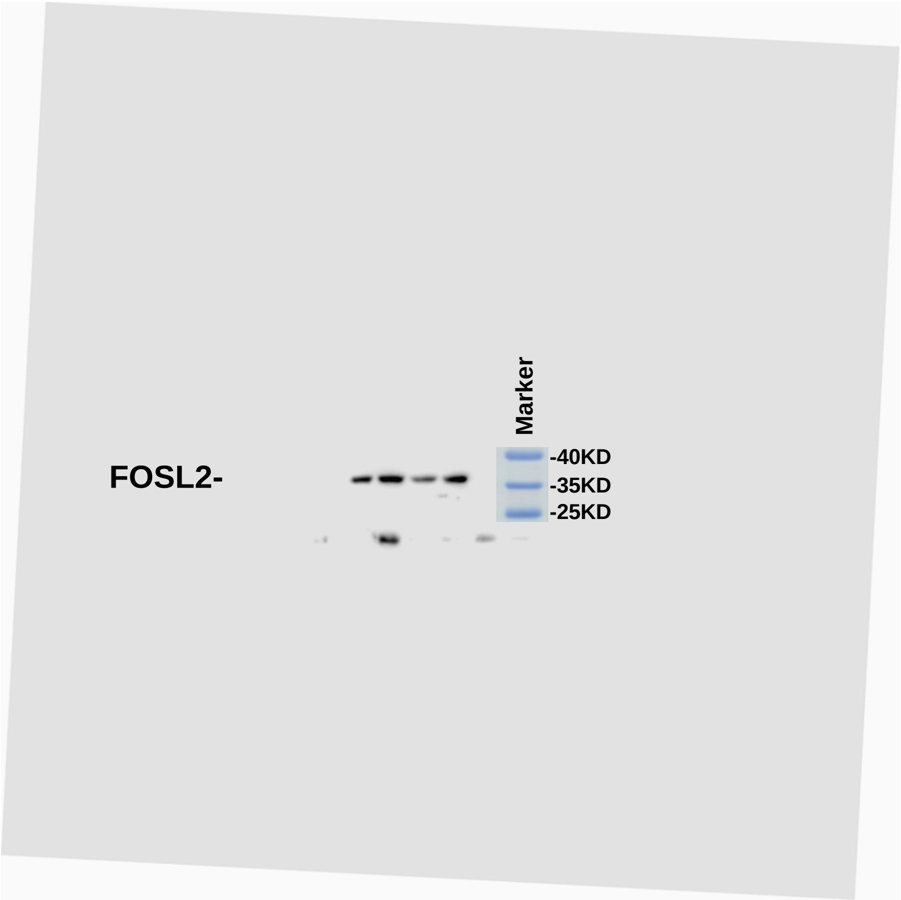

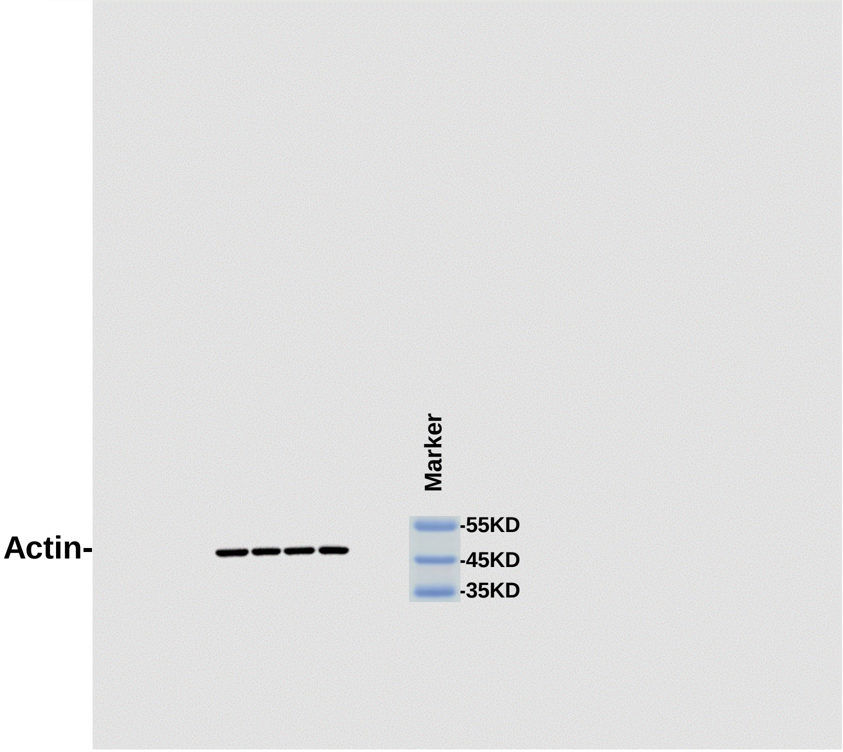

Figure 4L

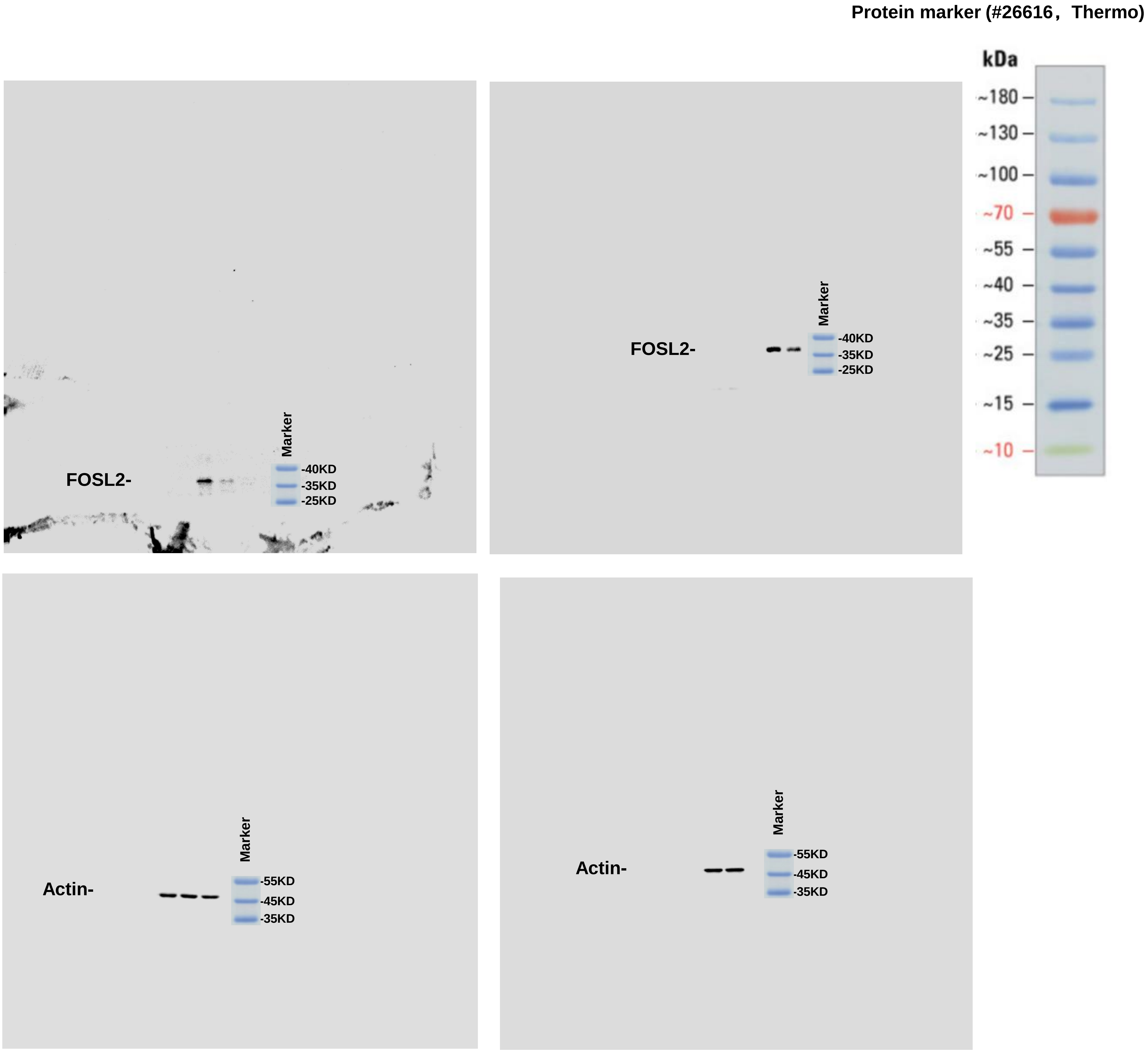
Protein marker (#26616，Thermo)
kDa
~180
~130
~100
~70
~55
~40
~35
~25
~15
~10
FOSL2-
Marker
-40KD
-35KD
-25KD
FOSL2-
Marker
-40KD
-35KD
-25KD
Actin-
Marker
-55KD
-45KD
-35KD
Actin-
Marker
-55KD
-45KD
-35KD

Figure 4L

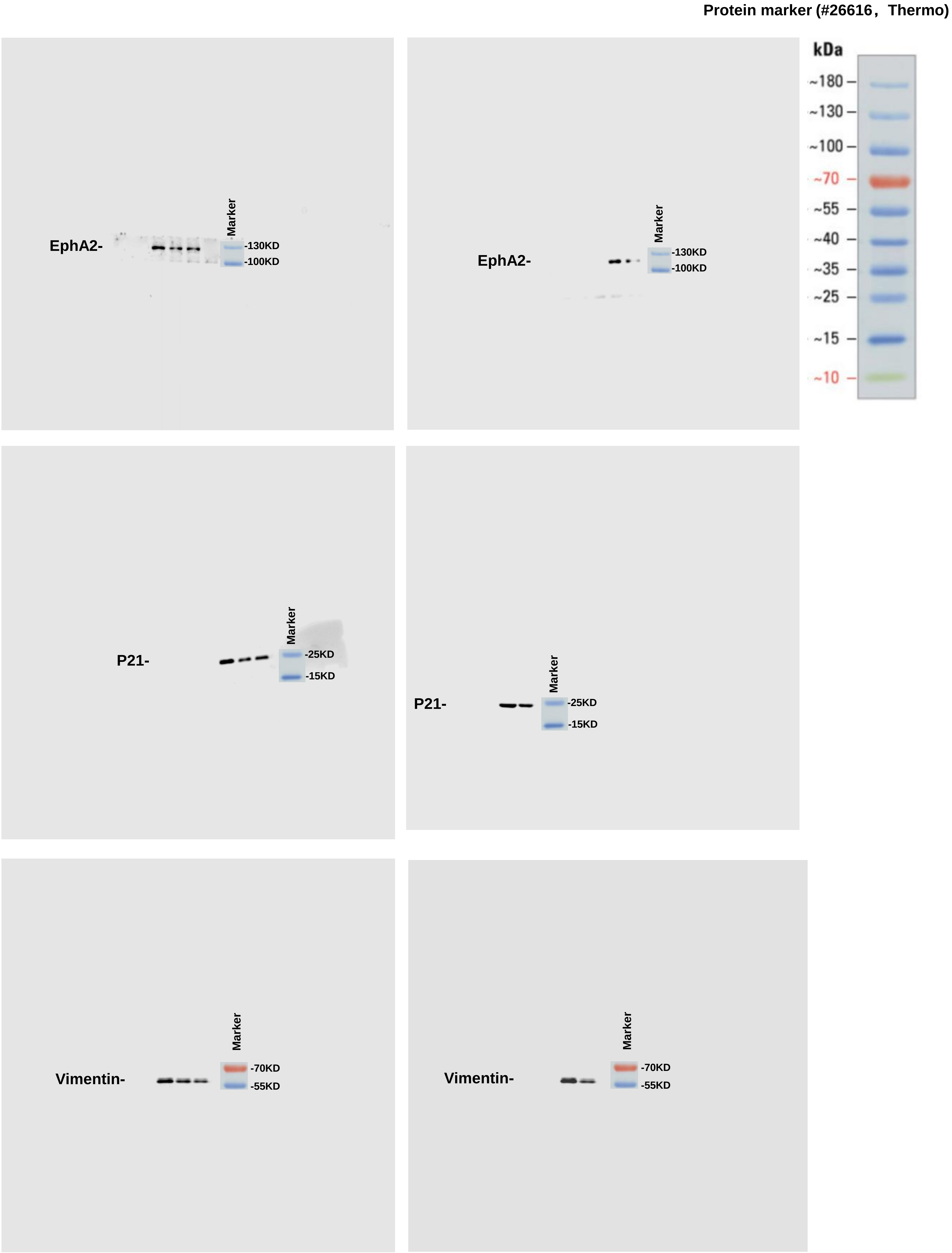

Protein marker (#26616，Thermo)
kDa
~180
~130
~100
~70
~55
~40
~35
~25
~15
~10
EphA2-
Marker
-130KD
-100KD
EphA2-
Marker
-130KD
-100KD
P21-
Marker
-25KD
-15KD
P21-
Marker
-25KD
-15KD
Vimentin-
Marker
-70KD
-55KD
Vimentin-
Marker
-70KD
-55KD

Figure 5D

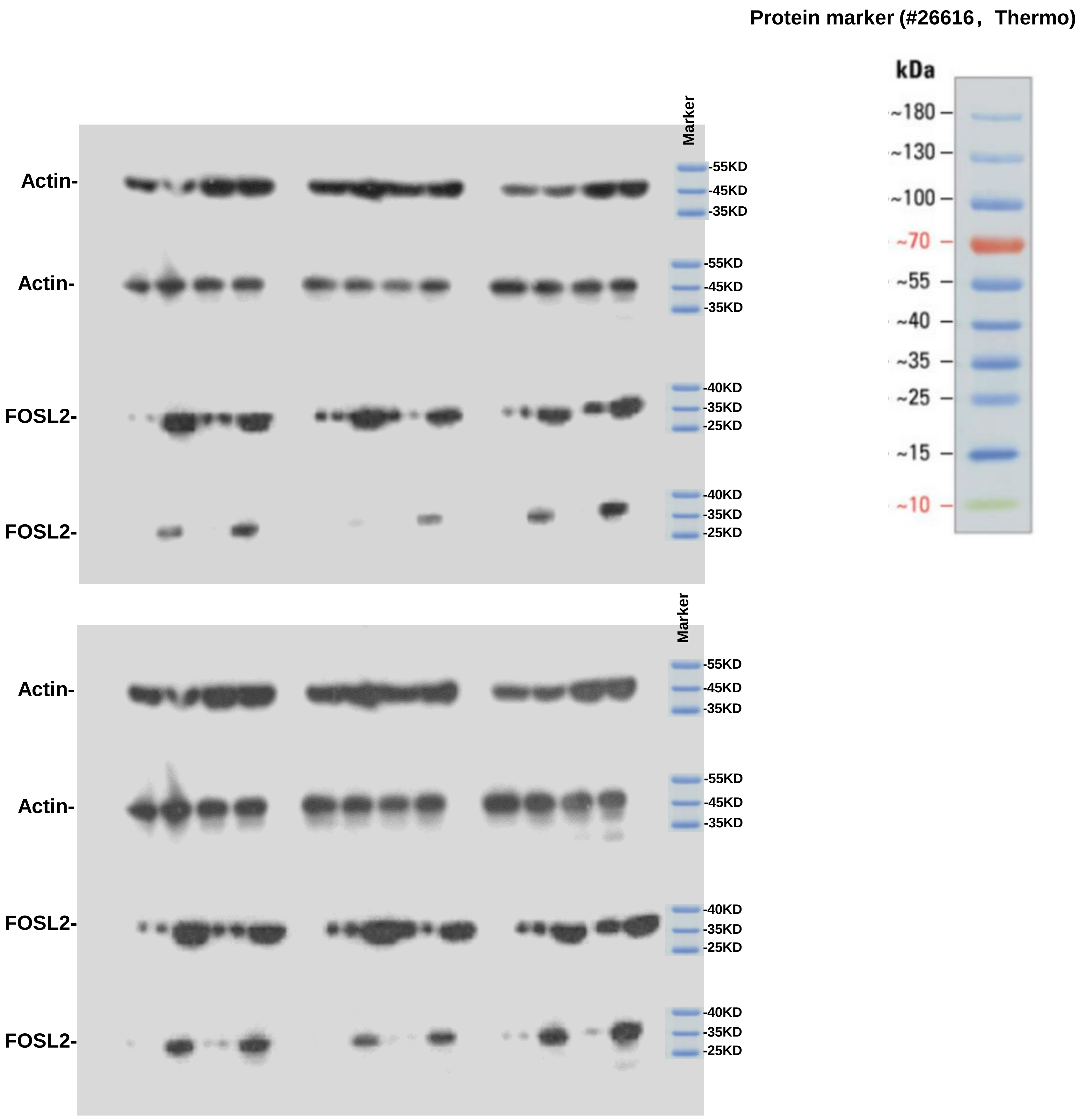

Figure 5F

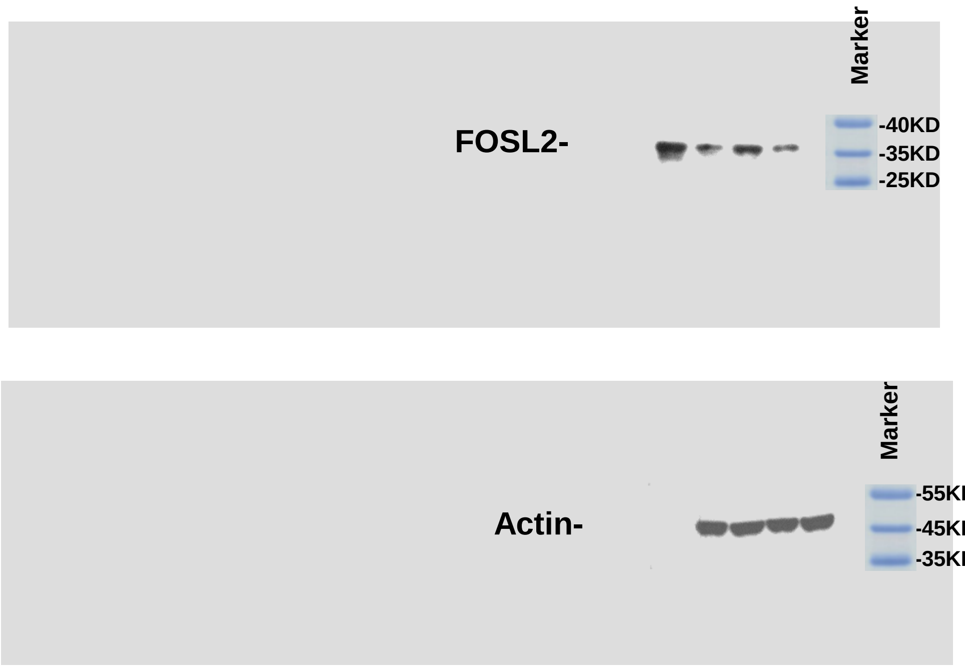

Figure 7A

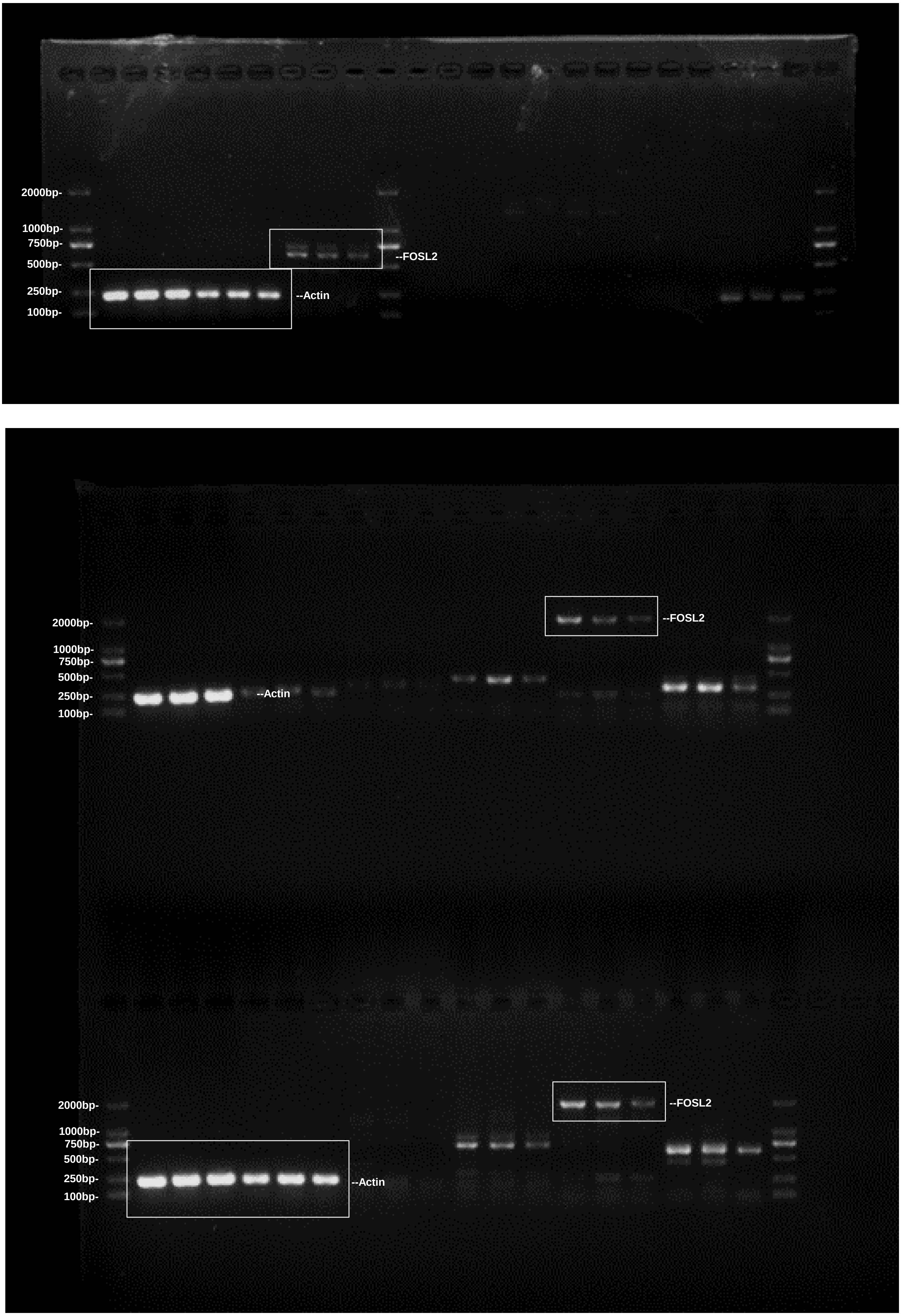
2000bp-
1000bp-
750bp-
500bp-
250bp-
100bp-
--FOSL2
--Actin
2000bp-
1000bp-
750bp-
500bp-
250bp-
100bp-
--FOSL2
--Actin
2000bp-
1000bp-
750bp-
500bp-
250bp-
100bp-
--FOSL2
--Actin

Figure 7D

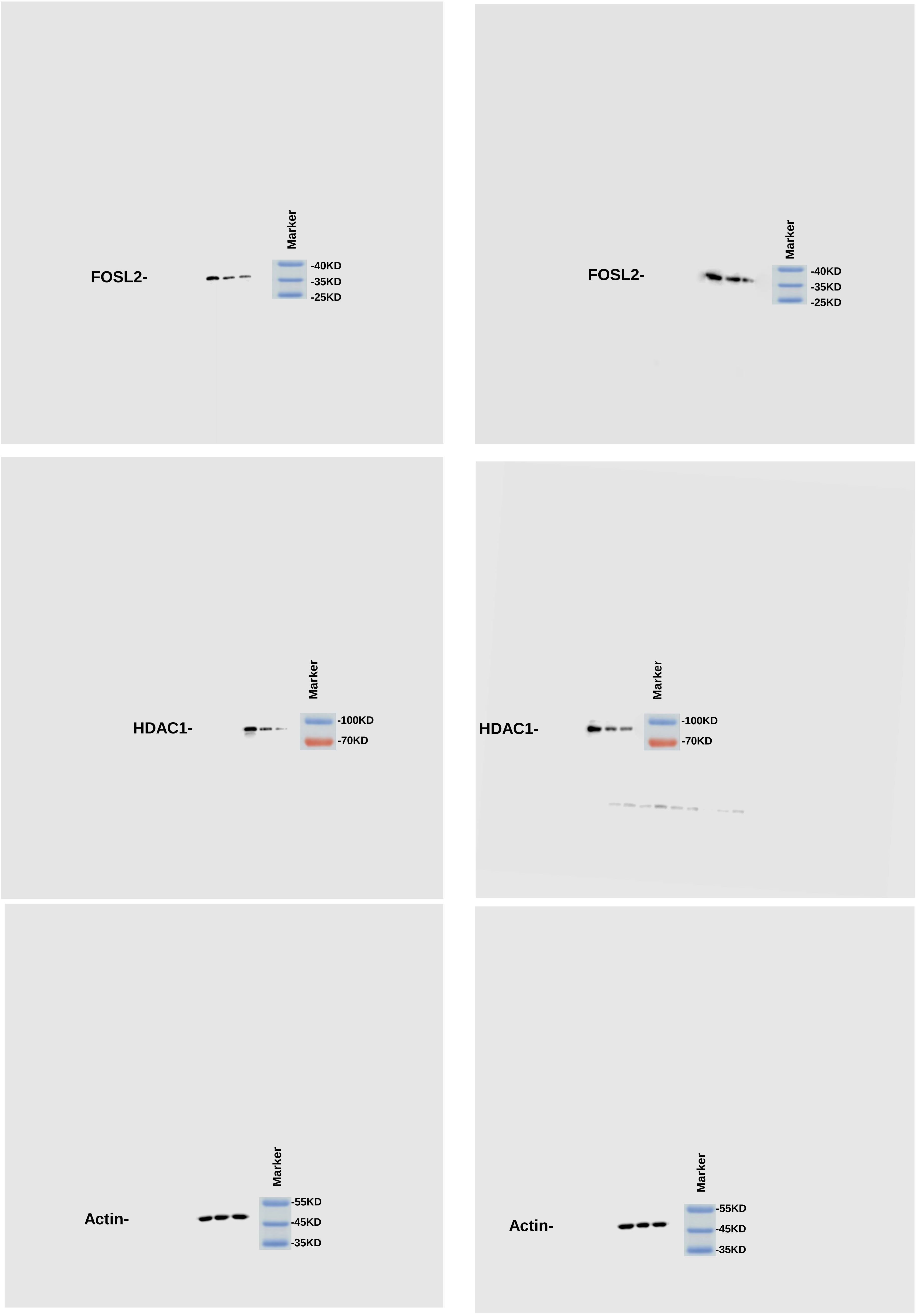

Marker
FOSL2-
-40KD
-35KD
-25KD
Marker
FOSL2-
-40KD
-35KD
-25KD
Marker
HDAC1-
-100KD
-70KD
Marker
HDAC1-
-100KD
-70KD
Marker
Actin-
-55KD
-45KD
-35KD
Marker
Actin-
-55KD
-45KD
-35KD

Figure 7F

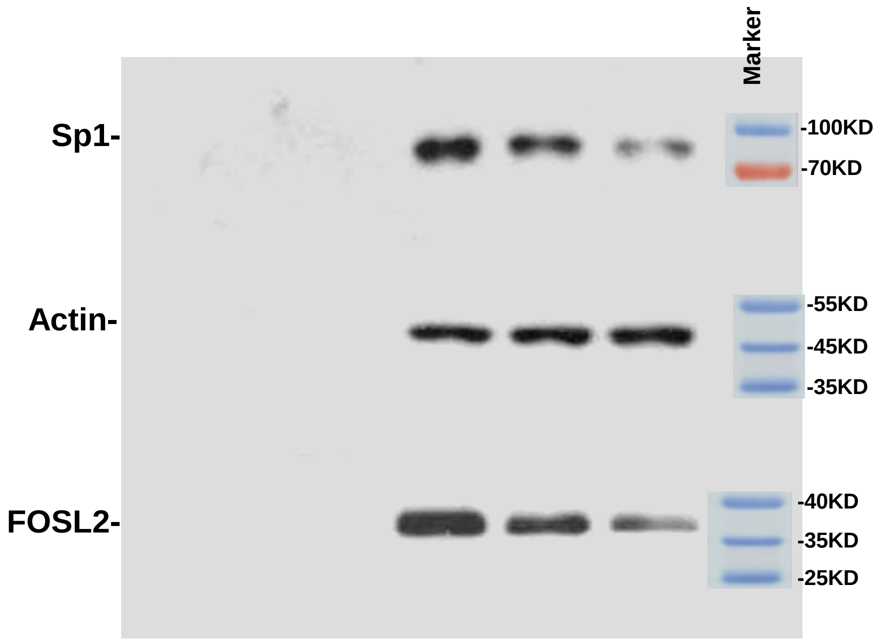

Protein marker (#26616，Thermo)

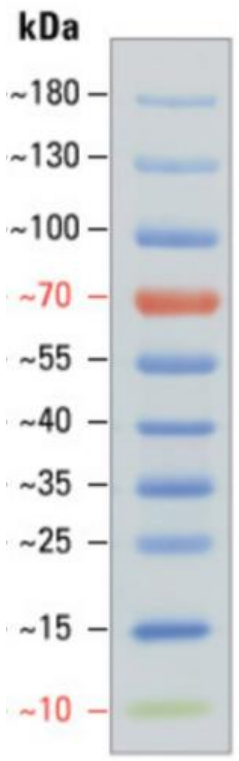

Figure 7J

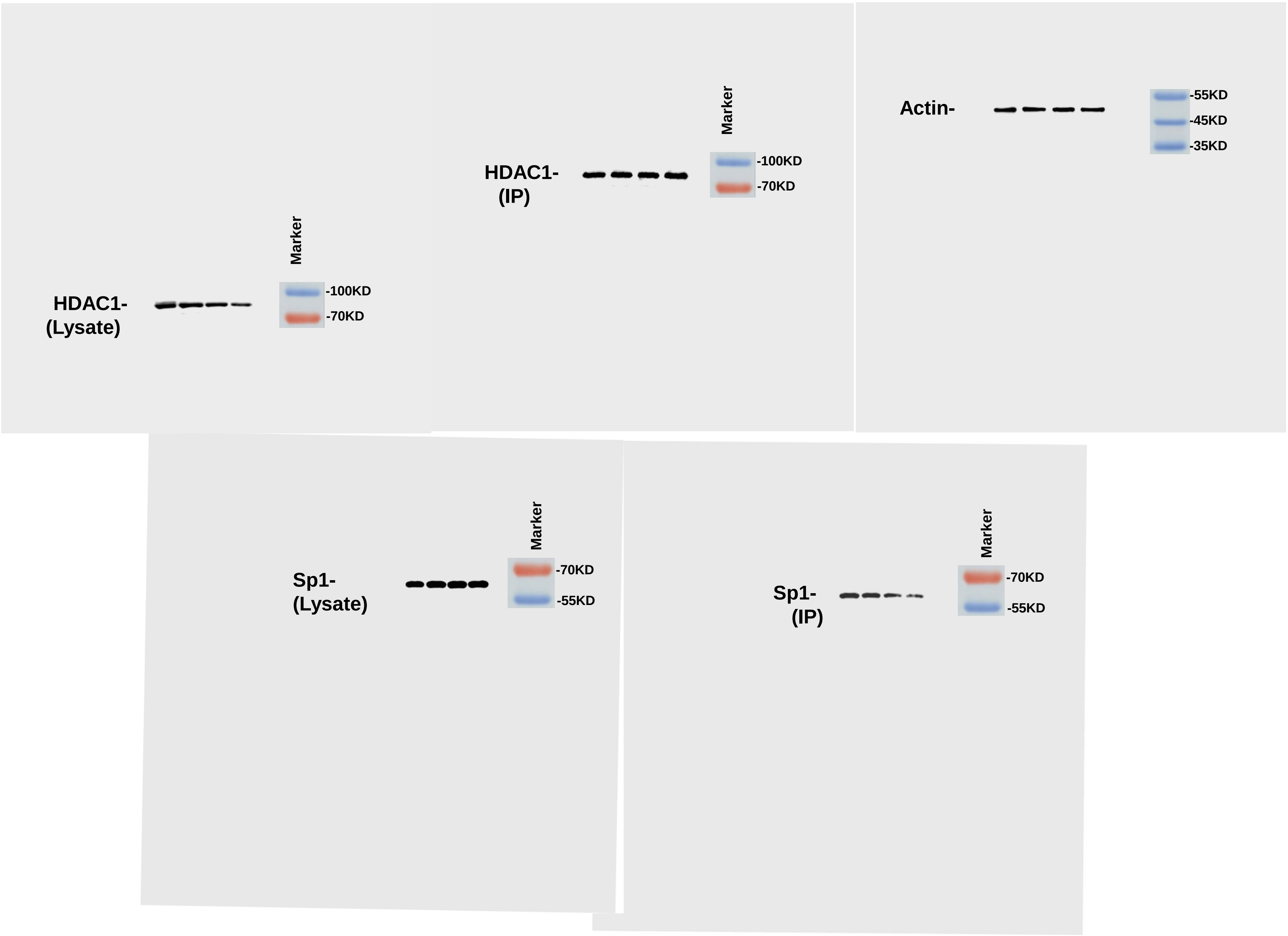

Figure 7M

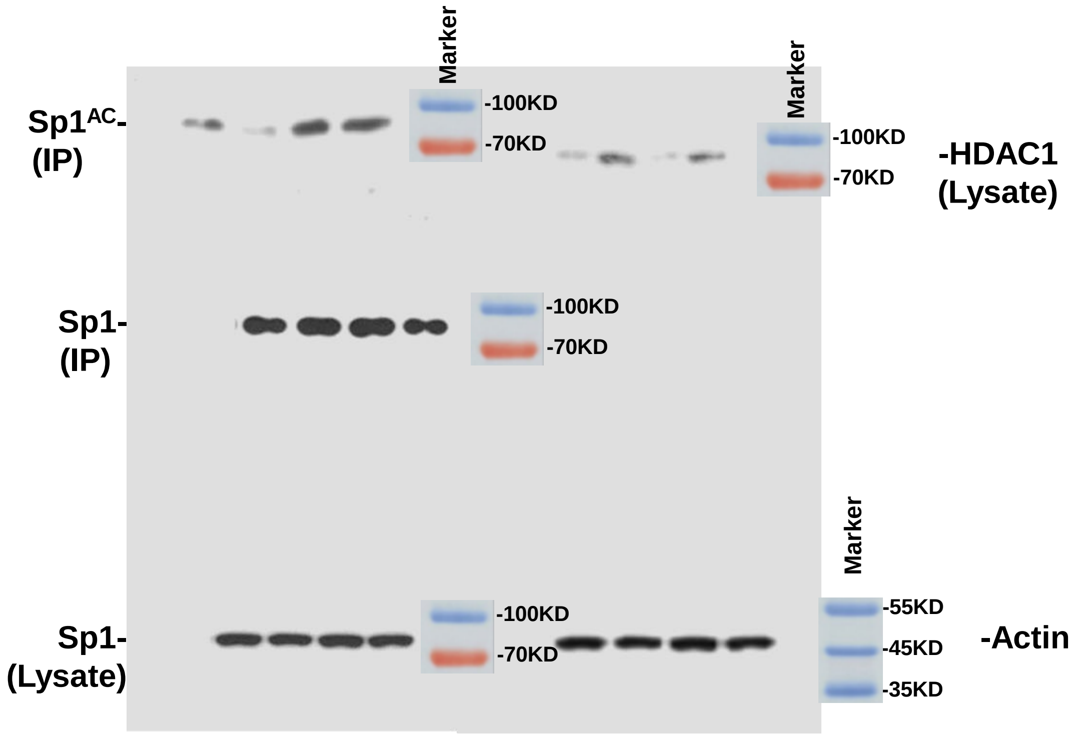

Protein marker (#26616，Thermo)

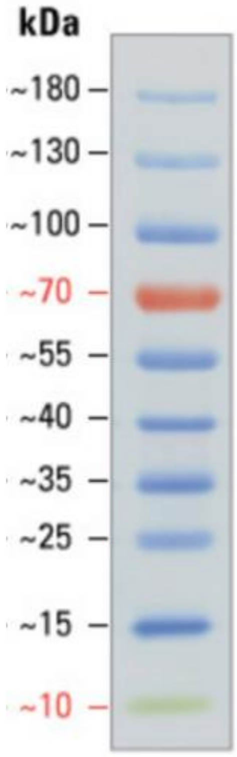

Figure 7O

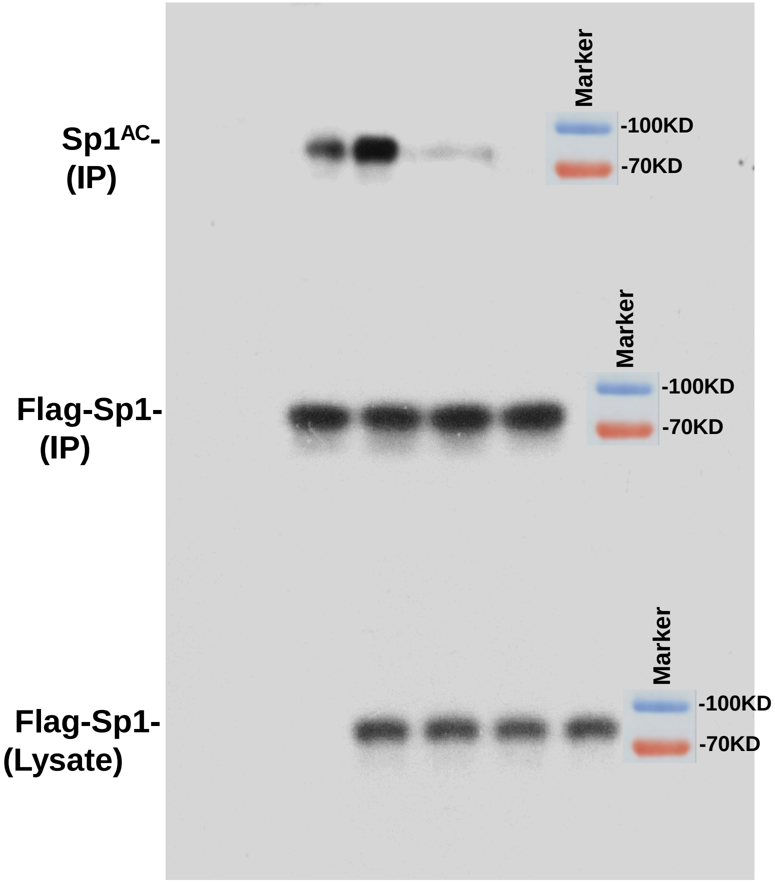

Protein marker (#26616，Thermo)

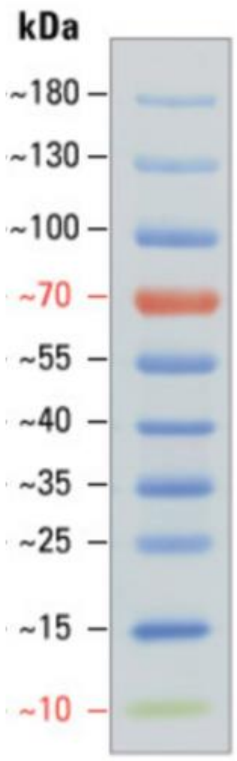

Figure 7Q

Protein marker (#26616，Thermo)

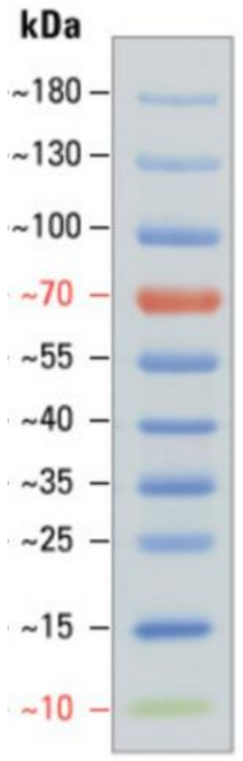

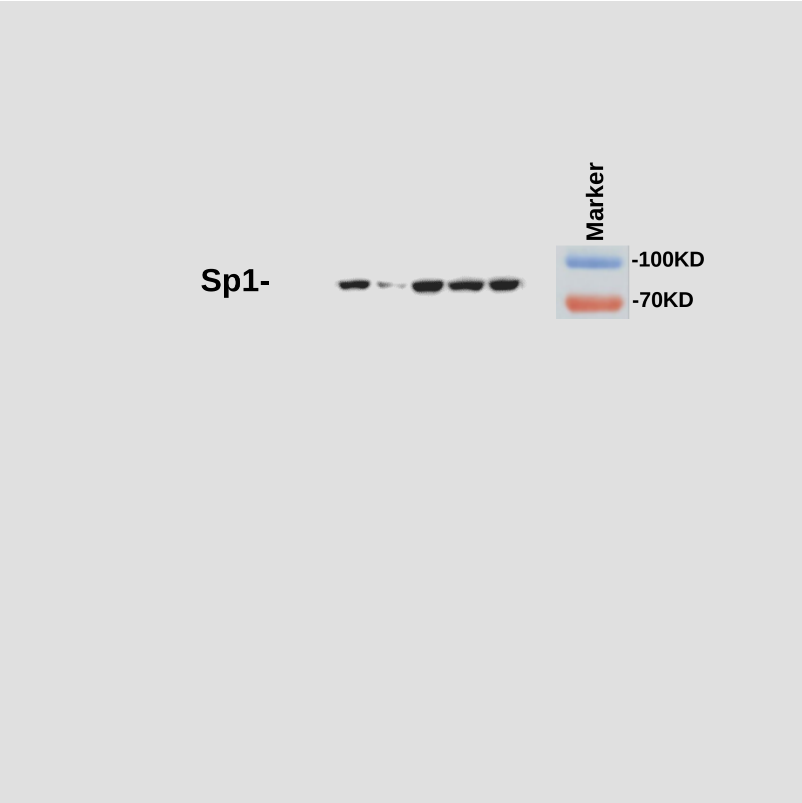

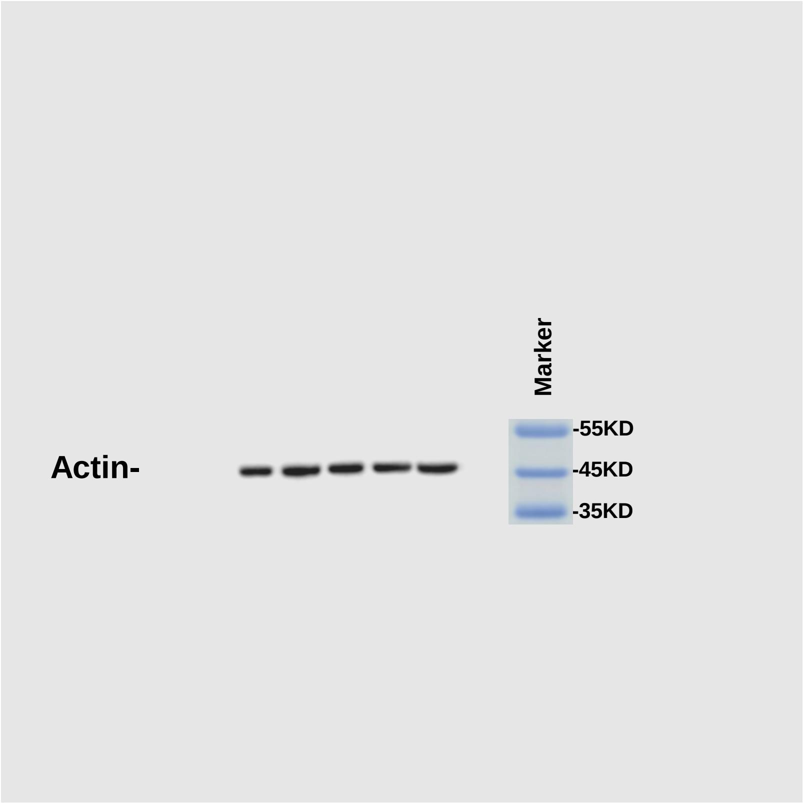

Supplement: Supplementary file 2 — Supplementary Material 2 [file 12964_2024_1733_MOESM2_ESM.pdf]
